# Diverse pathological lesions of primary aldosteronism and their clinical significance

**DOI:** 10.1038/s41440-020-00579-w

**Published:** 2021-01-12

**Authors:** Koshiro Nishimoto, Hironobu Umakoshi, Tsugio Seki, Masanori Yasuda, Ryuichiro Araki, Michio Otsuki, Takuyuki Katabami, Hirotaka Shibata, Yoshihiro Ogawa, Norio Wada, Masakatsu Sone, Shintaro Okamura, Shoichiro Izawa, Shozo Miyauchi, Takanobu Yoshimoto, Mika Tsuiki, Mitsuhide Naruse, JRAS Study Group, Takuyuki Katabami, Hisashi Fukuda, Yasushi Tanaka, Yoshiyu Takeda, Isao Kurihara, Hiroshi Itoh, Hironobu Umakoshi, Mika Tsuiki, Takamasa Ichijo, Norio Wada, Yui Shibayama, Takanobu Yoshimoto, Yoshihiro Ogawa, Junji Kawashima, Masakatsu Sone, Nobuya Inagaki, Katsutoshi Takahashi, Megumi Fujita, Minemori Watanabe, Yuichi Matsuda, Hiroki Kobayashi, Hirotaka Shibata, Kohei Kamemura, Michio Otsuki, Yuichi Fujii, Hiromi Rakugi, Koichi Yamamoto, Atsushi Ogo, Shintaro Okamura, Shozo Miyauchi, Toshihiko Yanase, Tomoko Suzuki, Takashi Kawamura, Mitsuhide Naruse, Tomikazu Fukuoka, Tatsuya Kai, Shoichiro Izawa, Yuichiro Yoshikawa, Shigeatsu Hashimoto, Masanobu Yamada, Ryuichi Sakamoto, Chiba Yoshiro

**Affiliations:** 1 Department of Uro-Oncology, Saitama Medical University International Medical Center, Saitama, 350-1241 Japan; 2 Department of Endocrinology and Metabolism, National Hospital Organization Kyoto Medical Center, Kyoto, 612-8555 Japan; 3 Department of Medical Education, School of Medicine, California University of Science and Medicine, Colton, CA USA; 4 Department of Pathology, Saitama Medical University International Medical Center, Hidaka, 350-1241 Japan; 5 Community Health Science Center, Saitama Medical University, Saitama, 350-0495 Japan; 6 Department of Metabolic Medicine, Osaka University Graduate School of Medicine, Osaka, 565-0871 Japan; 7 Division of Metabolism and Endocrinology, Department of Internal Medicine, St. Marianna University School of Medicine Yokohama City Seibu Hospital, Yokohama, 241-0811 Japan; 8 Department of Endocrinology, Metabolism, Rheumatology and Nephrology, Faculty of Medicine, Oita University, Yufu, 879-5593 Japan; 9 Department of Medicine and Bioregulatory Science, Graduate School of Medical Science, Kyushu University, Fukuoka, 812-8582 Japan; 10 Department of Molecular Endocrinology and Metabolism, Tokyo Medical and Dental University, Tokyo, 113-8510 Japan; 11 Department of Diabetes and Endocrinology, Sapporo City General Hospital, Sapporo, 060-8604 Japan; 12 Department of Diabetes, Endocrinology and Nutrition, Kyoto University, Kyoto, 606-8303 Japan; 13 Department of Endocrinology, Tenri Hospital, Tenri, 632-8552 Japan; 14 Division of Endocrinology and Metabolism, Tottori University Faculty of Medicine, Yonago, 683-8504 Japan; 15 Department of Internal Medicine, Uwajima City Hospital, Uwajima, Japan; 16 Division of Metabolism and Endocrinology, Department of Internal Medicine, St. Marianna University School of Medicine, Yokohama, Japan; 17 Department of Internal Medicine, Graduate School of Medical Science, Kanazawa University, Kanazawa, Japan; 18 Department of Endocrinology, Metabolism and Nephrology, School of Medicine Keio University, Tokyo, Japan; 19 Department of Endocrinology and Metabolism, Saiseikai Yokohamashi Tobu Hospital, Yokohama, Japan; 20 Department of Metabolic Medicine, Faculty of Life Science, Kumamoto University, Kumamoto University, Kumamoto, Japan; 21 Department of Diabetes, Endocrinology and Nutrition Kyoto University, Kyoto, Japan; 22 Division of Metabolism, Showa General Hospital, Tokyo, Japan; 23 Division of Nephrology and Endocrinology, The University of Tokyo, Tokyo, Japan; 24 Department of Endocrinology and Diabetes, Okazaki City Hospital, Okazaki, Japan; 25 Department of Cardiology, Sanda City Hospital, Sanda, Japan; 26 Division of Nephrology, Hypertension and Endocrinology, Nihon University School of Medicine, Tokyo, Japan; 27 Department of Cardiology, Akashi Medical Center, Akashi, Japan; 28 Department of Cardiology, JR Hiroshima Hospital, Hiroshima, Japan; 29 Department of Geriatric and General Medicine, Osaka University Graduate School of Medicine, Osaka, Japan; 30 Clinical Research Institute, National Hospital Organization Kyusyu Medical Center, Fukuoka, Japan; 31 Department of Endocrinology, Tenriyorozu Hospital, Tenri, Nara Japan; 32 Department of Endocrinology and Diabetes Mellitus, Faculty of Medicine, Fukuoka University, Fukuoka, Japan; 33 Department of Public Health, School of Medicine, International University of Health and Welfare, Narita, Japan; 34 Department of Preventive Services, Kyoto University School of Public Health, Kyoto, Japan; 35 Clinical Research Institute of Endocrinology and Metabolism, Kyoto Medical Center, National Hospital Organization, Kyoto, Japan; 36 Department of Internal Medicine, Matsuyama Red Cross Hospital, Matsuyama, Japan; 37 Department of Cardiology, Saiseikai Tondabayashi Hospital, Tondabayashi, Japan; 38 Department of Endocrinology and Metabolism, Tottori University Hospital, Tottori, Japan; 39 Department of Endocrinology and Diabetes Mellitus, Misato Kenwa Hospital, Misato, Japan; 40 Division of Nephrology, Hypertension, Endocrinology, and Diabetology/ Metabolism, Fukushima Medical University Hospital, Fukushima, Japan; 41 Department of Medicine and Molecular Science, Gunma University Graduate School of Medicine, Maebashi, Japan; 42 Department of Diabetes and Endocrinology, Saiseikai Fukuoka General Hospital, Fukuoka, Japan; 43 Endovascular Treatment Group, Mito Saiseikai General Hospital, Mito, Japan

**Keywords:** Primary aldosteronism, Adrenal venous sampling, Aldosterone

## Abstract

Primary aldosteronism (PA) is mainly clinically classified as unilateral aldosterone-producing adenoma (APA) or bilateral idiopathic hyperaldosteronism. Immunohistochemistry for aldosterone synthase reveals a diverse PA pathology, including pathological APA and aldosterone-producing cell clusters. The relationship between PA pathology and adrenalectomy outcomes was examined herein. Data from 219 unilaterally adrenalectomized PA cases were analyzed. Pathological analyses revealed diverse putative aldosterone-producing lesions. Postoperative biochemical outcomes in 114 cases (test cohort) were classified as complete success (*n* = 85), partial success (*n* = 19), and absent success (*n* = 10). Outcomes in the large and small PA lesion groups, rather than between PA lesion types, were compared at five threshold values for PA lesion sizes (2–6 mm with 1-mm increments) to streamline the results. The proportion of complete success was significantly higher in the large PA lesion group than in the small PA lesion group at the 5-mm threshold only. The proportion of absent success was significantly higher in the small PA lesion group than in the large PA lesion group at all thresholds. Univariate and multivariate analyses of the test cohort identified serum K as an independent predictive factor for the small PA lesion group, which was confirmed in the 105-case validation cohort. Chi-squared automatic interaction detector analysis revealed that the best threshold of serum K for predicting large PA lesions was 2.82 mEq/L. These results will be beneficial for treating PA in clinical settings because patients with low serum K levels and apparent adrenal masses on CT may be subjected to adrenalectomy even if the adrenal venous sampling test is unavailable.

## Introduction

Primary aldosteronism (PA) is the most common cause of endocrine hypertension, which significantly increases cardiovascular complications due to autonomous aldosterone production [1]. PA is diagnosed by confirmatory tests [2] and is primarily classified as unilateral PA, chiefly aldosterone-producing adenoma (APA, generally unilateral), or bilateral PA (also called idiopathic hyperaldosteronism) [3]. The former is often curable by unilateral adrenalectomy, whereas the latter is mostly treated by lifelong mineralocorticoid receptor antagonists [4].

We previously reported an immunohistochemistry protocol for aldosterone synthase (CYP11B2) that distinguishes CYP11B2 from a cortisol-synthesizing enzyme (steroid 11β-hydroxylase, CYP11B1) [5]. On hematoxylin and eosin (H&E) staining, APA, and adrenal incidentaloma are both characterized by a heterogeneous mixture of compact cells and large lipid-rich cells [5]. However, immunohistochemistry reveals that APA consists of CYP11B2-positive cells, CYP11B1-positive cells, and double negative cells, whereas non-functional adenomas only comprise CYP11B1-positive cells and double negative cells and did not harbor CYP11B2-positive cells. Thus, immunohistochemistry enables pathological discrimination between APA and incidentaloma through the detection of CYP11B2-positive cells [5].

CYP11B2 immunohistochemistry also identifies subcapsular aldosterone-producing cell clusters (APCCs, 0.2–1 mm in length) in most normal adult adrenal glands, which strongly express CYP11B2 [5]. Moreover, possible APCC-to-APA transitional lesions (pAATLs) have been identified in some PA adrenal glands, which consist of a subcapsular APCC-like portion and an inner APA-like portion [6, 7]. Somatic genetic mutation statuses in APCCs [8, 9] and pAATLs [6, 10] as well as the in situ localization of aldosterone in these lesions are consistent with the hypothesis that APCCs and pAATLs cause PA in addition to APA [11].

In recent years, increasing information has been gathered on the pathology and pathophysiology of PA; however, it remains unclear whether any relationship exists between PA pathology and unilateral adrenalectomy outcomes. Therefore, in this retrospective study, we attempted to elucidate the relationship between PA pathology and unilateral adrenalectomy outcomes, with a focus on the size of PA lesions and the preoperative predictive factor(s) of PA pathology, by generating the largest catalog of PA pathology and clinical data from 219 PA cases in 11 Japanese institutions.

## Methods

### Japan Research Projects for Rare/Intractable Adrenal Diseases (JRAS)

This study was conducted as part of the multi-institutional JRAS study. JRAS (http://www.adrenal.jp/en) is a consortium of 28 Japanese institutions as of September 2018, which was registered in the University Hospital Medical Information Network Clinical Trials Registry [UMIN-CTR] # 18756.

### Ethics and case selection

Investigators in 11 JRAS-participating institutions acquired Institutional Review Board (IRB) approval and selected 242 cases from their own institutions (Supplementary Table 1). Glass slides with adrenal tissue sections were sent to the Saitama Medical University International Medical Center (SIMC), and pathological analyses were performed at the SIMC under the SIMC IRB approval (Supplementary Fig. 1 and Supplementary Data 1 at https://humandbs.biosciencedbc.jp/en/hum0185-v1-st1). Details of ethical approval and case selection are described in the Supplementary Materials and Methods.

### Removal of 20 pathological samples

Among the 242 selected cases, 20 were excluded from subsequent statistical analyses due to inadequate slide sample conditions, as detailed in the Supplementary Materials and Methods.

### Initial pathological diagnosis

Initial pathological diagnoses of the remaining cases (*n* = 222) were performed by one of the authors (KN) (“Initial pathological diagnosis” in Supplementary Table 1). Detailed methods are shown in the Supplementary Materials and Methods.

### Isolation of clinical data

Clinical data for the 222 cases with initial pathological diagnoses were extracted from the JRAS database: preoperative physical data including age on the day of adrenalectomy (age, year), sex, and body mass index (BMI, kg/m^2^); the intensity of antihypertensives (see below and Supplementary Materials and Methods); systolic and diastolic blood pressure values while on antihypertensives with a defined daily dose; computed tomography (CT) data including maximum tumor length (mm); laboratory data including the estimated glomerular filtration rate (eGFR, mL/min/1.73 m^2^), plasma aldosterone concentration (PAC, pg/mL), plasma renin activity (PRA, ng/mL/hr); and adrenal venous sampling (AVS) data after intravenous stimulation using synthetic adrenocorticotropic hormone (ACTH). eGFR was calculated as follows: 194 × (serum creatinine level [mg/dL] − 1.094) × (age [years] − 0.287) × 0.739 [12]. Based on the AVS data, the lateralized ratio (LR) was calculated using the PAC and plasma cortisol concentrations [PCCs, µg/dL] as follows: LR = (PAC to PCC ratio on the dominant side)/(PAC to PCC ratio on the nondominant side). At this stage of the analysis, we removed three additional cases from the statistical study because one had PA concomitant with Cushing’s syndrome (Case 78, Supplementary Table 1) and two lacked any clinical information (Cases 111 and 176).

### Intensity calculation of hypertensive agents

The preoperative use of anti-hypertensive agents by each patient was quantified as the intensity of antihypertensives using a previously reported method [13], as detailed in the Supplementary Methods (Supplementary Table 2 [calculation of defined daily doses of antihypertensive agents] and Supplementary Data 2 [package inserts of azelnidipine, alacepril, bunazosin, and guanabenz, available only in Japanese: suggested daily doses are highlighted with blue boxes, and the English translations of the dosages is provided]). Detailed calculation methods are shown in the Supplementary Materials and Methods.

### Definition of surgical outcomes

The surgical outcomes (i.e., biochemical and clinical outcomes) of PA were evaluated ~6 months after adrenalectomy following a recently reported protocol [13]. In brief, biochemical outcomes were defined as “complete success” when patients had an aldosterone-to-renin ratio (ARR) <200 and serum K level >3.5 mEq/L, “partial success” when the postoperative ARR was <50% of the presurgical ARR but still >200 and serum K levels were >3.5 mEq/L, and “absent success” for the remaining patients. In the present study, the postoperative data from confirmatory tests, including the saline infusion test and captopril challenge test, were not used for outcome evaluation. Clinical outcome were defined as “complete success” when the patient had normal blood pressure without the aid of antihypertensive medication, “partial success” when the patient had the same blood pressure as before surgery with less antihypertensive medication or a reduction in blood pressure with either the same amount or less antihypertensive medication, and “absent success” for the remaining patients.

### Statistical analysis

The normality of continuous variables was tested using the Shapiro–Wilk normality test. Continuous variables with normal and non-normal distributions are reported as means with standard deviations (mean ± SD) and medians with interquartile ranges (median [IQRs]), respectively. Differences between two unpaired groups with normal and non-normal distributions were tested by the unpaired *t*-test and Mann–Whitney *U* (MW-*U*) test, respectively. Categorical variables between two groups were compared by Fisher’s exact test. Multivariable logistic regression analysis was used to identify the associated factors for distinguishing small lesions from large lesions (also see “Results” section). Chi-squared automatic interaction detector (CHAID) analysis was performed to identify the best cutoff value. *p* values < 0.05 were considered to be significant. Statistical analyses, except for the CHAID analysis, were performed using SigmaPlot version 14.0 (Systat Software, San Jose, CA). The CHAID analysis was performed using IBM SPSS Software version 25.

## Results

### Catalog of PA lesions

**Fig. 1:**
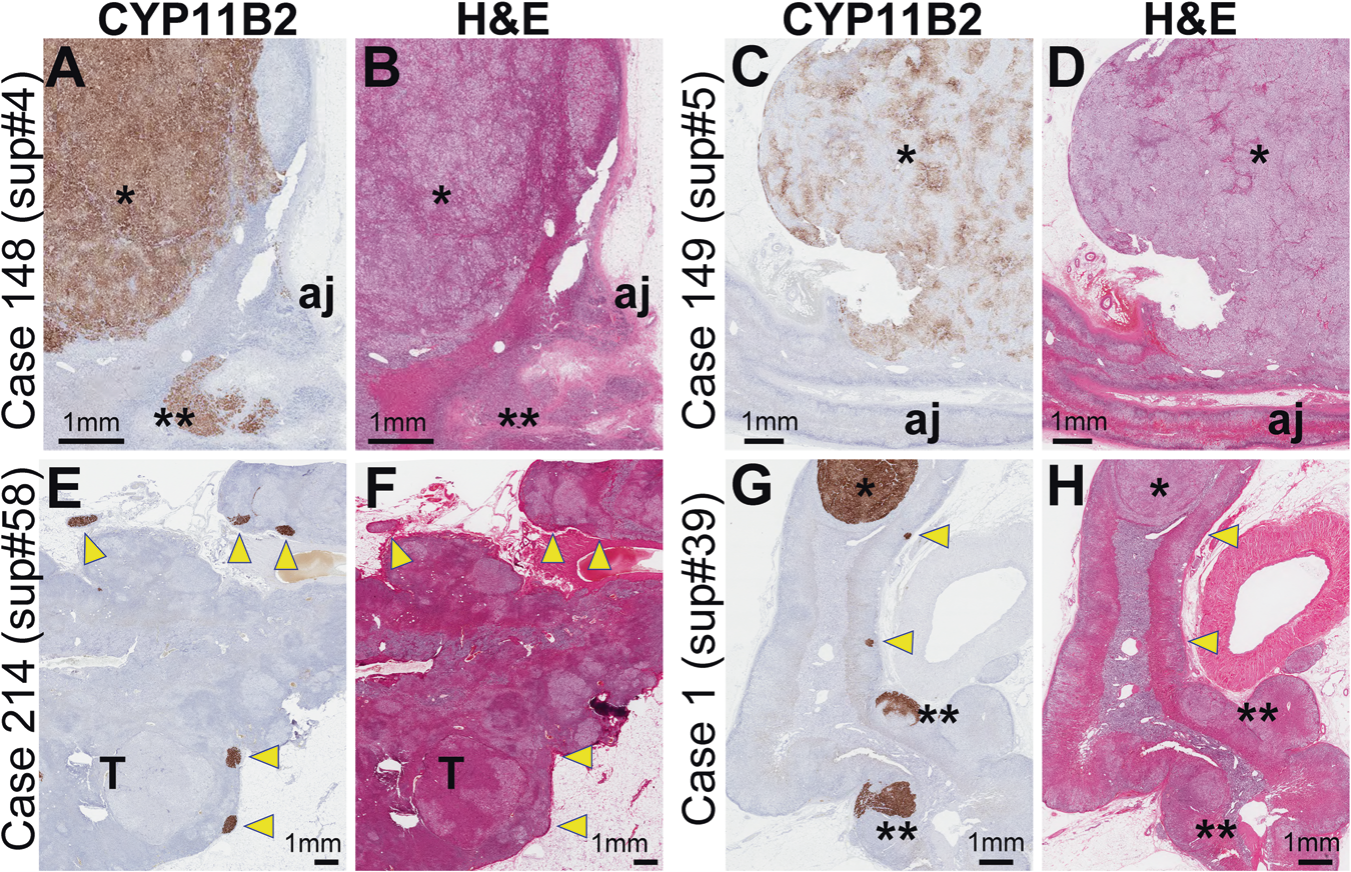
Representative images of aldosterone-producing lesions. **A**–**B**, **C**–**D**, **E**–**F**, and **G**–**H** are representative images from Cases 148 (sup #4), 149 (#5), 214 (#58), and 1 (#39), respectively. **A**, **C**, **E**, and **G** Immunohistochemistry for CYP11B2. **B**, **D**, **F**, and **H** Hematoxylin & eosin staining of serial sections of **A**, **C**, **E**, and **G**, respectively. Symbols * (asterisk), yellow arrowheads, and ** (double asterisk) indicate aldosterone-producing adenoma (APA), aldosterone-producing cell clusters (APCCs), and possible APCC-to-APA transitional lesions, respectively. T: A nonfunctional tumor (incidentaloma)

Initial pathological analyses revealed diverse types of putative aldosterone-producing lesions (Fig. 1 and Supplementary Table 1): APAs that strongly (e.g., Case 148, asterisk in Fig. 1A, B) or unevenly/weakly (e.g., asterisk in Case 149, Fig. 1C, D) expressed CYP11B2, APCCs (e.g., Case 214, yellow arrowheads in Fig. 1E, F), and pAATLs (e.g., Case 1, Fig. 1G, H) (Supplementary Table 1 and Supplementary Fig. 1; all original images may be downloaded from https://humandbs.biosciencedbc.jp/en/hum0185-v1-st1 [Supplementary Data 1]). CYP11B2 positivity was calculated using the Aperio Positive Pixel Count Algorithm as we previously reported [7] and ranged between 4.34 and 62.5% (Supplementary Fig. 1 and “CYP11B2 positivity [per case, CYP11B2 staining #1 and #2]” column in Supplementary Table 1). Accordingly, the catalog of PA lesions we created for the present study included a wide range of lesion types, CYP11B2 positivities, and sizes.

### Cases with CYP11B2-positive PA lesions larger than 5 mm were more unilateral than those with lesions smaller than 5 mm

We investigated whether the sizes of the CYP11B2-positive PA lesions on stained slides were associated with postoperative clinical outcomes. Lesions did not include non-functional adenomas because those were not positive for CYP11B2. To perform the statistical analyses, we classified each case into a large or small PA lesion group based on the most dominant lesion for different threshold values (between 2 and 6 mm in 1-mm increments, Supplementary Table 1) as follows. For example, Cases 148 and 149 were classified into the large PA lesion group for all threshold values because the sizes of the most dominant lesions were >6 mm (asterisks in Fig. 1A–D), although Case 148 harbored an additional smaller lesion (double asterisks in Fig. 1A, B). Case 214 was classified into the small PA lesion group for all threshold values because the largest lesion was smaller than 2 mm (Fig. 1E, F). Case 1 was classified into the large and small PA lesion groups for threshold values of 2–3 mm and 4–6 mm, respectively, because the size of the largest lesion was 3.2 mm. Surgical outcomes were compared between the large and small PA lesion groups at each of the five threshold values.

We hypothesized that unilateral adrenalectomy is more effective for the large PA lesion group at a certain threshold value based on our extensive experience with pathological analysis. We previously found that cases with large PA lesion(s) generally had fewer smaller lesions in their adjacent adrenal glands and were more likely to be unilateral in nature than those with small PA lesions, which may have led to better surgical outcomes in these cases.

Therefore, we compared the percentages of complete success (vs. partial and absent success percentages) and absent success (vs. complete and partial success percentages) between the large and small PA lesion groups at each of the five threshold values (2–6 mm with 1-mm increments). Among the 219 cases, postoperative biochemical outcomes were available in 114 (test cohort: complete success [*n* = 85], partial success [*n* = 19], absent success [*n* = 10]). Among these cases, the percentage of complete success was significantly higher in the large PA lesion group (80.5%) than in the small PA group (55.6%) only when the threshold was 5 mm (*p* = 0.02, Fisher’s exact test, Table 1). The small PA lesion group at all thresholds (2–6 mm) showed significantly higher percentages of absent success than the large PA lesion group (22.6–28.0% vs. 3.4–5.3%, *p* < 0.05 for all thresholds, Table 1). All cases in the test cohort underwent AVS; however, data were not available for 24 cases, presumably due to the lack of ACTH stimulation, failed AVS, or missing data. We performed analyses using the data from the test cohort with AVS data (*n* = 90, “test cohort with AVS data” in Table 1) and confirmed that the results obtained were similar to the data of the entire test cohort. These results confirmed that unilateral adrenalectomy more effectively improved biochemical outcomes in the large PA lesion group when the threshold value was ≥5 mm.

**Table 1:** Biochemical outcomes between small and large PA lesion groups with different cut-offs for the maximum lesion size in pathology

	Test cohort (*n* =114)						Test cohort with adrenal venous sampling data (*n* = 90)					
Group (cut-off for the maximum lesion size)	Complete success	Partial and absent success	*p*	Complete and partial success	Absent success	*p*	Complete success	Partial and absent success	*p*	Complete and partial success	Absent success	*p*
L-PAL (>6 mm)	*66* (*79.5%*)	*17* (*20.5%*)	*0.06*	**80 (96.4%)**	**3 (3.6%)**	**0.004**	*52* (*77.6%*)	*15* (*22.4%*)	*0.06*	**64 (95.5%)**	**3 (4.5%)**	**0.02**
S-PAL (<6 mm)	*19* (*61.3%*)	*12* (*38.7%*)		**24 (77.4%)**	**7 (22.6%)**		*13* (*56.5%*)	*10* (*43.5%*)		**18 (78.3%)**	**5 (21.7%)**	
L-PAL (>5 mm)	**70 (80.5%)**	***17*** **(19.5%)**	**0.02**	**84 (96.6%)**	**3 (3.4%)**	**0.002**	**55 (78.6%)**	**15 (21.4%)**	**0.02**	**67 (95.7%)**	**3 (4.3%)**	**0.01**
S-PAL (<5 mm)	**15 (55.6%)**	***12*** **(44.4%)**		**20 (74.1%)**	**7 (25.9%)**		**10 (50.0%)**	**10 (50.0%)**		**15 (75.0%)**	**5 (25.0%)**	
L-PAL (>4 mm)	*70* (*78.7%*)	*19* (*21.3%*)	*0.07*	**86 (96.6%)**	**3 (3.4%)**	**<0.001**	*55* (*76.4%*)	*17* (*23.6%*)	*0.087*	**67 (95.7%)**	**3 (4.3%)**	**0.01**
S-PAL (<4 mm)	*15* (*60.0%*)	*10* (*40.0%*)		**18 (72.0%)**	**7 (28.0%)**		*10* (*55.6%*)	*8* (*44.4%*)		**15 (75.0%)**	**5 (25.0%)**	
L-PAL (>3 mm)	*71* (*78.0%*)	*20* (*22.0%*)	*0.11*	**87 (95.6%)**	**4 (4.4%)**	**0.004**	*56* (*76.7%*)	*17* (*23.3%*)	*0.07*	**70 (95.9%)**	**3 (4.1%)**	**0.005**
S-PAL (<3 mm)	*14* (*60.9%*)	*9* (*39.1%*)		**17 (73.9%)**	**6 (26.1%)**		*9* (*52.9%*)	*8* (*47.1%*)		**12 (70.6%)**	**5 (29.4%)**	
L-PAL (>2 mm)	*73* (*76.8%*)	*22* (*23.2%*)	*0.25*	**90 (94.7%)**	**5 (5.3%)**	**0.01**	*58* (*75.3%*)	*19* (*24.7%*)	*0.177*	**73 (94.8%)**	**4 (5.2%)**	**0.01**
S-PAL (<2 mm)	*12* (*63.2%*)	*7* (*36.8%*)		**14 (73.7%)**	**5 (26.3%)**		*7* (*53.8%*)	*6* (*46.2%*)		**9 (69.2%)**	**4 (30.8%)**	

S-PAL and L-PAL represent the small and large PA lesion groups, respectively. Numbers (percentages) indicate the number of cases (percentage of the number of cases). *p* values were calculated by Fisher’s exact tests. Bold and italicized characters indicate data with *p* values < 0.05 and ≥0.05, respectively

Although similar analyses were performed using clinical outcomes, no significant differences were observed between the large and small PA lesion groups at any threshold (Supplementary Table 3). Additional comparisons in the test cohort showed that among patients with available data, the duration of hypertension was longer in the large PA lesion group (10.0 [5.0–13.0] years, *n* = 83) than in the small PA lesion group (3.0 [1.0–12.0] years, n = 25, *p* = 0.028, MW-U). The longer hypertensive status may have contributed to the lack of differences in clinical outcomes, possibly due to the accumulation of damage in the vasculature and other organs.

Similarly, CYP11B2 positivity levels were associated with biochemical outcomes but not with clinical outcomes. CYP11B2 positivity was significantly higher in the large PA lesion group (33.1 ± 1.4%) than in the small PA lesion group (3.26 ± 3.97%, *p* < 0.001, unpaired *t*-test). In terms of biochemical outcomes, CYP11B2 positivity was significantly higher in patients who achieved complete success (30.3 [15.9–39.9]%) than in those who did not achieve success (1.8 [0.8–28.6]%, *p* = 0.04, Kruskal–Wallis one-way analysis of variance on ranks with Dunn’s post hoc test); however, no significant difference was observed in CYP11B2 positivity between patients who achieved complete success and partial success (0.231 [0.121–0.435], Supplementary Fig. 2A). Regarding clinical outcomes, no significant differences were noted between patients achieving complete success (31.5 [20.7–41.0]%), partial success (27.9 [14.8–38.9]%), and absent success 30.1 [10.3–48.1]%, *p* = 0.808, Kruskal–Wallis one-way analysis of variance on ranks, Supplementary Fig. 2B). Thus, patients with higher CYP11B2 positivity levels showed better biochemical outcomes but similar clinical outcomes.

These results appear to support the hypothesis that unilateral adrenalectomy is more effective for the large PA lesion group, presumably due to the lower rate of bilateral PA lesions in this group, and suggest that the size of the pathological lesions influences biochemical outcomes more than clinical outcomes after adrenalectomy.

### Identification of preoperative factors associated with large (≥5 mm) PA lesions

To identify preoperative factors associated with the sizes of PA lesions, preoperative clinical data were compared between the large (≥5 mm) and small (<5 mm) PA lesion groups in the same test cohort (*n* = 114, Table 2). Age (years), sex, side of adrenalectomy, body mass index (kg/m^2^), and eGFR were not significantly different between the groups. The maximum tumor length measured on CT images (mm) was not significantly different between the groups; however, the large PA lesion group showed an enrichment of larger tumor lengths (*p* = 0.097, MW-U). This result presumably indicated that CT detected many non-functional adenomas [14]. Although blood pressure values obtained under the use of antihypertensive agents were similar between the two groups, patients in the large PA lesion group were taking antihypertensives at significantly stronger intensities (2.00 [interquartile range: 1.00–2.50] vs. 1.33 [1.00–2.17], *p* = 0.012, MW-*U*). LR was significantly higher in the large PA lesion group (14.59 [4.79–31.86]) than in the small PA lesion group (5.24 [3.16–8.23], *p* < 0.001, MW-U). Preoperative serum K (the lowest value) was significantly higher in the small PA lesion group (mean ± S.D. = 3.61 ± 0.46 mEq/L) than in the large PA lesion group (2.86 ± 0.58 mEq/L, *p* < 0.001, unpaired *t*-test). Preoperatively, PAC was higher in the large PA lesion group than in the small PA lesion group, whereas PRA was not. Overall, the intensity of antihypertensives, LR, serum K, and PAC significantly differed preoperatively between the small and large PA lesion groups.

**Table 2:** Comparison of clinical data between small and large PA lesion groups (outcome cohort)

Variables	S-PAL group (*n* = 26)	*n*	L-PAL group (*n* = 87)	*n*	*p* values	Statistics
CT, tumor size (mm, threshold: 5 mm)	*10.0 (2.5–17.6)*	*26*	*15.0 (10.0–17.7)*	*87*	*0.097*	*MW-U*
Age (year)	*50.0 (43.0–63.0)*	*27*	*50.0 (41.0–61.0)*	*87*	*0.73*	*MW-U*
Sex (male vs. female)	*14 (51.9%) vs 13 (48.1%)*	*27*	*44 (50.6%) vs 43 (49.4%)*	*87*	*1.0*	*Fisher*
Side of adrenalectomy (left vs. right)	*17 (63.0%) vs 10 (37.0%)*	*27*	*46 (52.9%) vs 41 (47.1%)*	*87*	*0.39*	*Fisher*
Body mass index (kg/m^2^)	*24.4 (22.2–27.7)*	*27*	*23.1 (20.3–27.0)*	*87*	*0.29*	*MW-U*
eGFR (mL/min/1.73 m^2^)	*83.4 (74.3–96.5)*	*25*	*80.7 (68.1–97.5)*	*86*	*0.53*	*MW-U*
Systolic blood pressure (mmHg)	*138.8* ± *17.8*	*27*	*142.1* ± *18.3*	*85*	*0.41*	*uTT*
Diastolic blood pressure (mmHg)	*87.4* ± *12.7*	*27*	*86.7* ± *12.9*	*85*	*0.80*	*uTT*
**Intensity of antihypertensives**	**1.33 (1.00–2.17)**	**27**	**2.00 (1.00–2.50)**	**87**	**0.012**	**MW-U**
**Lateralized ratio (LR) in AVS**	**5.24 (3.16–8.23)**	**20**	**14.59 (4.79–31.86)**	**60**	**<0.001**	**MW-U**
**CYP11B2 positivity**	**3.26** ± **3.97**	**27**	**33.1** ± **12.8**	**87**	**<0.001**	***uTT***
**Serum K level (mEq/L)**	**3.61** ± **0.46**	**27**	**2.86** ± **0.58**	**86**	**<0.001**	**uTT**
**PAC (pg/mL)**	**164.0 (139.0–217.0)**	**27**	**320.0 (232.8–517.0)**	**86**	**<0.001**	**MW-U**
*PRA (ng/mL/hr)*	*0.200 (0.100–0.300)*	*27*	*0.200 (0.100–0.300)*	*86*	*0.51*	*MW-U*

When a tumor was not detected on CT and the size of tumor was shorter than 5 mm, the tumor size in statistical analyses was set at 2.5 mm

*BP* blood pressure, *eGFR* estimated glomerular filtration rate, *PAC* plasma aldosterone concentration, *PRA* plasma renin activity, *ARR* aldosterone-renin ratio = PAC/PRA, *AVS* adrenal venous sampling, *MW-U* the Mann–Whitney *U* test, the unpaired *t*-test: uTT, *S-PAL group* small PA lesion group, *L-PAL group* large PA lesion group

### Prediction model for the small PA lesion group

**Fig. 2:**
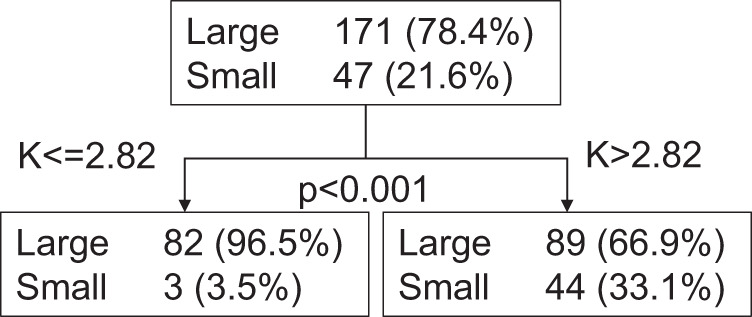
A classification tree predicting patients with small PA lesions for a serum K threshold of 2.82 mEq/L. The threshold for serum K was assessed by CHAID analysis. “Large” and “Small” indicate patient groups with large and small PA lesions, respectively. Numbers without and with parentheses within each node indicate the number and percentage of patients, respectively. The upper node was separated from the lower nodes based on the serum K value (≤2.82 mEq/L vs. >2.82 mEq/L). The *p* value was calculated by Fisher’s exact test

To investigate whether a preoperative prediction model could be generated that differentiates between the small and large PA lesion groups, we used preoperative factors with significant differences in univariable analyses for multivariable logistic regression analysis (i.e., intensity of antihypertensives, serum K levels, and PAC). LR was removed from the analysis due to the lack of data in many patients. The results showed that serum K and PAC were independent predictive factors (*n* = 112 [two cases were not included due to missing data], test cohort in Table 3). Since the number of cases in the test cohort was limited, to confirm the model, we utilized cases without postoperative biochemical outcomes (*n* = 105, validation cohort in Table 3) for which an analysis was possible without outcome data. Although preoperative PAC was not a predictive factor for PA lesion sizes in this cohort, serum K level was. To identify a predictive threshold value for preoperative serum K that differentiated small and large PA lesions, we performed CHAID analysis using data from patients with available preoperative serum K and pathological data (*n* = 218, Supplementary Table 1). In the cohort with serum K levels less than or equal to 2.82 mEq/L, only three out of 85 patients (3.5%) had small PA lesions (<5 mm), whereas in the cohort with serum K levels higher than 2.82 mEq/L, 44 out of 133 patients (33.1%) had small lesions (*p* < 0.001, Fig. 2). Overall, this model indicated that cases with a preoperative serum K level <2.82 mEq/L had a markedly higher chance of having large PA lesions, and, thus, potentially unilateral lesions. These results will be beneficial for the clinical practice of PA because cases with low serum K levels and apparent adrenal masses on CT could be subjected to adrenalectomy even if the AVS test is unavailable.

**Table 3:** Logistic regression analyses to predict cases in the small PA lesion group

		Crude			Adjusted		
Variables		Odds ratio	95% confidence interval	*p* value	Odds ratio	95% confidence interval	*p* value
Test cohort	*Intensity of antihypertensives*	*0.645*	*(0.389–1.071)*	*0.09*			
(Small PA lesion group/total = 27/112)	**Serum K level (mEq/L)**	**5.877**	**(1.928–17.911)**	**0.002**	**6.277**	**(2.081–18.934)**	**0.001**
	**PAC**	**0.994**	**(0.989–1.000)**	**0.037**	**0.994**	**(0.989–1.000)**	**0.037**
Validation cohort	*Intensity of antihypertensives*	*0.921*	*(0.573–1.479)*	*0.73*			
(Small PA lesion group/total = 20/105)	**Serum K level (mEq/L)**	**5.072**	**(1.493–17.231)**	**0.009**	**6.601**	**(2.214–19.681)**	**<0.001**
	*PAC*	*0.998*	*(0.994–1.003)*	*0.41*			

*CT* computed tomography, *PAC* plasma aldosterone concentration (pg/mL), *ARR* aldosterone-renin ratio

## Discussion

We herein retrospectively investigated the relationships between preoperative clinical data, pathological findings, and postoperative biochemical/clinical outcomes using a large sample from JRAS: (i) we generated the largest catalog (https://humandbs.biosciencedbc.jp/en/hum0185-v1-st1) of heterogeneous PA pathology that allowed a simple classification of small and large PA lesions at a threshold of 5 mm, which correlated with postoperative biochemical outcomes but not clinical outcomes, and (ii) we demonstrated that cases with preoperative serum K levels <2.82 mEq/L had a markedly higher chance of having potentially unilateral, large PA lesions. These results will be beneficial for PA in clinical settings and the pathological diagnosis of PA lesions, particularly for cases with lower serum K levels and apparent tumor(s) on CT, because these tumor(s) must be APA rather than incidentaloma and may allow the patient to avoid invasive AVS.

Following the development of CYP11B2 immunohistochemistry, we proposed new terms to describe small PA lesions: APCCs [5] and pAATLs [6] (refer to the Introduction section for the definition of these terms). These terms not only delineate the morphology of PA lesions but also speculate upon their function/pathophysiology. The term APCC appears to be widely accepted, whereas pAATL has only been used by a small number of research groups [15, 16], possibly because the transition from APCC to APA has not yet been demonstrated. Another group defined “aldosterone-producing micronodules” as “CYP11B2-positive cortical micronodules located in the CYP11B2-negative ZG” [11, 17]. These lesions appear to be similar to pAATLs and small APA lesions [7, 10]. Thus, we and others have utilized multiple terms to describe different types of PA lesions, and none of them may be sufficiently complete or comprehensive. We noted that when utilizing our definition, it was frequently difficult to define the diagnosis of PA lesions, as shown in Supplementary Table 1 (diagnosis with “/” in the column “initial pathological diagnosis”). As shown in the present study, simple grouping by the size of the largest PA lesion for a threshold value of 5 mm may be more useful for both clinical practice and pathological diagnoses because it is associated with postoperative biochemical outcomes.

The sizes of PA lesions were associated with postoperative biochemical outcomes but not clinical outcomes. These outcomes were recently defined by the Primary Aldosteronism Surgical Outcome (PASO) study using the Delphi method [13]. The biochemical outcome of adrenalectomy is principally associated with AVS results, which were also strongly associated with the size of PA lesions in the present study (the values of LR from AVS of the small and large PA lesion groups were 5.24 and 14.59, respectively [*p* < 0.001]). Clinical outcomes appear to be associated with the normalization of the aldosterone secretion status as well as systemic vascular damage due to PA and other disorders (e.g., lifestyle-related diseases). The large PA lesion group in the present study had hypertension for a longer period, and prolonged hyperaldosteronism in these patients appeared to have diminished the effect of lesion removal on clinical outcome.

Several models for predicting unilateral or bilateral PA, the diagnosis of which was based on AVS but not on pathology, have been previously proposed [18–21]. All of these models include serum K as one of the predictors at a threshold of 3.5 mEq/L, presumably because this value is often the lower limit of the normal range. As shown above, we demonstrated that the best threshold value for preoperative serum K was 2.82 mEq/L using CHAID analysis, and only 3.5% of patients had small PA lesions when the preoperative serum K level was <2.82 (Fig. 2). At the threshold value of 3.5 mEq/L, in the cohort with serum K levels at or <3.5 mEq/L, 19 out of 162 patients (11.7%) had small PA lesions (<5 mm), whereas in the cohort with serum K levels more than 3.5 mEq/L, 50% of 56 patients had small lesions (Supplementary Fig. 3), and the classification tree was very different from that in Fig. 2. Therefore, these results will be beneficial for PA in clinical settings because patients with low serum K levels and apparent adrenal masses on CT could be subjected to adrenalectomy even if the AVS test is unavailable.

As a follow-up to this retrospective study, we recently initiated a prospective study to collect additional data, including CT images analyzed by four independent radiologists, a pathological analysis of multiple parts of a resected adrenal gland, and the most reliable AVS method, superselective AVS [ssAVS] [6, 22, 23]. We designed a new study based on the limitations in the present study, which included (i) its retrospective design with inconsistent data availability, including biochemical/clinical outcomes, (ii) the investigation of cases that underwent unilateral adrenalectomy but excluding bilateral PA, (iii) CT findings reported by different investigators/clinicians across multiple institutions, and (iv) pathological analyses performed with only one or two sections from each case. In the new study with ssAVS, blood samples are obtained not only from the adrenal central veins but also from the tributary veins of the adrenal central veins using a microcatheter. Therefore, the ssAVS method may distinguish multiple regions within an adrenal. For example, ssAVS for Case 149 (Fig. 1C, D) may show high PAC in the tributary vein from the APA and low (or suppressed) PAC in those from adjacent adrenal regions. This region specificity will allow us to perform partial adrenalectomy on cases with bilateral lesions and elucidate the relationship between lesion sizes and biochemical outcomes. Thus, the new prospective study will confirm the outcomes of the present retrospective study, even for bilateral PA cases.

In summary, we generated the largest catalog of heterogeneous PA pathology and demonstrated that pathological PA lesions or larger than or equal to 5 mm in size correlated with postoperative biochemical outcomes and that PA patients with serum K levels at or <2.8 mEq/L had a significantly higher chance of having APA than incidentaloma. Based on these results, we concluded that PA cases with serum K levels less than or equal to 2.82 mEq/L and with an apparent single adenoma on CT may undergo unilateral adrenalectomy even in the absence of AVS because the adenoma must be a large PA lesion (i.e., apparent APA).

## Supplementary information

Supplementary Figure 1

Supplementary Figure 2

Supplementary Figure 3

Supplementary Materials and Methods

Supplementary Table 1

Supplementary Table 2

Supplementary Table 3

Supplementary Data 2
