# Supplementary material for: Diverse pathological lesions of primary aldosteronism and their clinical significance
Source: Hypertens Res. 2021 Jan 12;44(5):498–507. doi: 10.1038/s41440-020-00579-w (PMC8099725; doi:10.1038/s41440-020-00579-w)

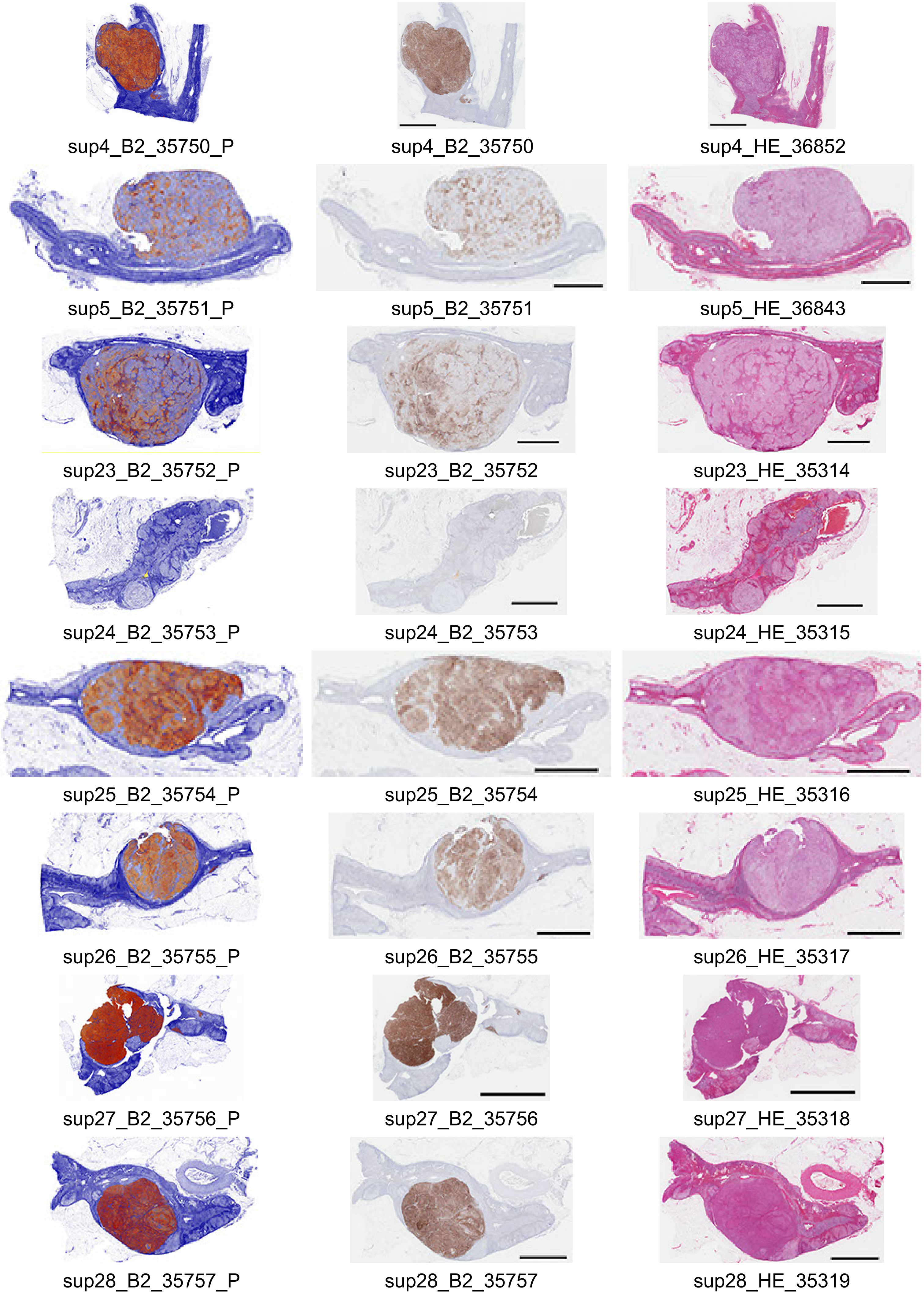
sup4_B2_35750_P
sup4_B2_35750
sup4_HE_36852
sup5_B2_35751_P
sup5_B2_35751
sup5_HE_36843
sup23_B2_35752_P
sup23_B2_35752
sup23_HE_35314
sup24_B2_35753_P
sup24_B2_35753
sup24_HE_35315
sup25_B2_35754_P
sup25_B2_35754
sup25_HE_35316
sup26_B2_35755_P
sup26_B2_35755
sup26_HE_35317
sup27_B2_35756_P
sup27_B2_35756
sup27_HE_35318
sup28_B2_35757_P
sup28_B2_35757
sup28_HE_35319

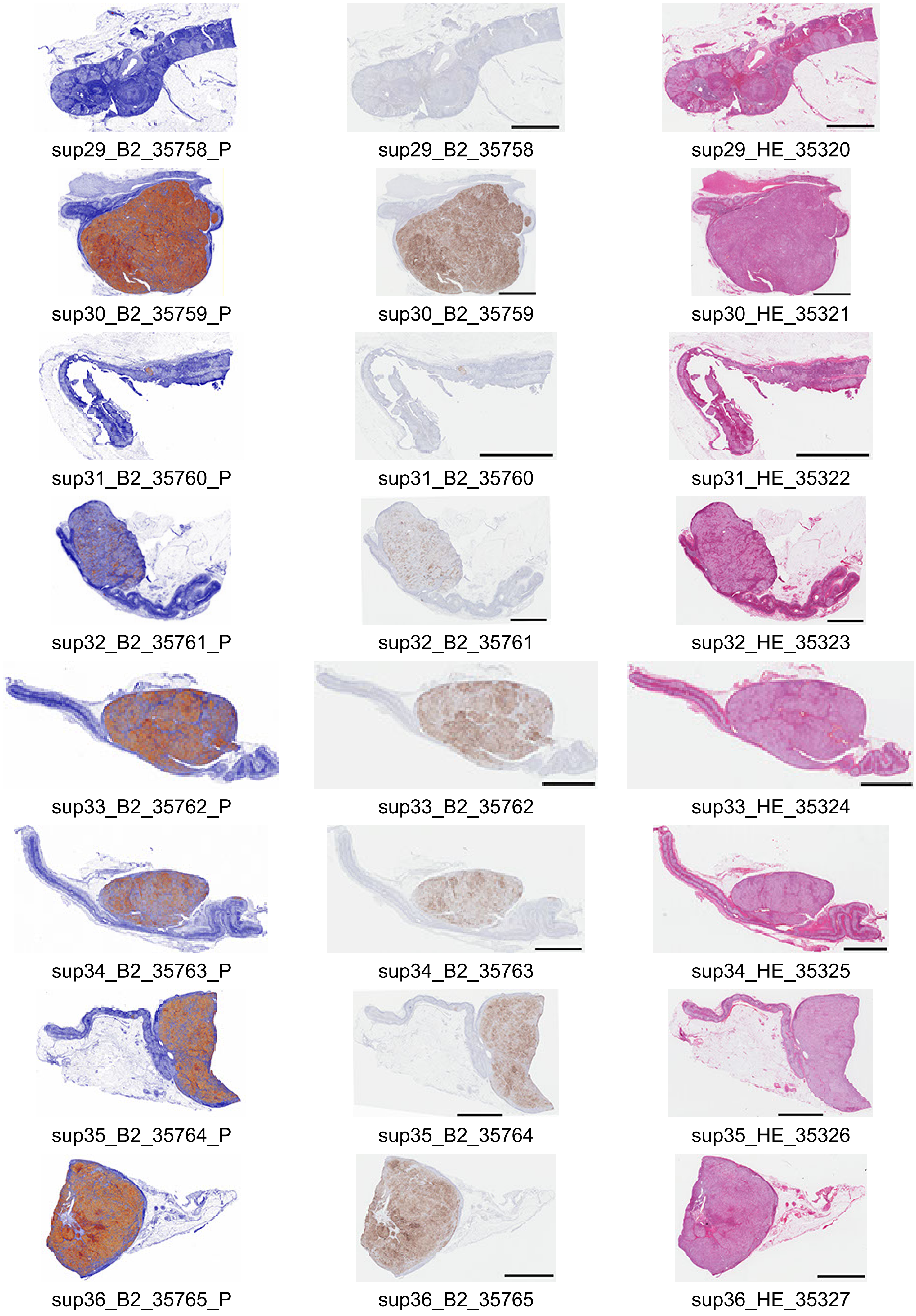
sup29_B2_35758_P
sup29_B2_35758
sup29_HE_35320
sup30_B2_35759_P
sup30_B2_35759
sup30_HE_35321
sup31_B2_35760_P
sup31_B2_35760
sup31_HE_35322
sup32_B2_35761_P
sup32_B2_35761
sup32_HE_35323
sup33_B2_35762_P
sup33_B2_35762
sup33_HE_35324
sup34_B2_35763_P
sup34_B2_35763
sup34_HE_35325
sup35_B2_35764_P
sup35_B2_35764
sup35_HE_35326
sup36_B2_35765_P
sup36_B2_35765
sup36_HE_35327

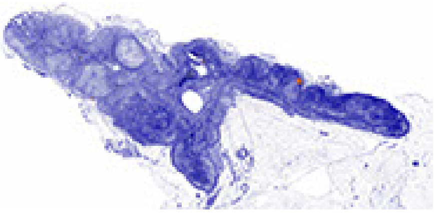
sup37_B2_35766_P
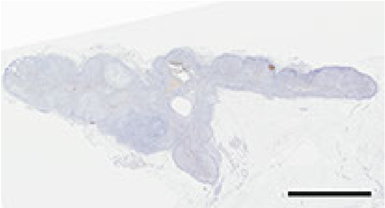
sup37_B2_35766
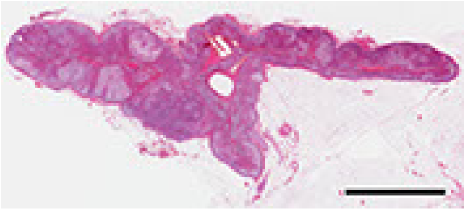
sup37_HE_35449

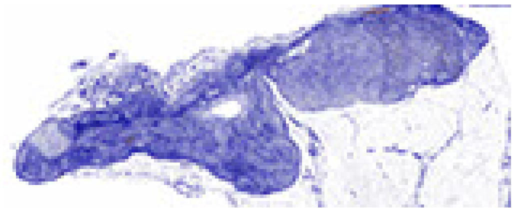
sup38_B2_35767_P
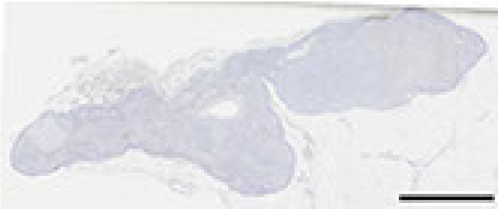
sup38_B2_35767
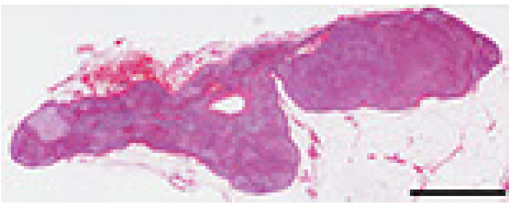
sup38_HE_35329

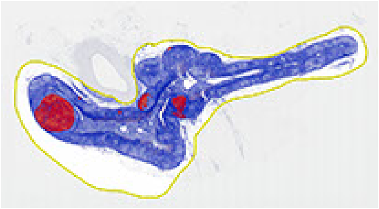
sup39_B2_35768_P
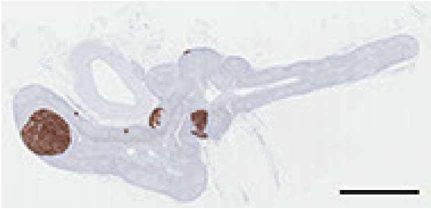
sup39_B2_35768
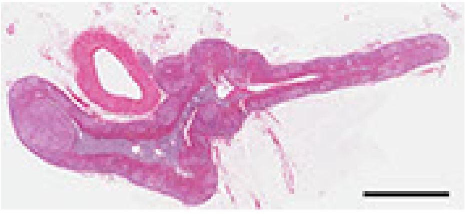
sup39_HE_35450

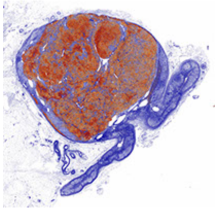
sup40_B2_35769_P
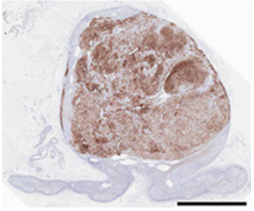
sup40_B2_35769
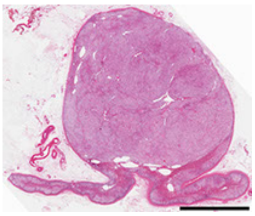
sup40_HE_35451

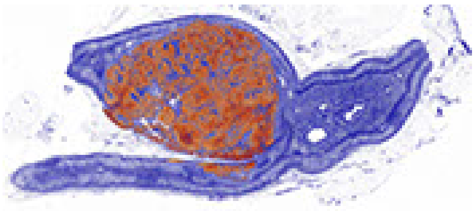
sup41_B2_35730_P
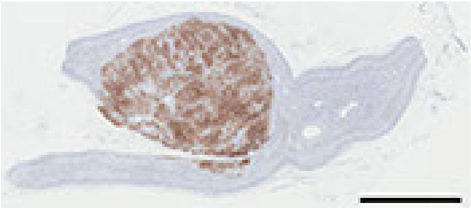
sup41_B2_35730
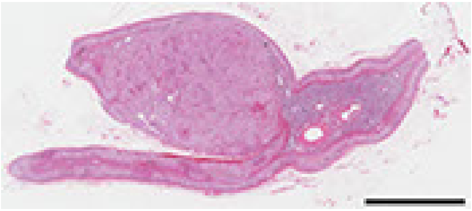
sup41_HE_35332

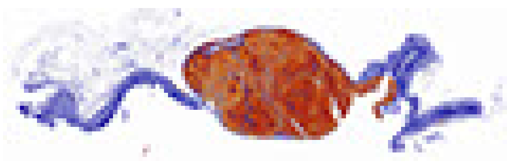
sup42_B2_35731_P
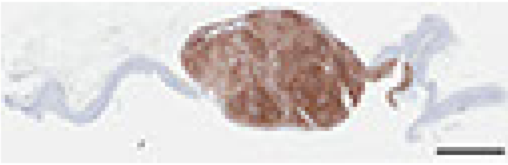
sup42_B2_35731
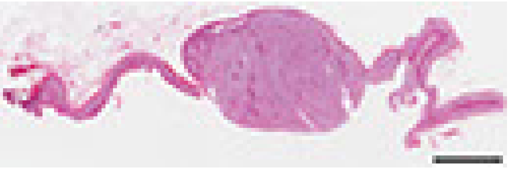
sup42_HE_35333

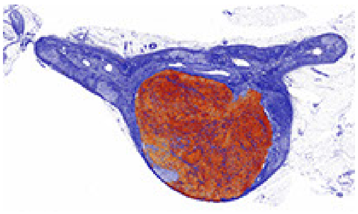
sup43_B2_35732_P
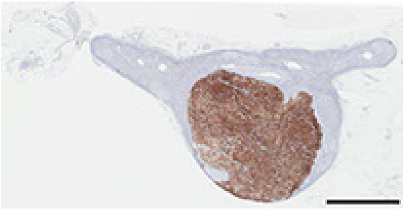
sup43_B2_35732
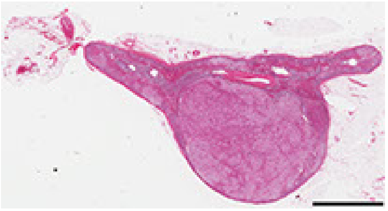
sup43_HE_35334

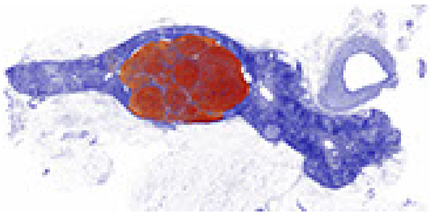
sup44_B2_35733_P
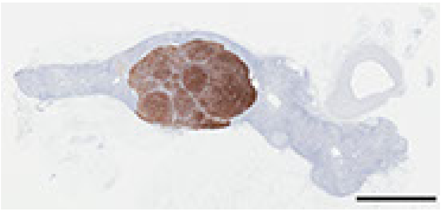
sup44_B2_35733
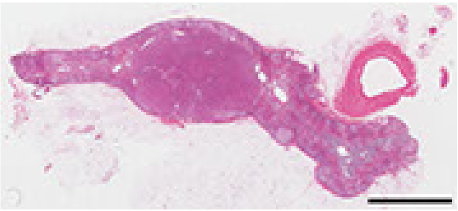
sup44_HE_35335

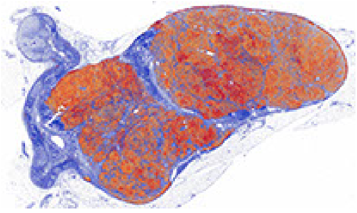
sup45_B2_35777_P

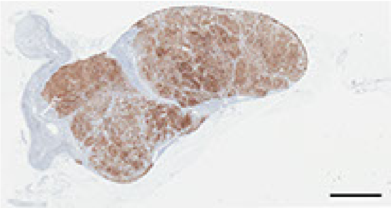
sup45_B2_35777

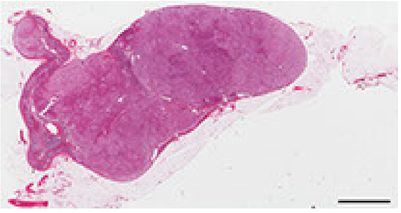
sup45_HE_35336

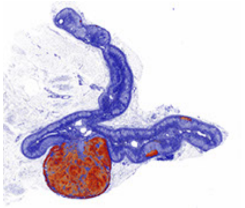
sup46_B2_35776_P

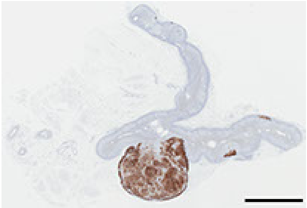
sup46_B2_35776

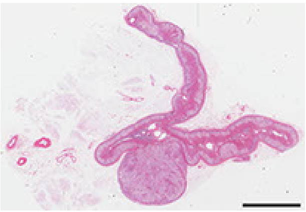
sup46_HE_35337

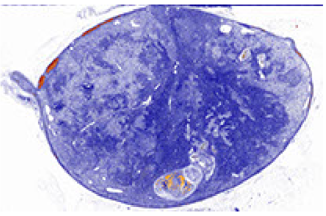
sup47_B2_35775_P

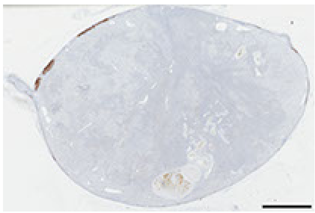
sup47_B2_35775

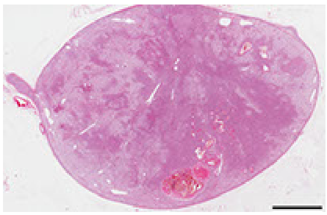
sup47_HE_35338

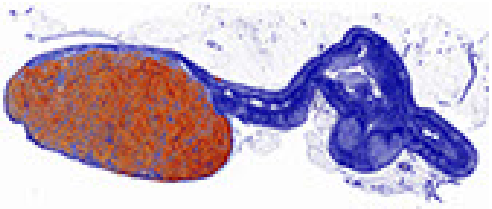
sup48_B2_35774_P

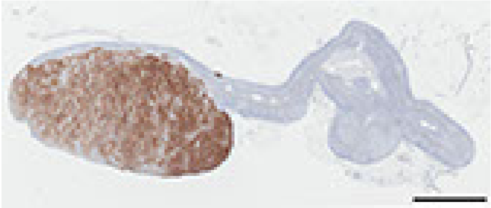
sup48_B2_35774

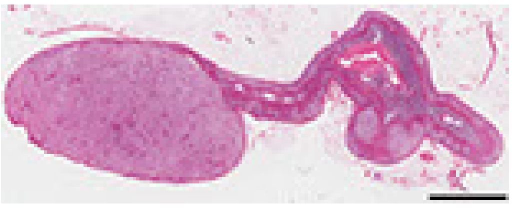
sup48_HE_35339

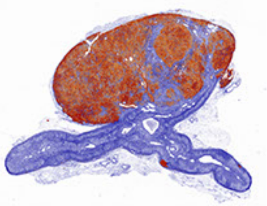
sup49_B2_35773_P

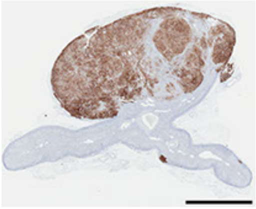
sup49_B2_35773

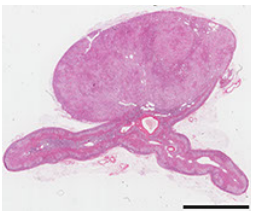
sup49_HE_35340

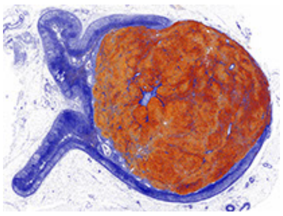
sup50_B2_35772_P

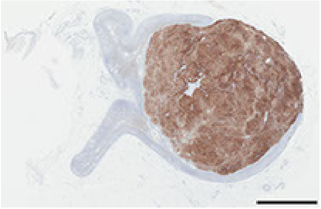
sup50_B2_35772

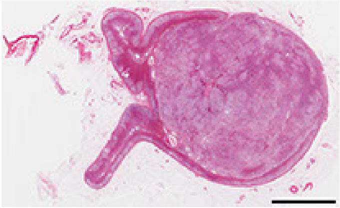
sup50_HE_35341

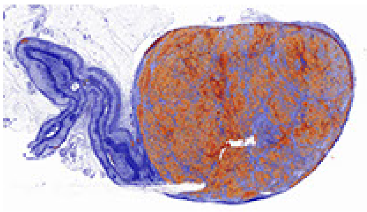
sup51_B2_35771_P

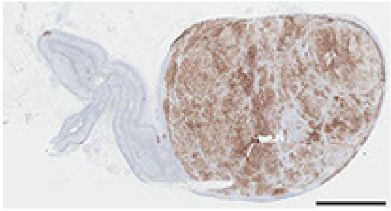
sup51_B2_35771

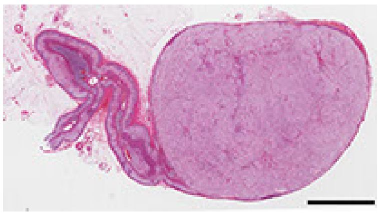
sup51_HE_35342

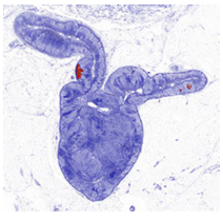
sup52_B2_35770_P

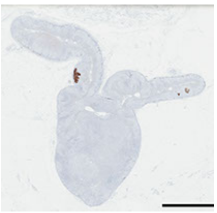
sup52_B2_35770

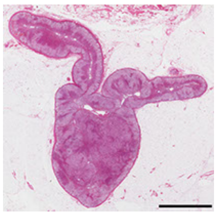
sup52_HE_35343

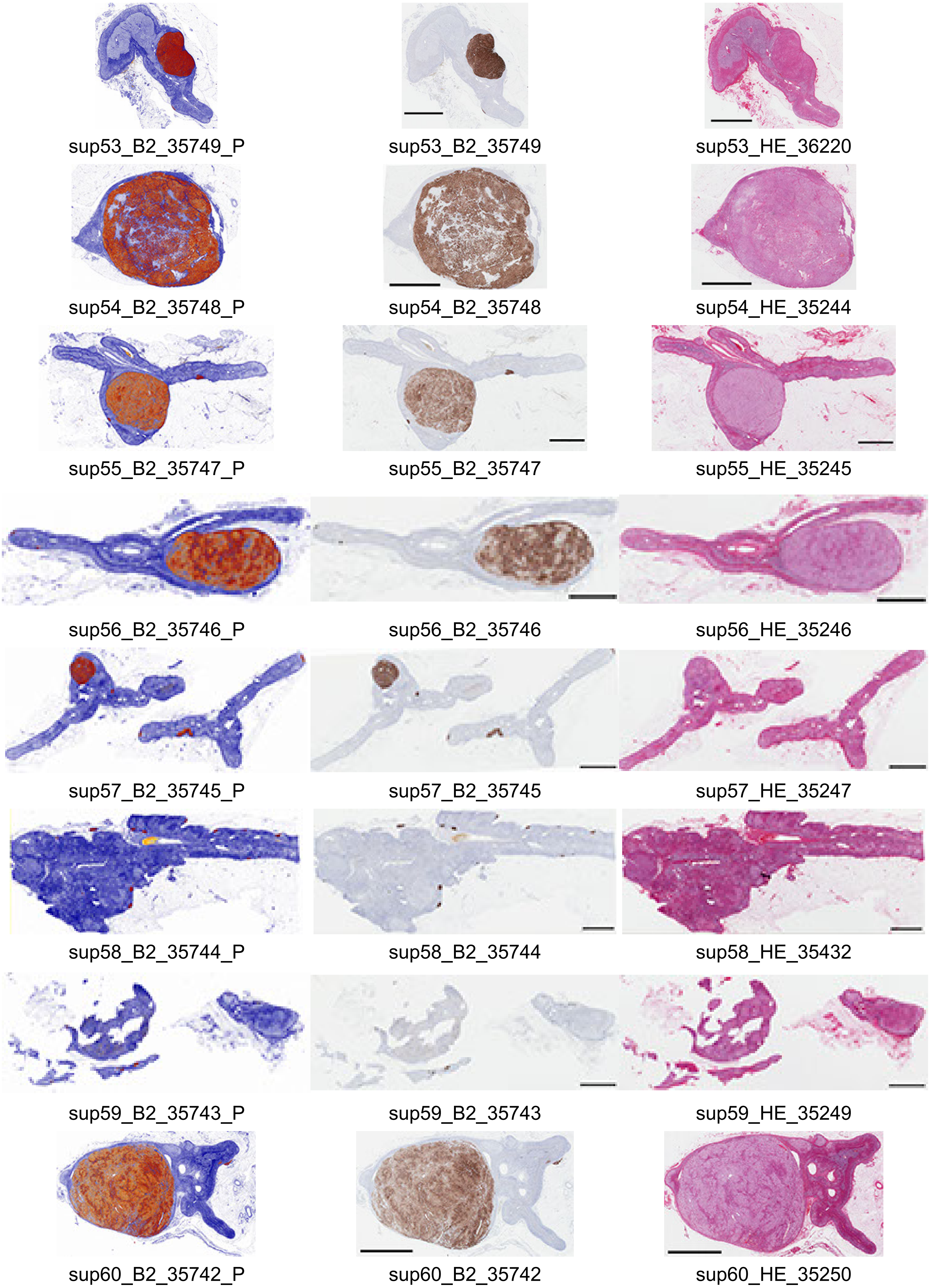
sup53_B2_35749_P
sup53_B2_35749
sup53_HE_36220
sup54_B2_35748_P
sup54_B2_35748
sup54_HE_35244
sup55_B2_35747_P
sup55_B2_35747
sup55_HE_35245
sup56_B2_35746_P
sup56_B2_35746
sup56_HE_35246
sup57_B2_35745_P
sup57_B2_35745
sup57_HE_35247
sup58_B2_35744_P
sup58_B2_35744
sup58_HE_35432
sup59_B2_35743_P
sup59_B2_35743
sup59_HE_35249
sup60_B2_35742_P
sup60_B2_35742
sup60_HE_35250

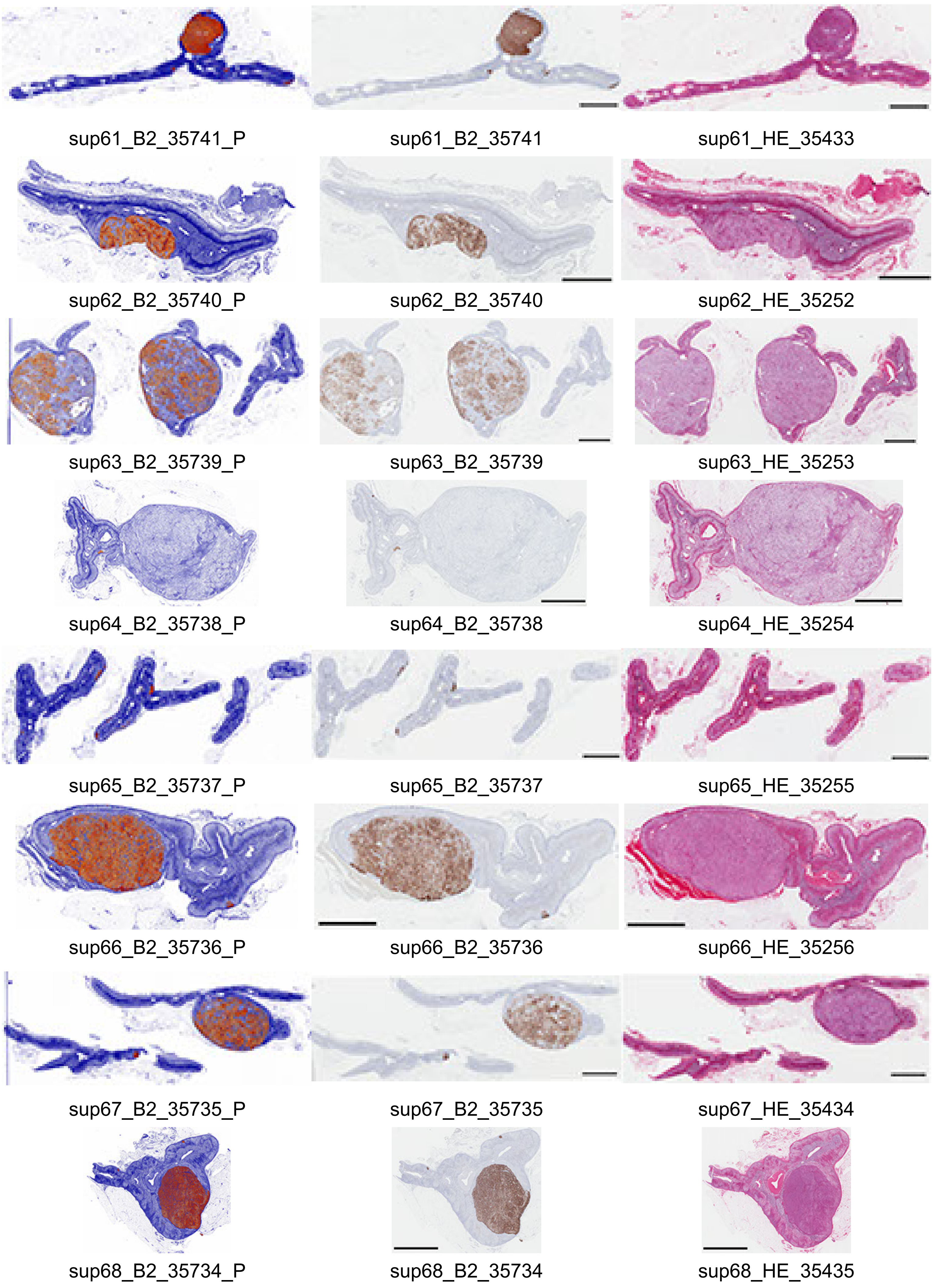
sup61_B2_35741_P
sup61_B2_35741
sup61_HE_35433
sup62_B2_35740_P
sup62_B2_35740
sup62_HE_35252
sup63_B2_35739_P
sup63_B2_35739
sup63_HE_35253
sup64_B2_35738_P
sup64_B2_35738
sup64_HE_35254
sup65_B2_35737_P
sup65_B2_35737
sup65_HE_35255
sup66_B2_35736_P
sup66_B2_35736
sup66_HE_35256
sup67_B2_35735_P
sup67_B2_35735
sup67_HE_35434
sup68_B2_35734_P
sup68_B2_35734
sup68_HE_35435

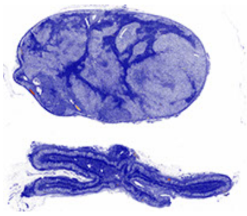
sup69_B2_35778_P
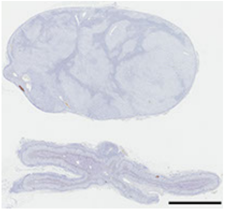
sup69_B2_35778
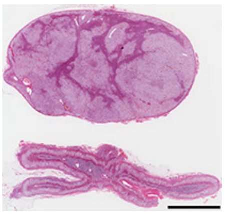
sup69_HE_35436

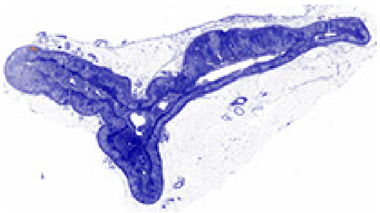
sup70_B2_35779_P
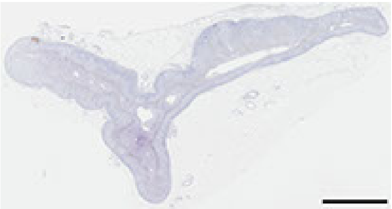
sup70_B2_35779
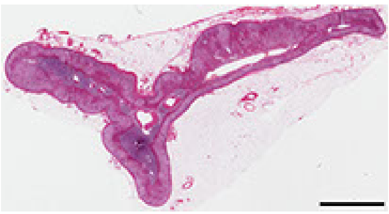
sup70_HE_35260

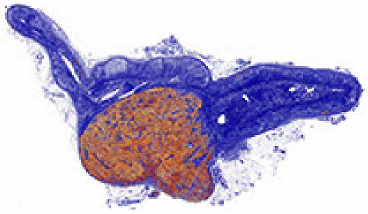
sup71_B2_35780_P
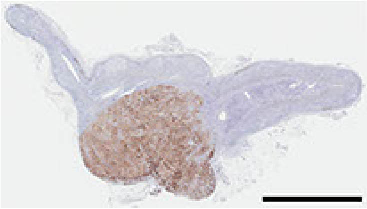
sup71_B2_35780
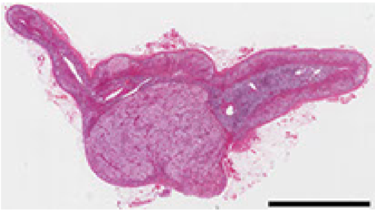
sup71_HE_35261

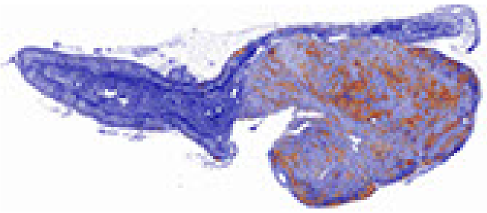
sup72_B2_35781_P
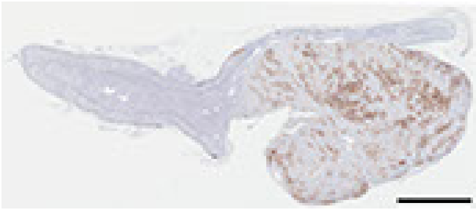
sup72_B2_35781
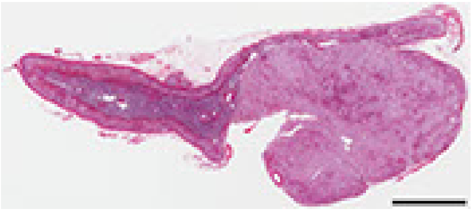
sup72_HE_35437

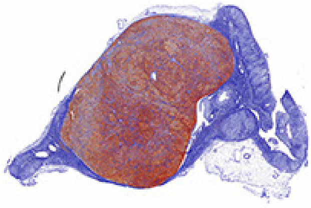
sup73_B2_35782_P
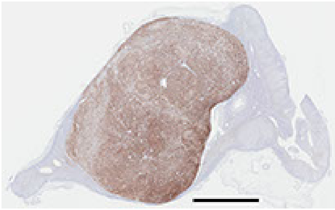
sup73_B2_35782
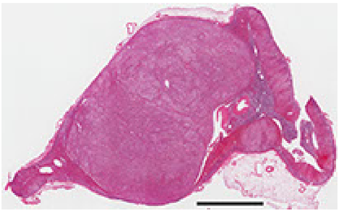
sup73_HE_35438

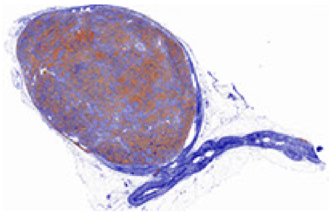
sup74_B2_35783_P
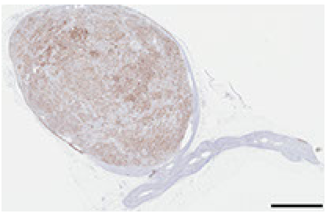
sup74_B2_35783
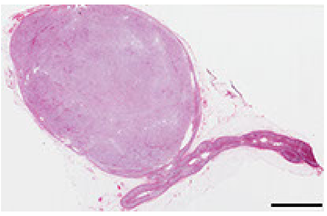
sup74_HE_35264

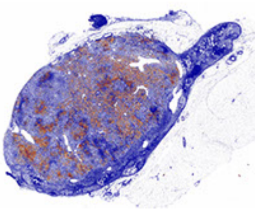
sup75_B2_35784_P
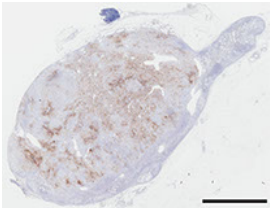
sup75_B2_35784
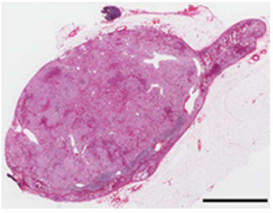
sup75_HE_35265

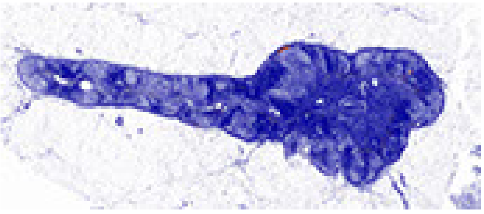
sup76_B2_35785_P
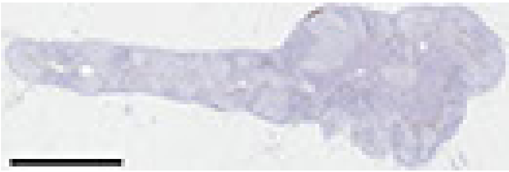
sup76_B2_35785
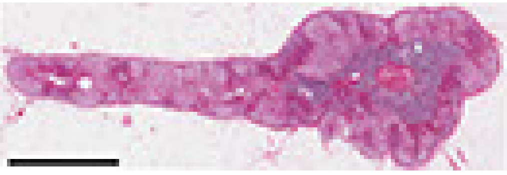
sup76_HE_35439

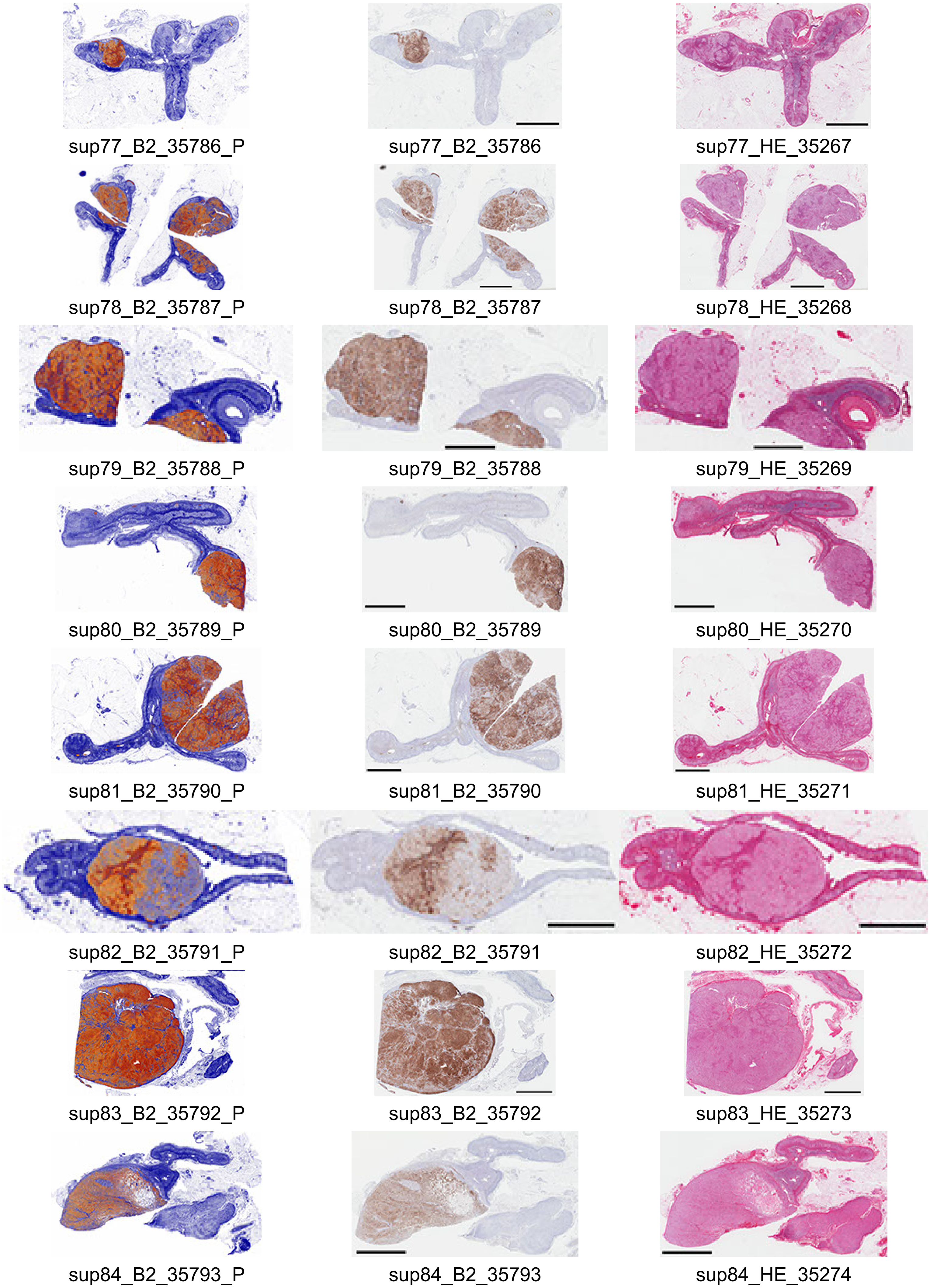
sup77_B2_35786_P
sup77_B2_35786
sup77_HE_35267
sup78_B2_35787_P
sup78_B2_35787
sup78_HE_35268
sup79_B2_35788_P
sup79_B2_35788
sup79_HE_35269
sup80_B2_35789_P
sup80_B2_35789
sup80_HE_35270
sup81_B2_35790_P
sup81_B2_35790
sup81_HE_35271
sup82_B2_35791_P
sup82_B2_35791
sup82_HE_35272
sup83_B2_35792_P
sup83_B2_35792
sup83_HE_35273
sup84_B2_35793_P
sup84_B2_35793
sup84_HE_35274

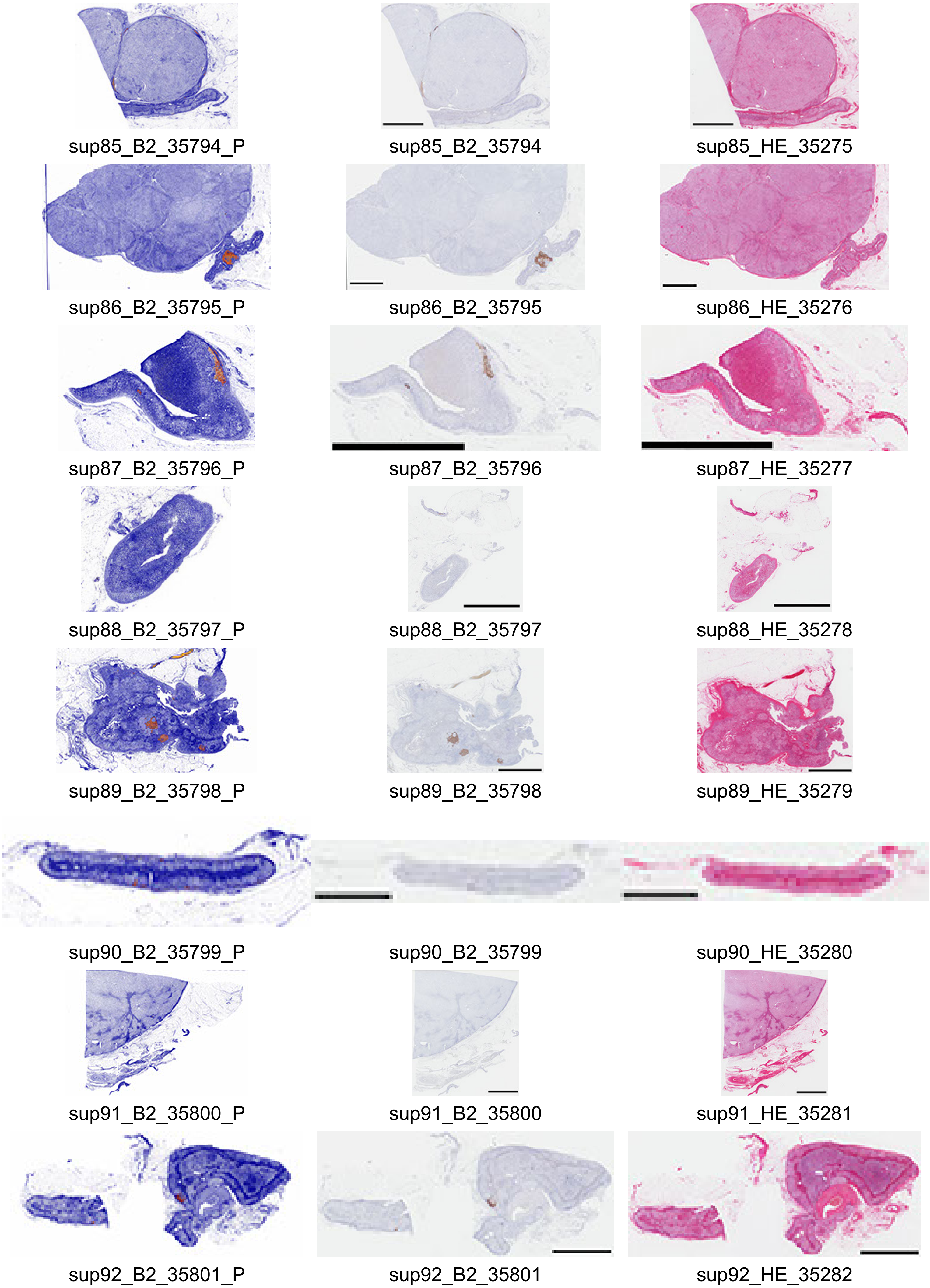
sup85_B2_35794_P
sup85_B2_35794
sup85_HE_35275
sup86_B2_35795_P
sup86_B2_35795
sup86_HE_35276
sup87_B2_35796_P
sup87_B2_35796
sup87_HE_35277
sup88_B2_35797_P
sup88_B2_35797
sup88_HE_35278
sup89_B2_35798_P
sup89_B2_35798
sup89_HE_35279
sup90_B2_35799_P
sup90_B2_35799
sup90_HE_35280
sup91_B2_35800_P
sup91_B2_35800
sup91_HE_35281
sup92_B2_35801_P
sup92_B2_35801
sup92_HE_35282

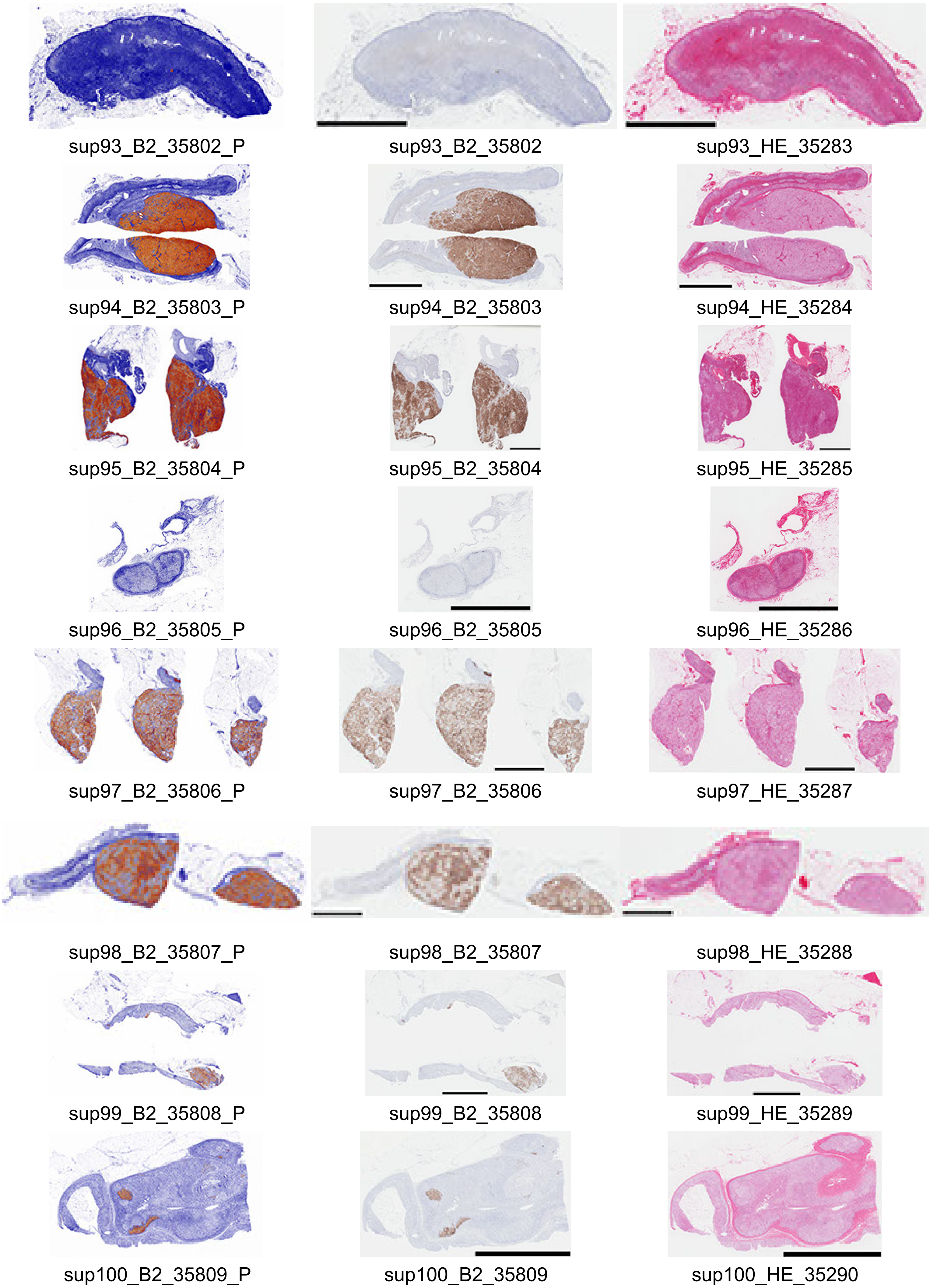
sup93_B2_35802_P
sup93_B2_35802
sup93_HE_35283
sup94_B2_35803_P
sup94_B2_35803
sup94_HE_35284
sup95_B2_35804_P
sup95_B2_35804
sup95_HE_35285
sup96_B2_35805_P
sup96_B2_35805
sup96_HE_35286
sup97_B2_35806_P
sup97_B2_35806
sup97_HE_35287
sup98_B2_35807_P
sup98_B2_35807
sup98_HE_35288
sup99_B2_35808_P
sup99_B2_35808
sup99_HE_35289
sup100_B2_35809_P
sup100_B2_35809
sup100_HE_35290

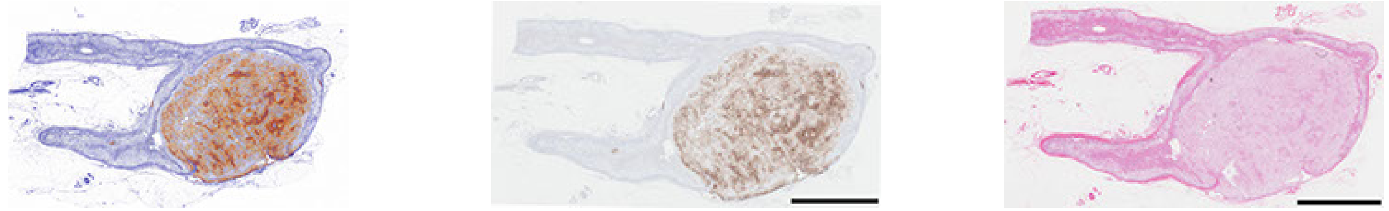

sup101_B2_35810_P sup101_B2_35810 sup101_HE_35301

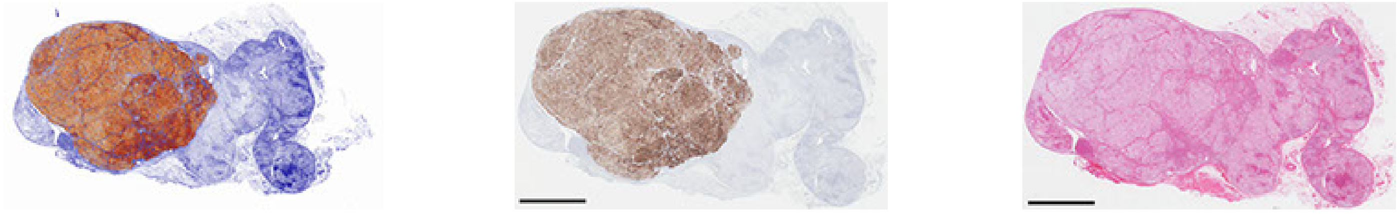

sup102_B2_35811_P sup102_B2_35811 sup102_HE_35302

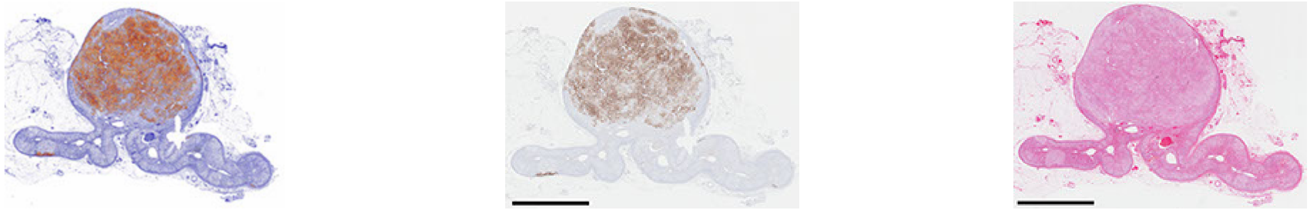

sup103_B2_35812_P sup103_B2_35812 sup103_HE_35303

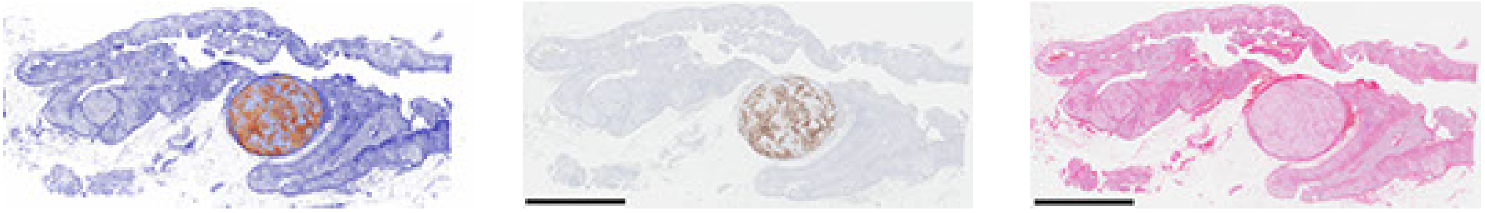

sup104_B2_35813_P sup104_B2_35813 sup104_HE_35441

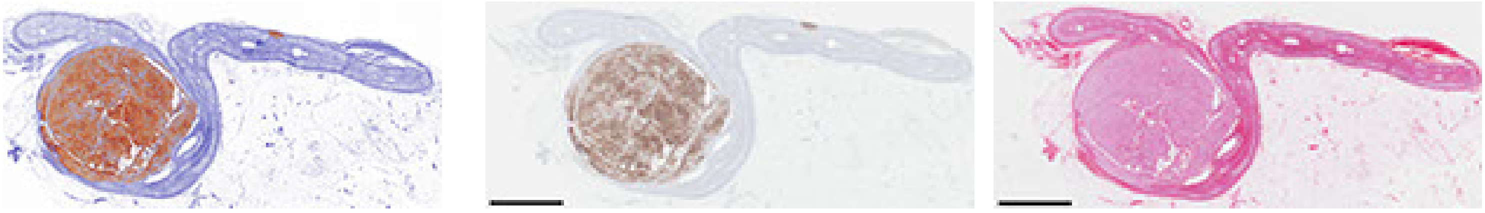

sup105_B2_35814_P sup105_B2_35814 sup105_HE_35305

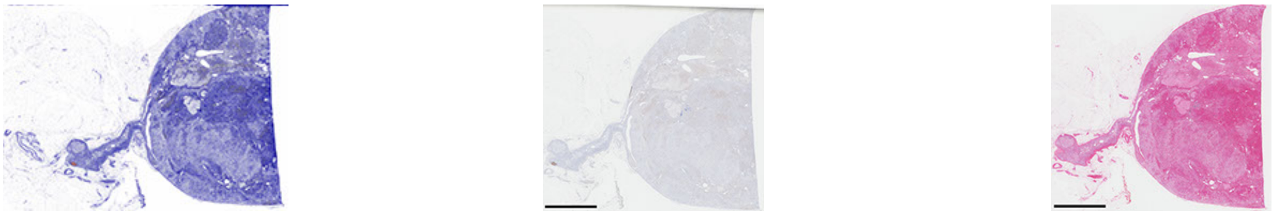

sup106_B2_35815_P sup106_B2_35815 sup106_HE_35442

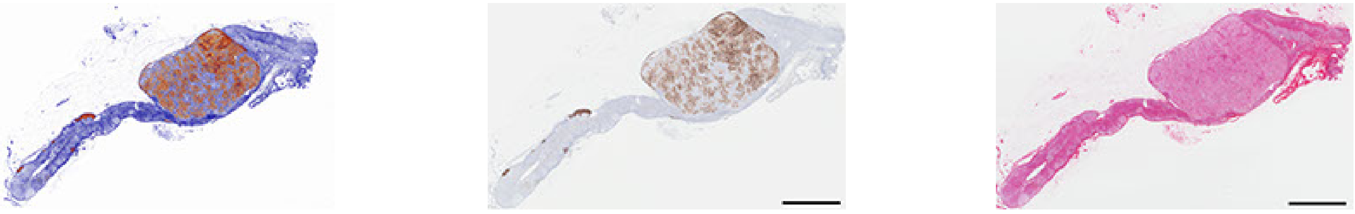

sup107_B2_35816_P sup107_B2_35816 sup107_HE_35443

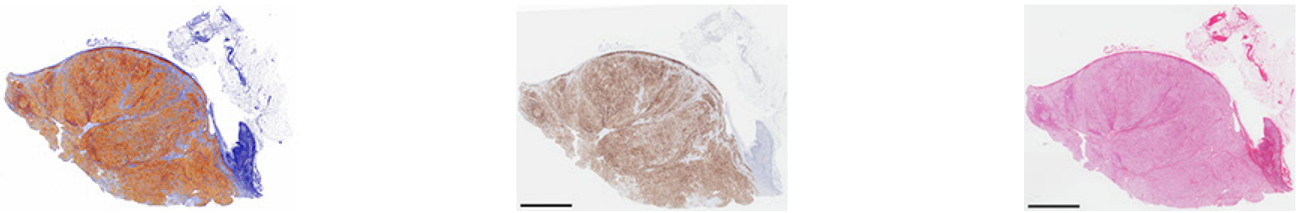

sup108_B2_35817_P sup108_B2_35817 sup108_HE_35444

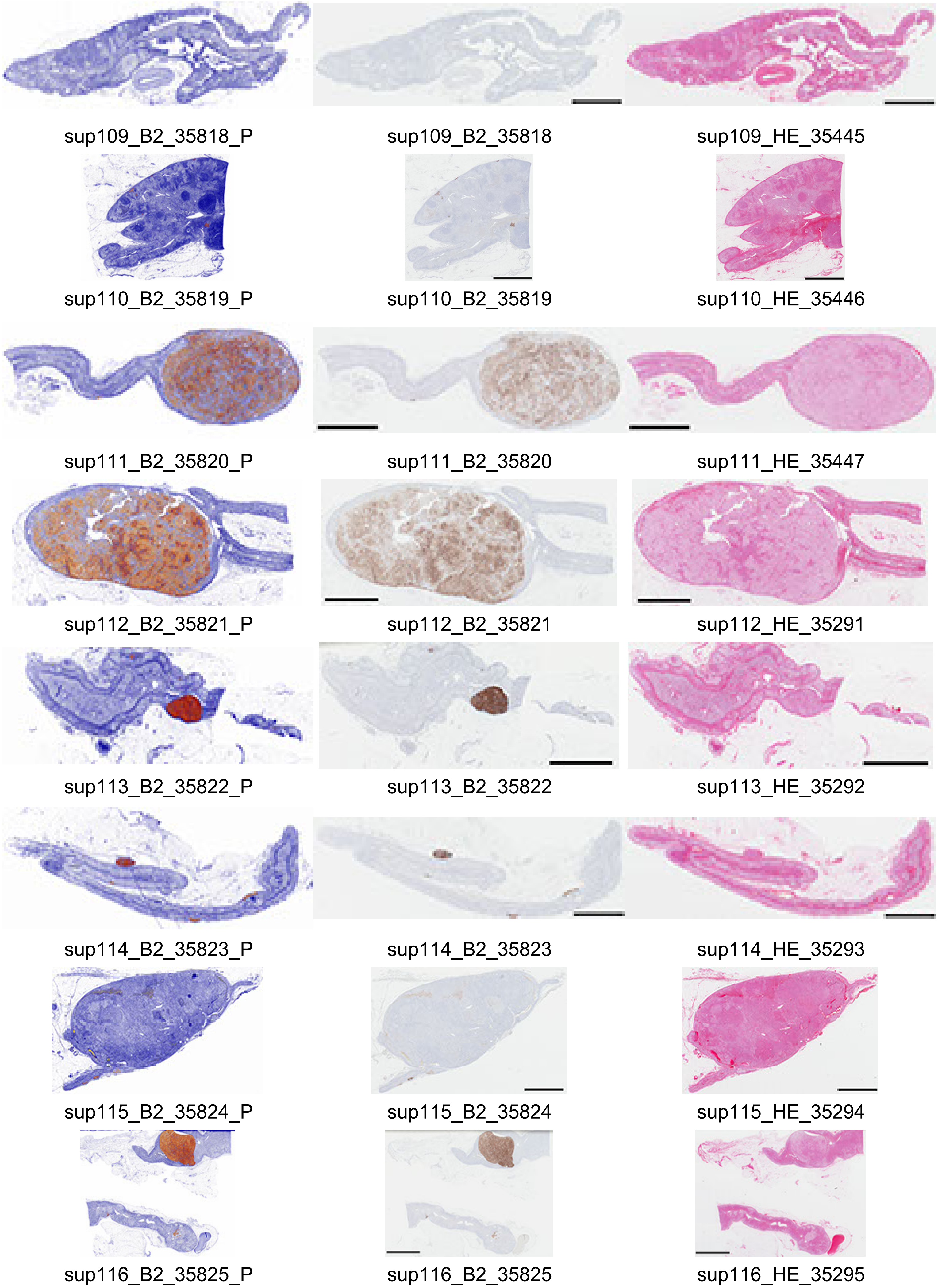
sup109_B2_35818_P
sup109_B2_35818
sup109_HE_35445
sup110_B2_35819_P
sup110_B2_35819
sup110_HE_35446
sup111_B2_35820_P
sup111_B2_35820
sup111_HE_35447
sup112_B2_35821_P
sup112_B2_35821
sup112_HE_35291
sup113_B2_35822_P
sup113_B2_35822
sup113_HE_35292
sup114_B2_35823_P
sup114_B2_35823
sup114_HE_35293
sup115_B2_35824_P
sup115_B2_35824
sup115_HE_35294
sup116_B2_35825_P
sup116_B2_35825
sup116_HE_35295

Supplementary Figure 1

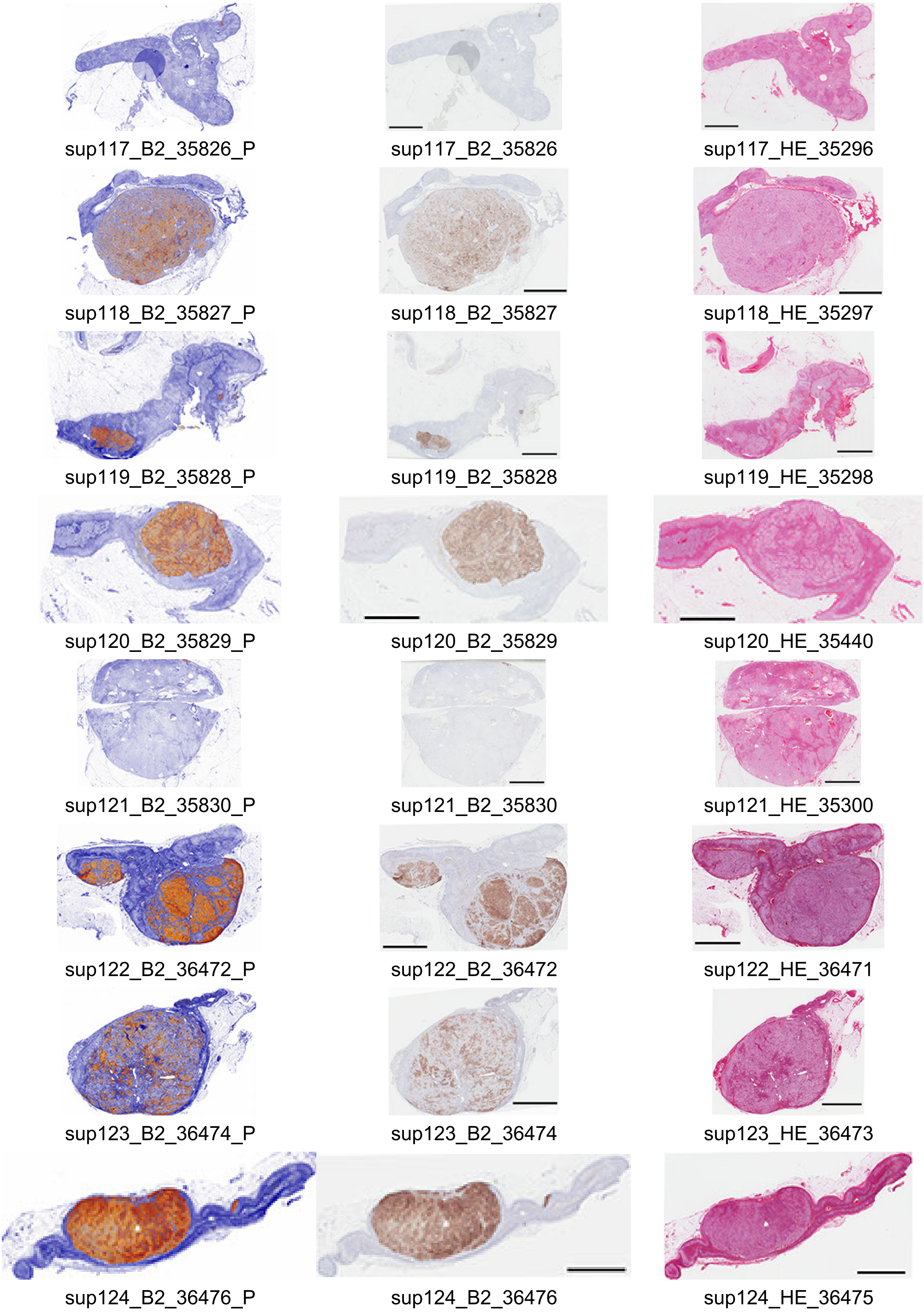

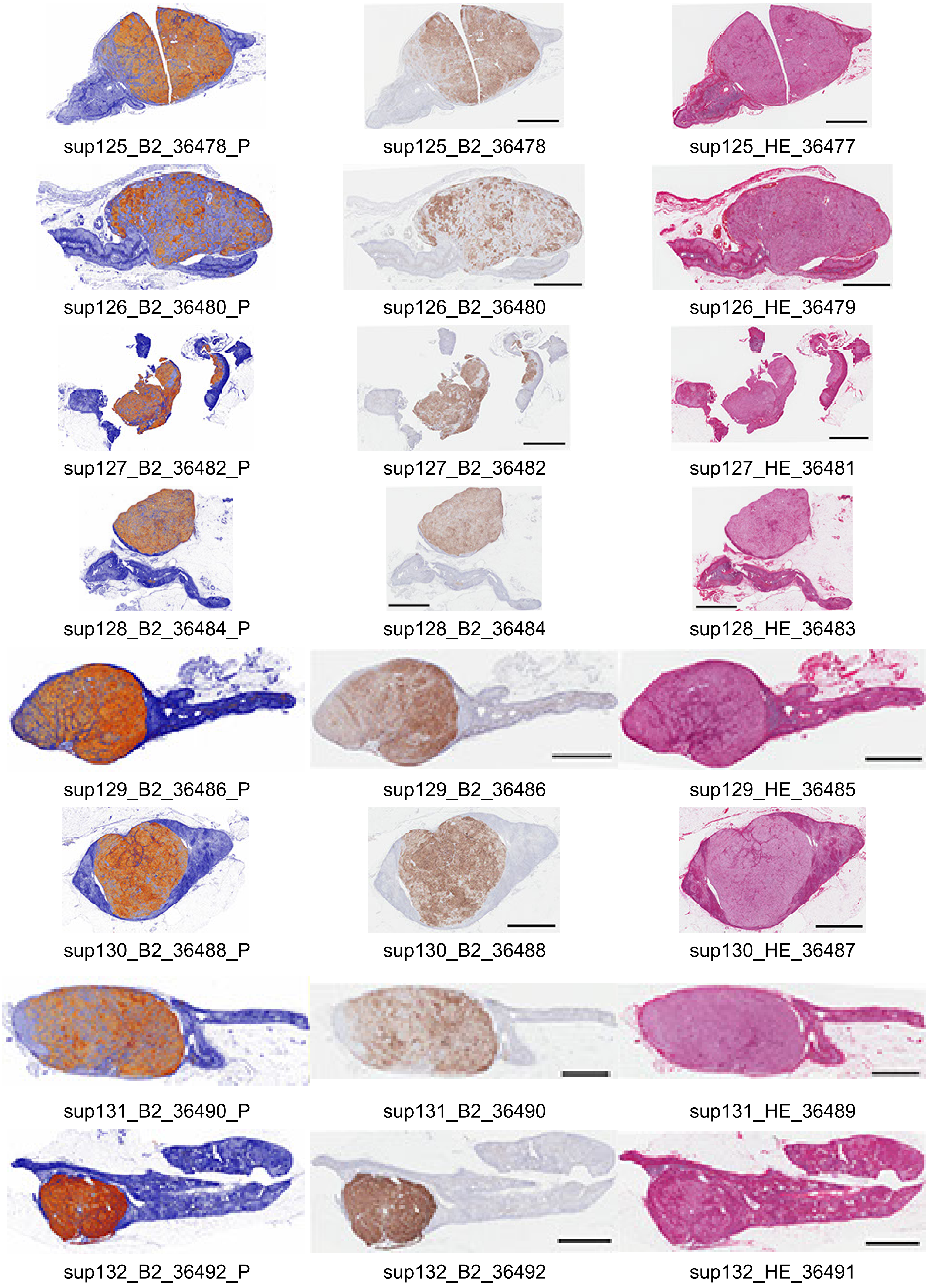
sup125_B2_36478_P
sup125_B2_36478
sup125_HE_36477
sup126_B2_36480_P
sup126_B2_36480
sup126_HE_36479
sup127_B2_36482_P
sup127_B2_36482
sup127_HE_36481
sup128_B2_36484_P
sup128_B2_36484
sup128_HE_36483
sup129_B2_36486_P
sup129_B2_36486
sup129_HE_36485
sup130_B2_36488_P
sup130_B2_36488
sup130_HE_36487
sup131_B2_36490_P
sup131_B2_36490
sup131_HE_36489
sup132_B2_36492_P
sup132_B2_36492
sup132_HE_36491

Supplementary Figure 1

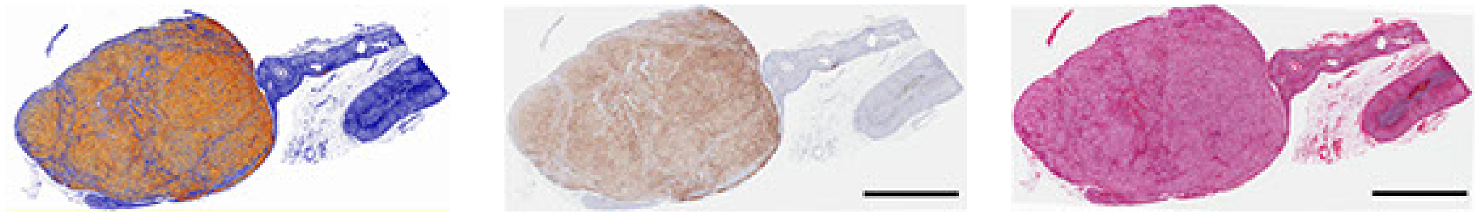

sup133_B2_36494_P sup133_B2_36494 sup133_HE_36493

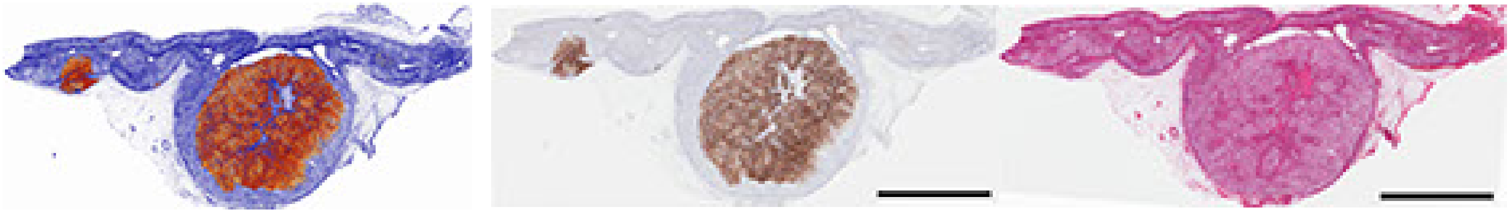

sup134_B2_36496_P sup134_B2_36496 sup134_HE_36495

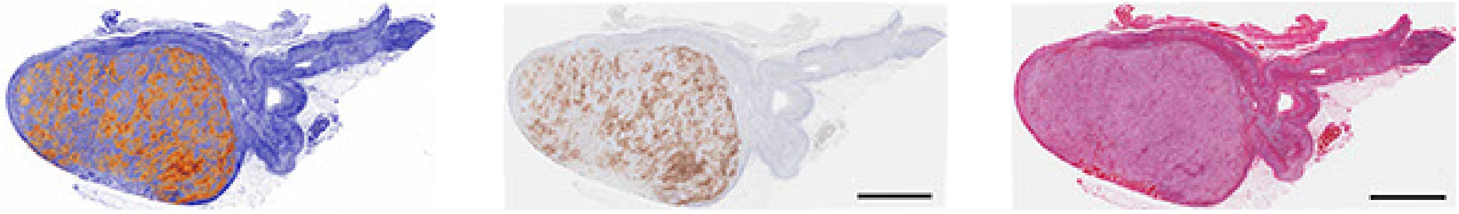

sup135_B2_36498_P sup135_B2_36498 sup135_HE_36497

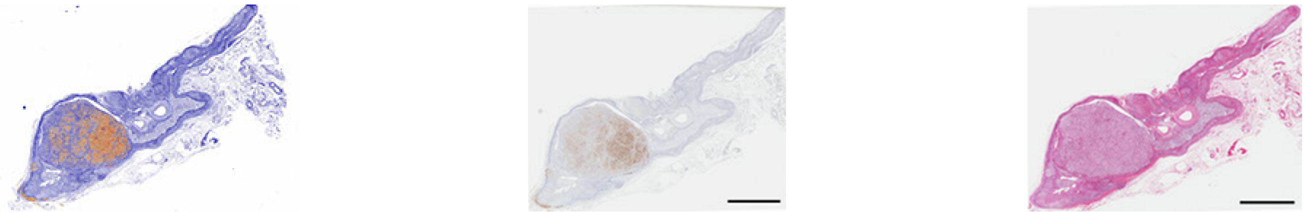

sup136_B2_36500_P sup136_B2_36500 sup136_HE_36499

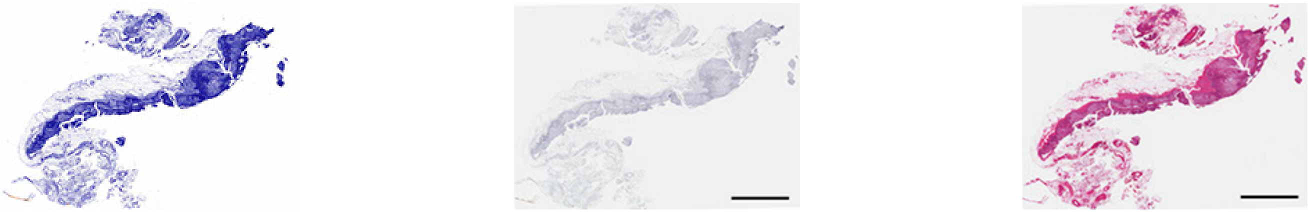

sup137_B2_36502_P sup137_B2_36502 sup137_HE_36501

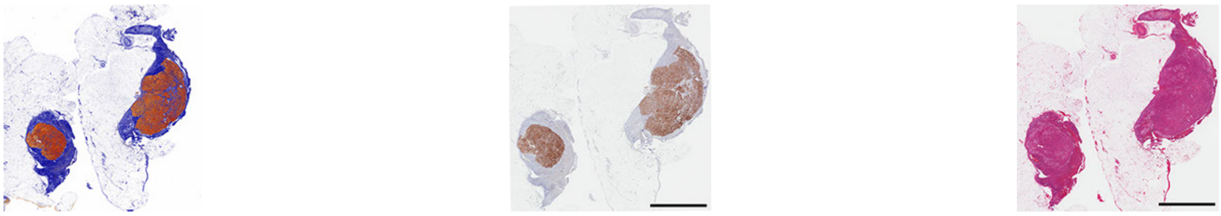

sup138_B2_36504_P sup138_B2_36504 sup138_HE_36503

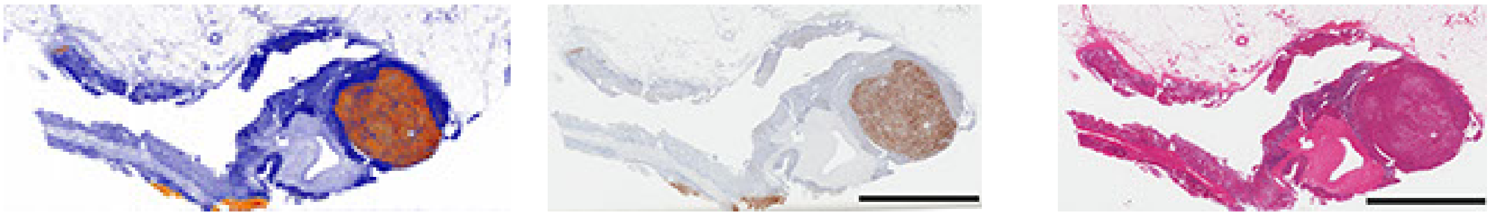

sup139_B2_36506_P sup139_B2_36506 sup139_HE_36505

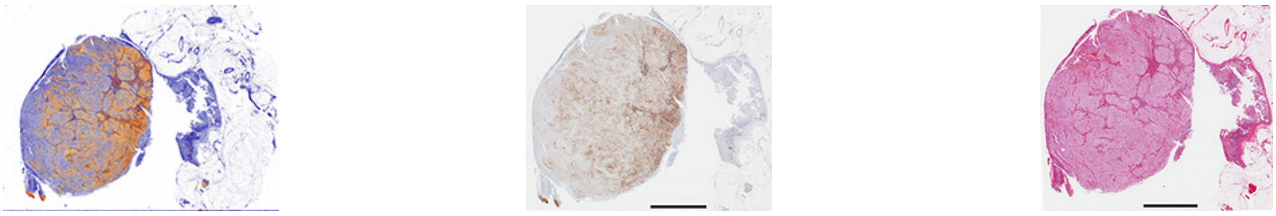

sup140_B2_36508_P sup140_B2_36508 sup140_HE_36507

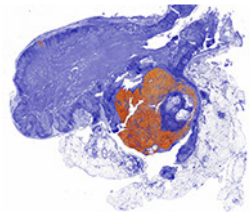
sup141_B2_36510_P

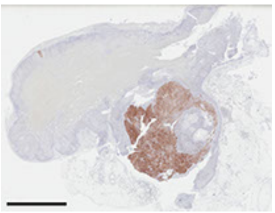
sup141_B2_36510

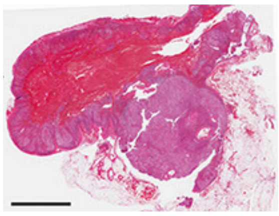
sup141_HE_36509

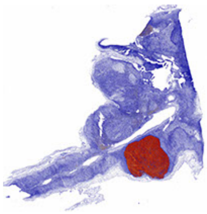
sup142_B2_36512_P

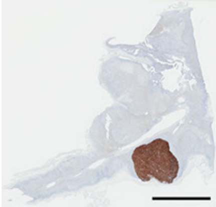
sup142_B2_36512

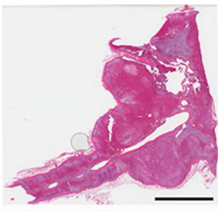
sup142_HE_36511

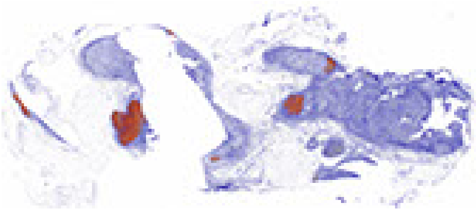
sup143_B2_36514_P

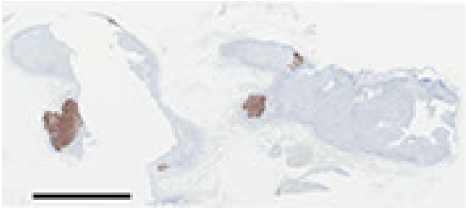
sup143_B2_36514

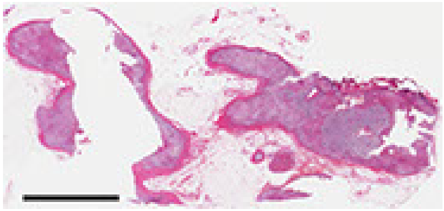
sup143_HE_36513

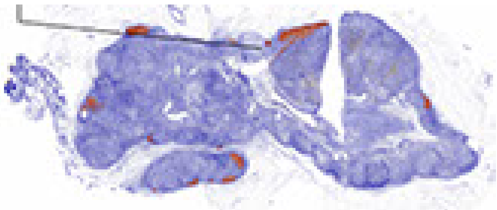
sup144_B2_36516_P

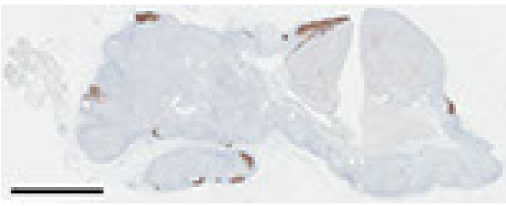
sup144_B2_36516

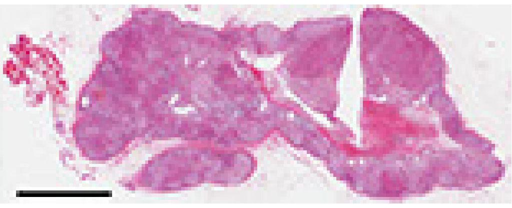
sup144_HE_36515

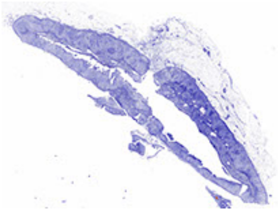
sup145_B2_36518_P

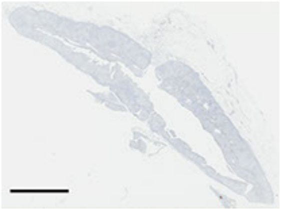
sup145_B2_36518

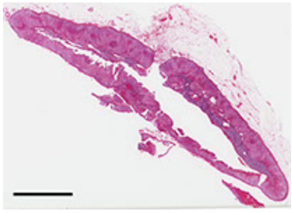
sup145_HE_36517

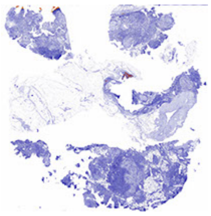
sup146_B2_36520_P

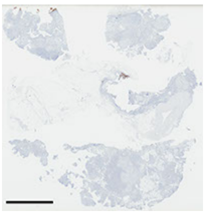
sup146_B2_36520

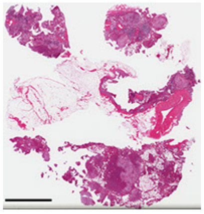
sup146_HE_36519

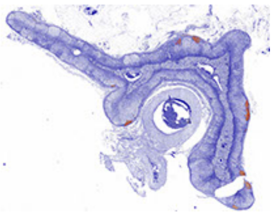
sup147_B2_36522_P

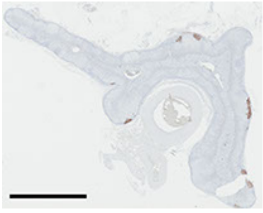
sup147_B2_36522

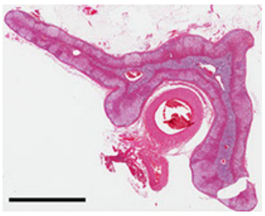
sup147_HE_36521

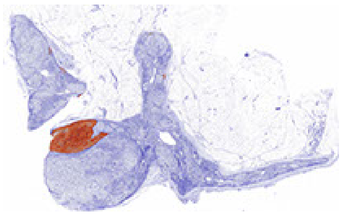
sup148_B2_36524_P

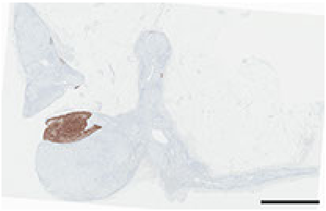
sup148_B2_36524

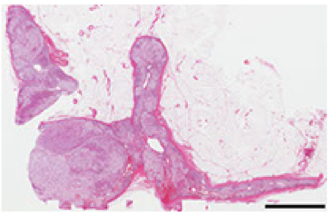
sup148_HE_36523

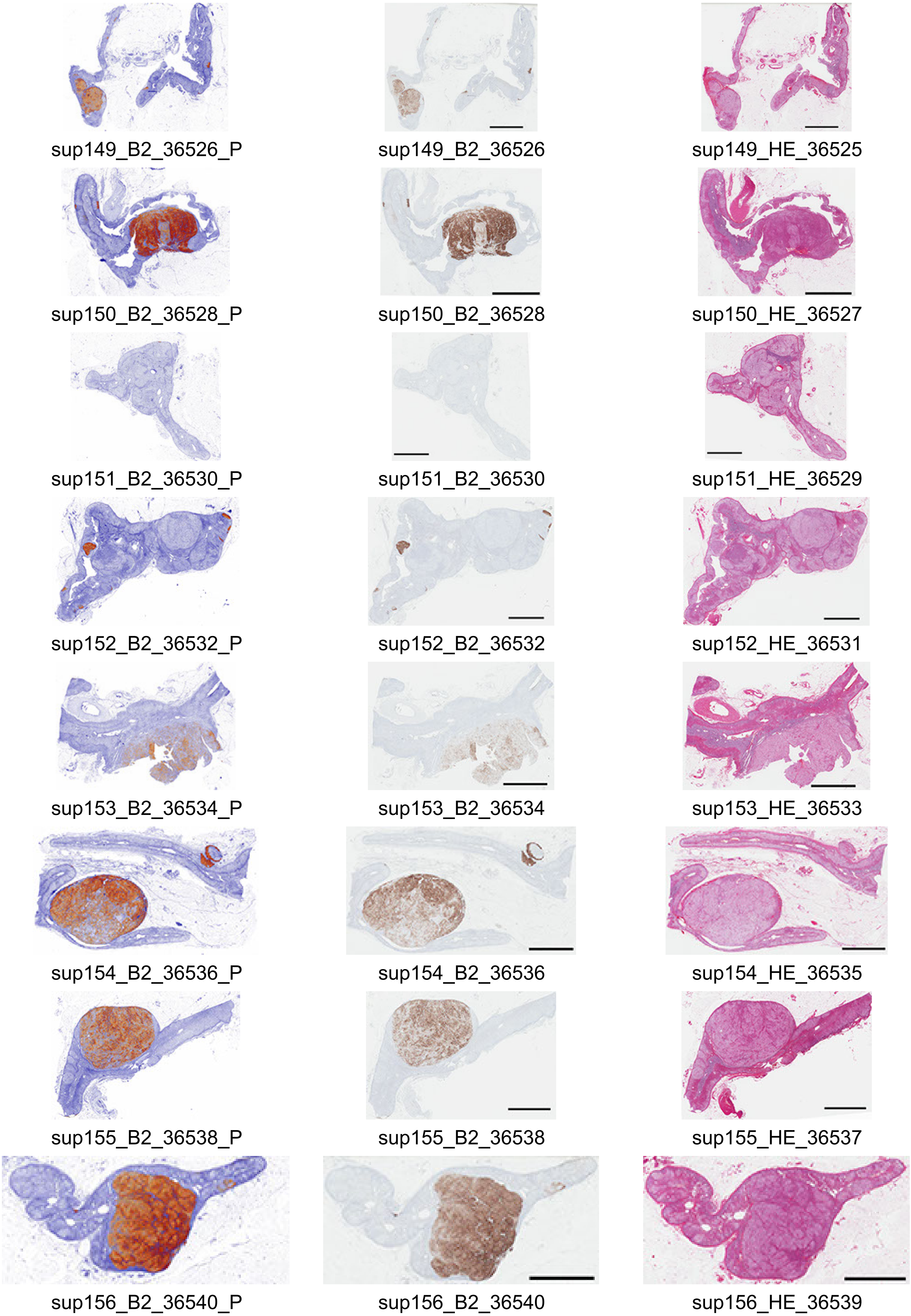
sup149_B2_36526_P
sup149_B2_36526
sup149_HE_36525
sup150_B2_36528_P
sup150_B2_36528
sup150_HE_36527
sup151_B2_36530_P
sup151_B2_36530
sup151_HE_36529
sup152_B2_36532_P
sup152_B2_36532
sup152_HE_36531
sup153_B2_36534_P
sup153_B2_36534
sup153_HE_36533
sup154_B2_36536_P
sup154_B2_36536
sup154_HE_36535
sup155_B2_36538_P
sup155_B2_36538
sup155_HE_36537
sup156_B2_36540_P
sup156_B2_36540
sup156_HE_36539

Supplementary Figure 1

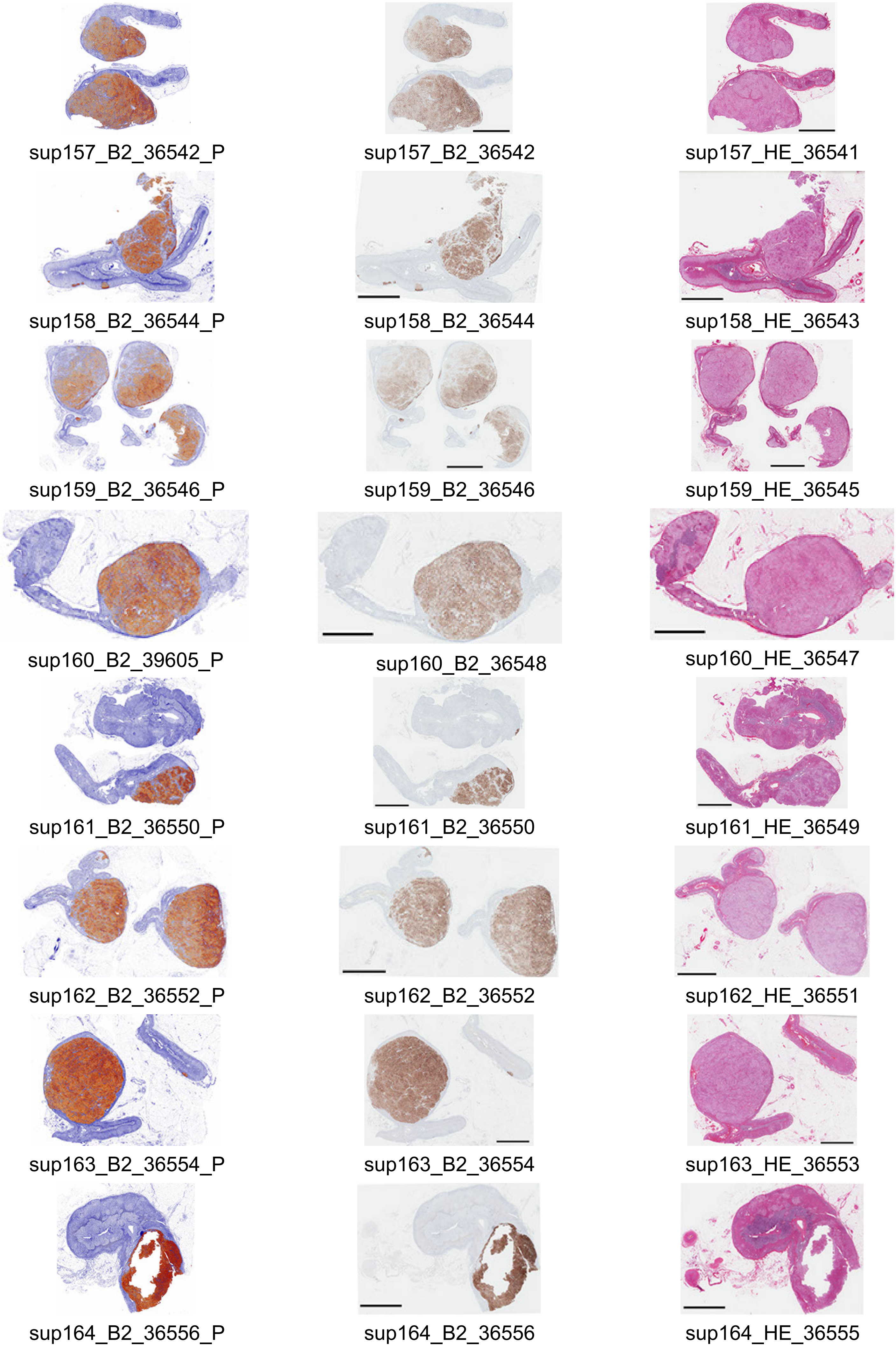

Supplementary Figure 1

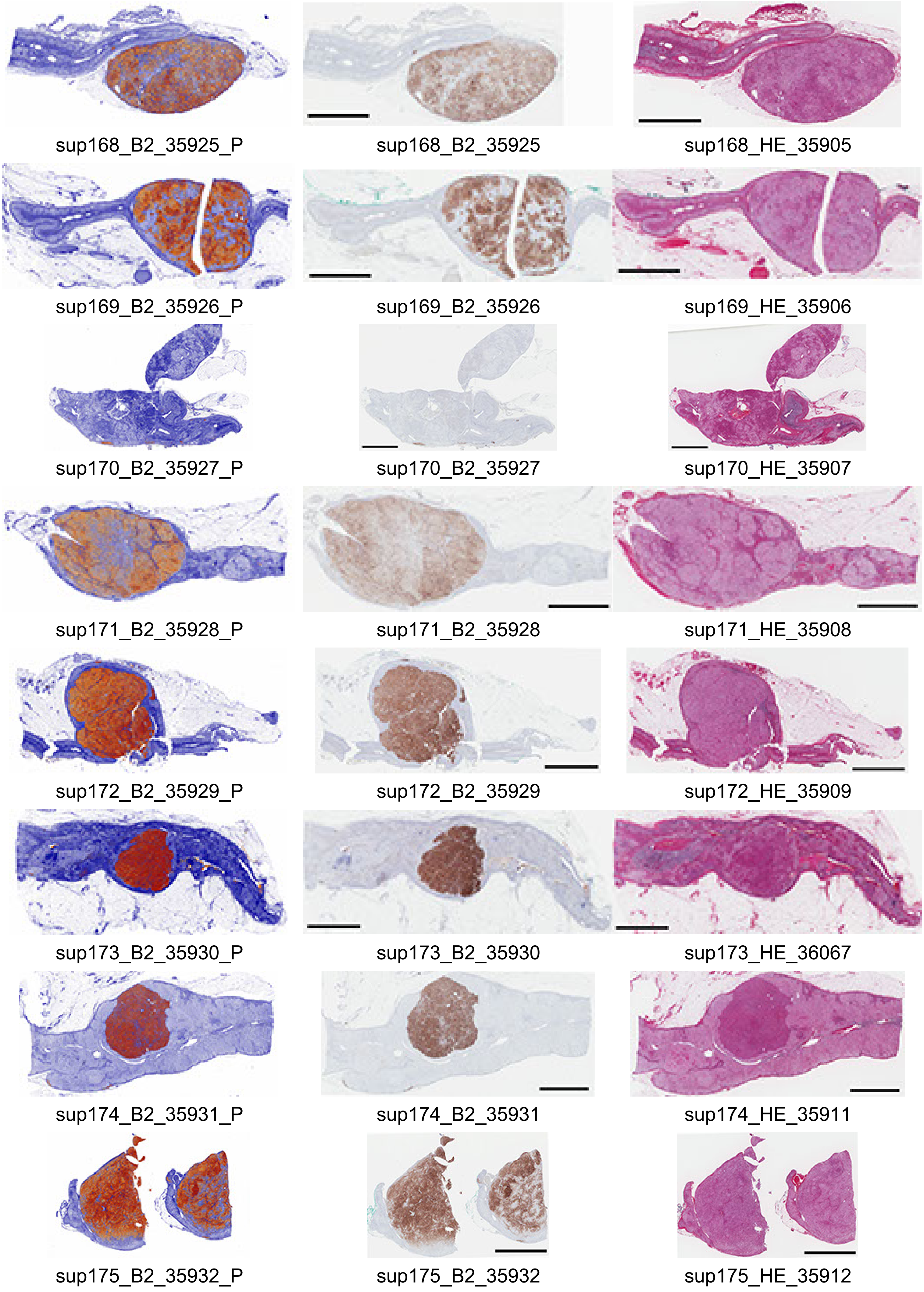

Supplementary Figure 1

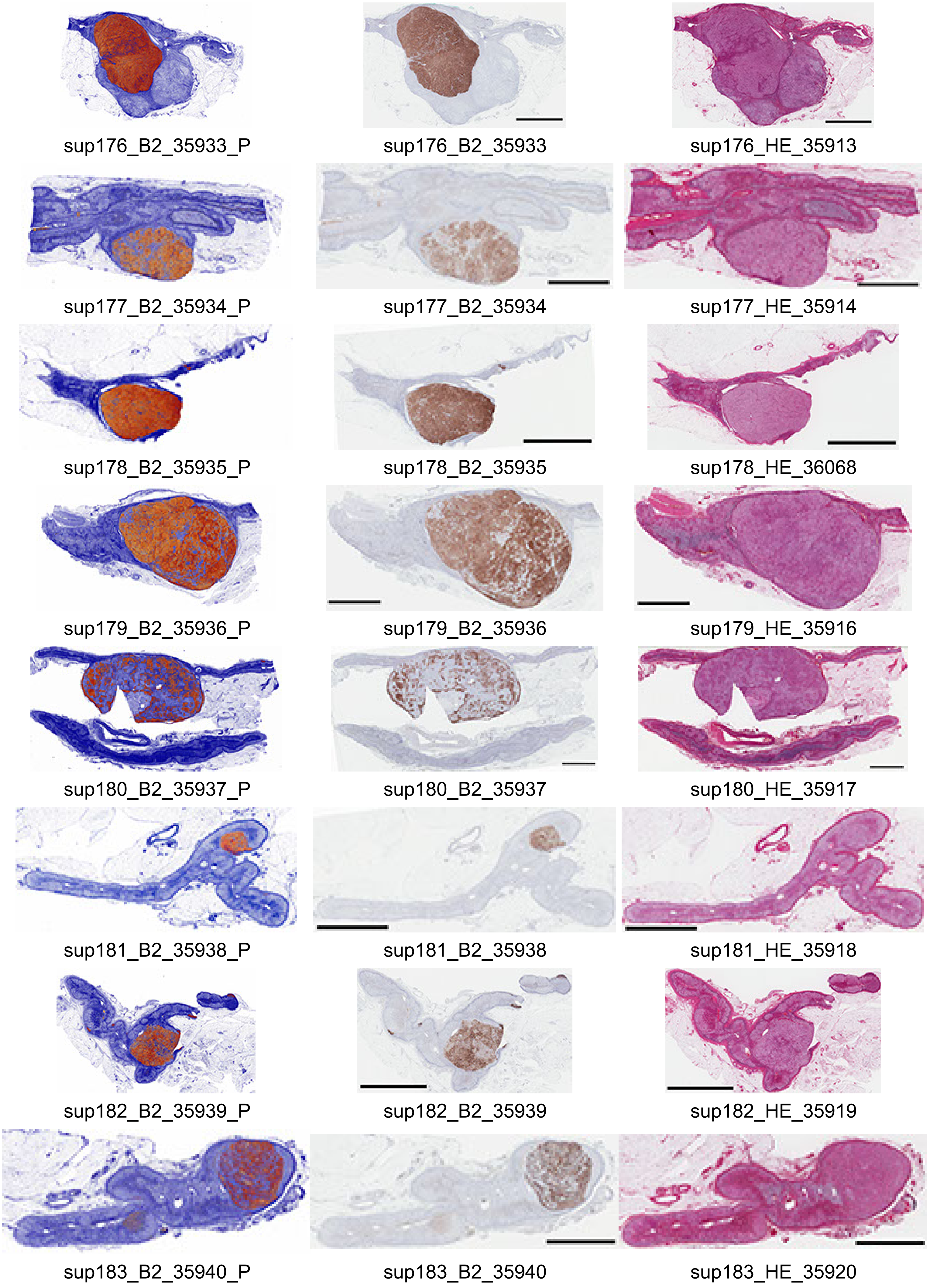

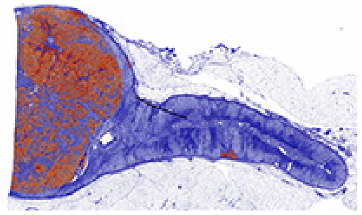
sup184_B2_35941_P
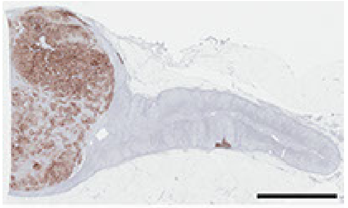
sup184_B2_35941
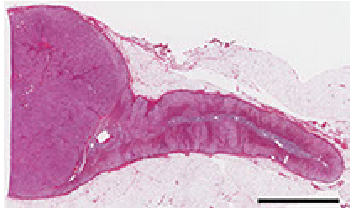
sup184_HE_35921

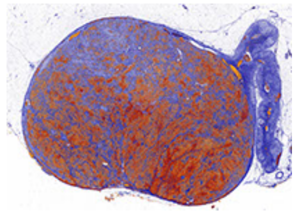
sup185_B2_35942_P
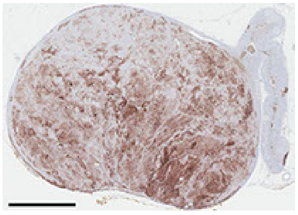
sup185_B2_35942
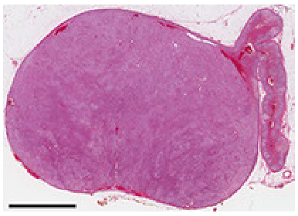
sup185_HE_35922

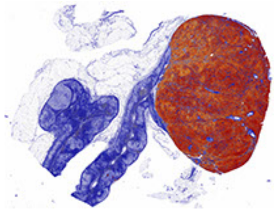
sup186_B2_35943_P
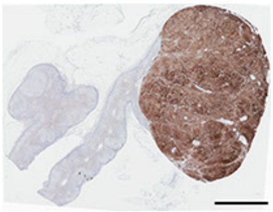
sup186_B2_35943
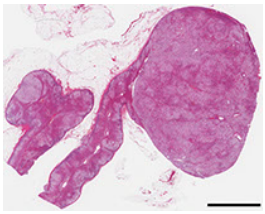
sup186_HE_35923

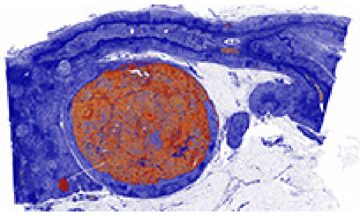
sup187_B2_35944_P
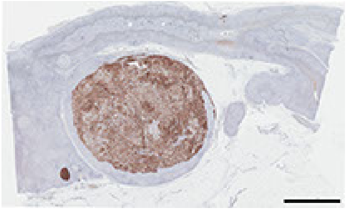
sup187_B2_35944
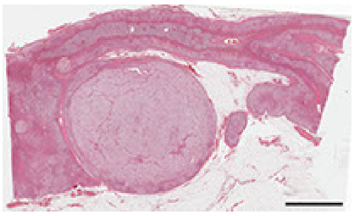
sup187_HE_36851

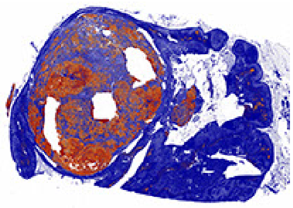
sup188_B2_35965_P
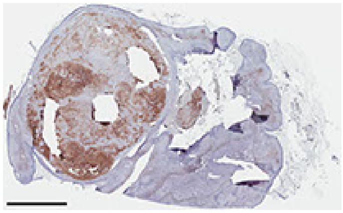
sup188_B2_35965
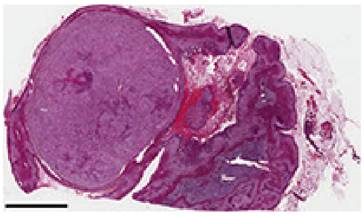
sup188_HE_35945

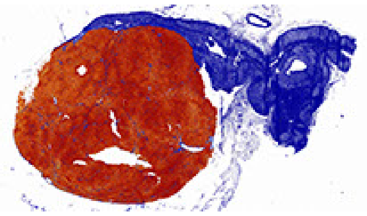
sup189_B2_35966_P
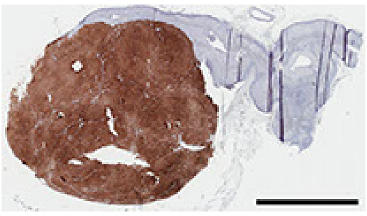
sup189_B2_35966
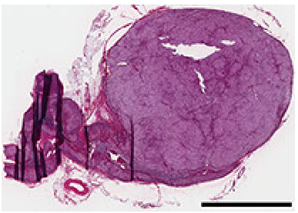
sup189_HE_35946

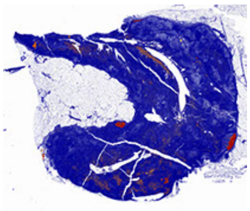
sup190_B2_35967_P
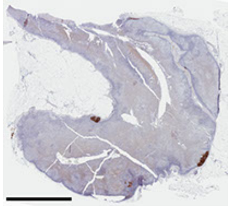
sup190_B2_35967
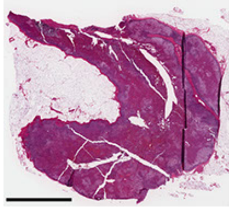
sup190_HE_35947

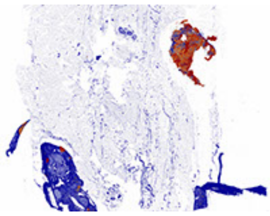
sup191_B2_35968_P
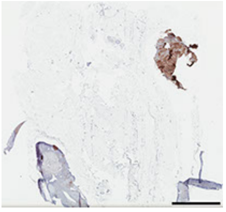
sup191_B2_35968
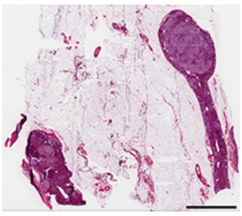
sup191_HE_35948

Supplementary Figure 1

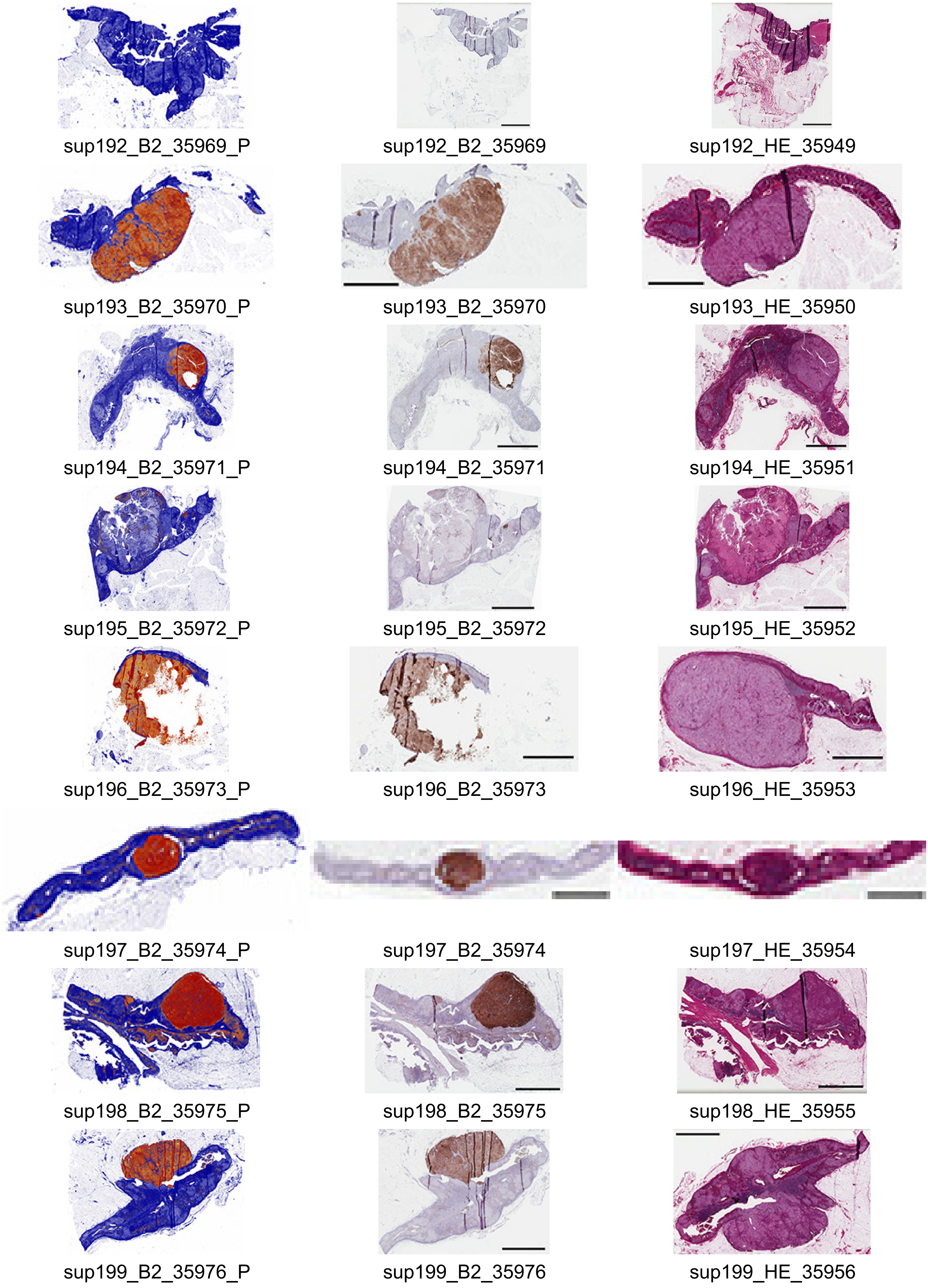

Supplementary Figure 1

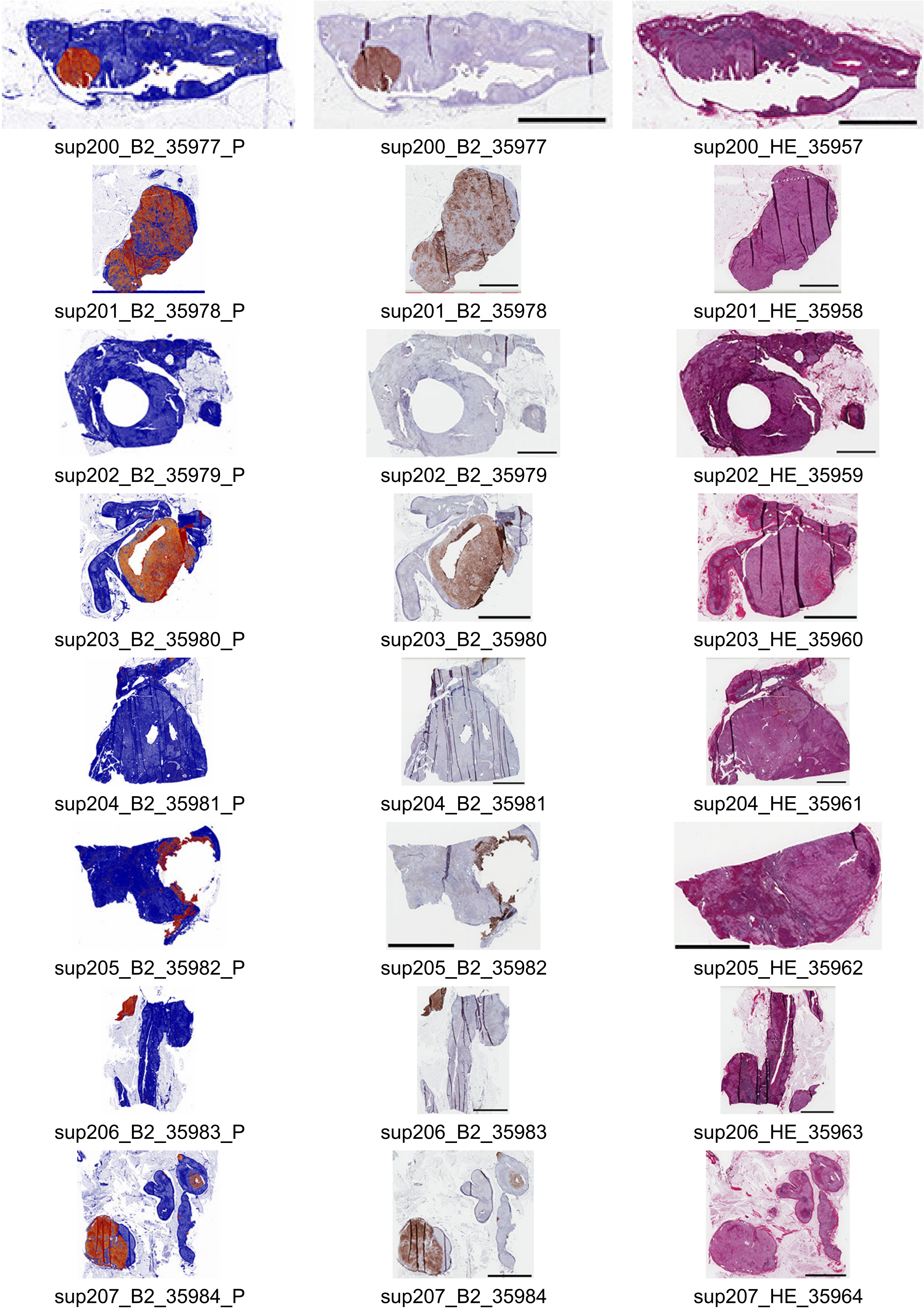

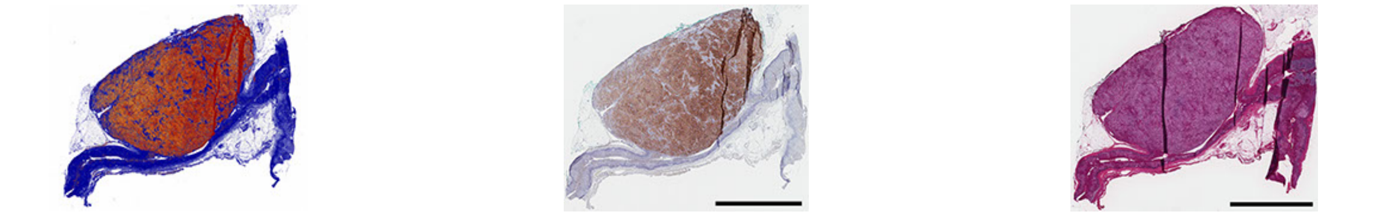

sup208_B2_35987_P sup208_B2_35987 sup208_HE_35985

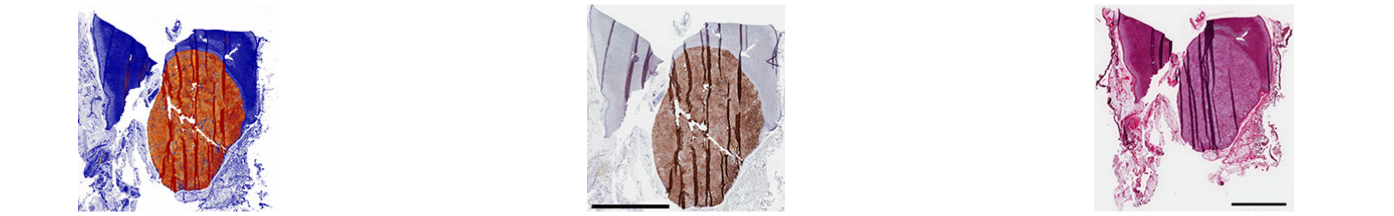

sup209_B2_35988_P sup209_B2_35988 sup209_HE_35986

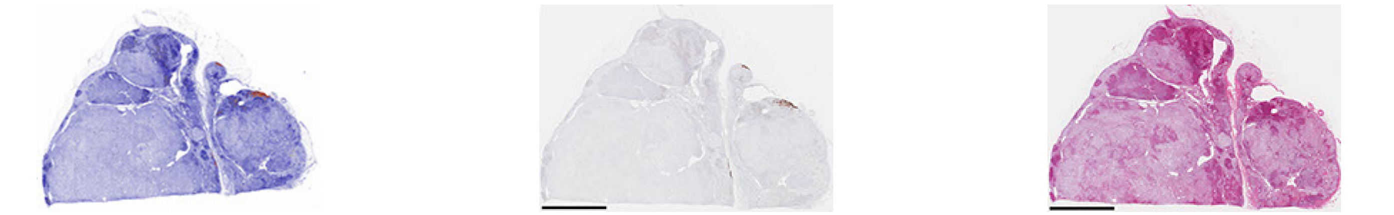

sup210_B2_36041_P sup210_B2_36041 sup210_HE_36015

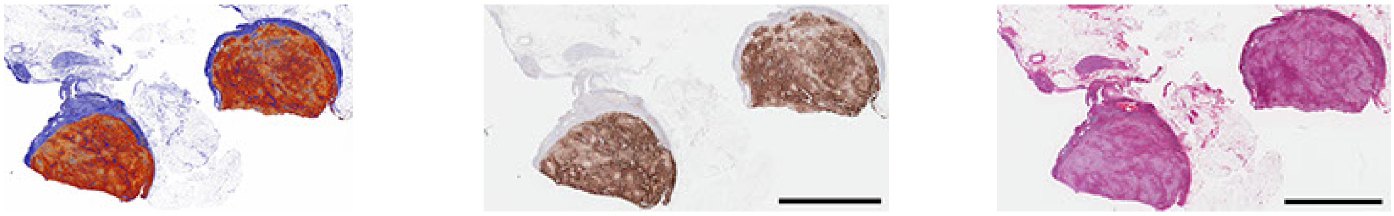

sup211_B2_36042_P sup211_B2_36042 sup211_HE_36016

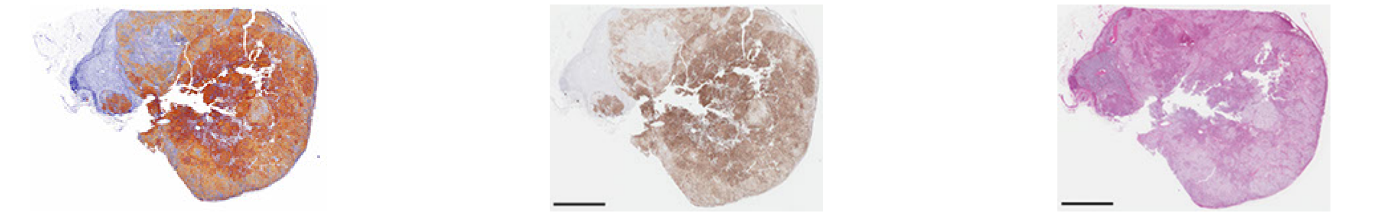

sup212_B2_36043_P sup212_B2_36043 sup212_HE_36017

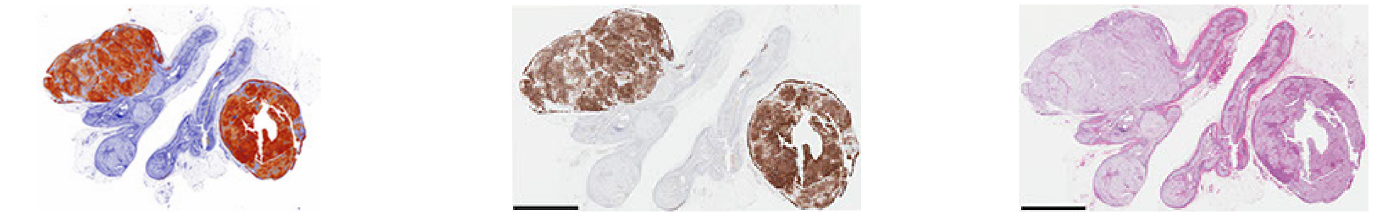

sup213_B2_36044_P sup213_B2_36044 sup213_HE_36018

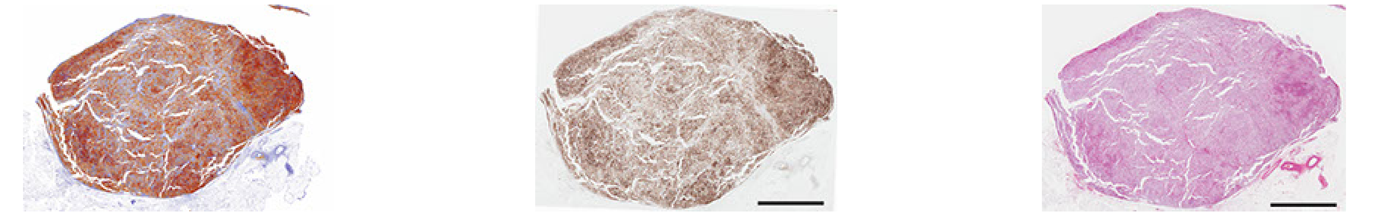

sup214_B2_36045_P sup214_B2_36045 sup214_HE_36019_001

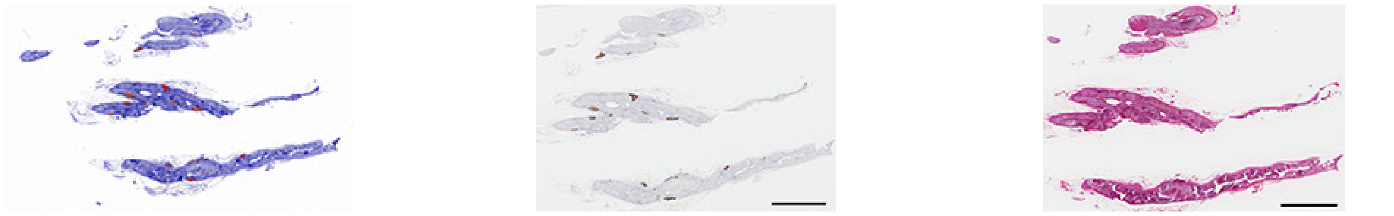

sup215_B2_36046_P sup215_B2_36046 sup215_HE_36073

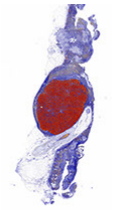
sup216_B2_36047_P

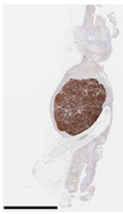
sup216_B2_36047

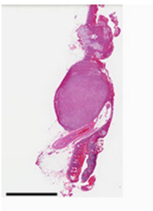
sup216_HE_36021

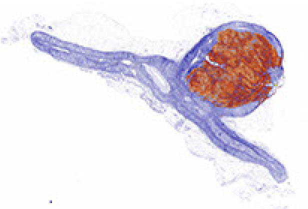
sup217_B2_36048_P

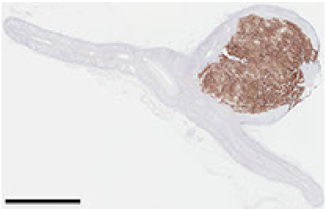
sup217_B2_36048

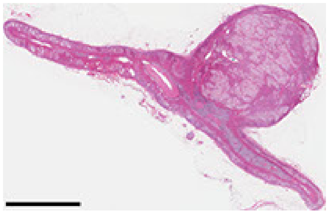
sup217_HE_36022

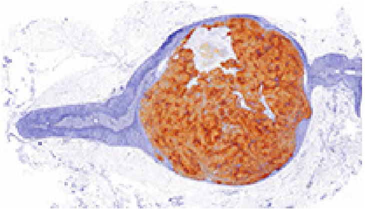
sup218_B2_36049_P

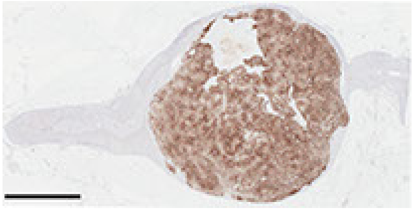
sup218_B2_36049

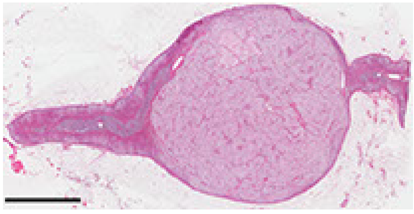
sup218_HE_36023

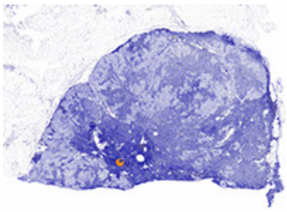
sup219_B2_36050_P

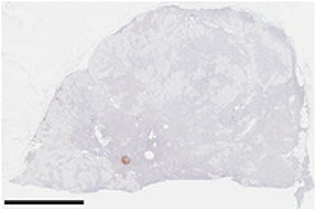
sup219_B2_36050

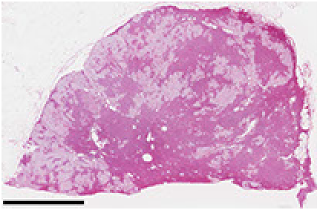
sup219_HE_36024

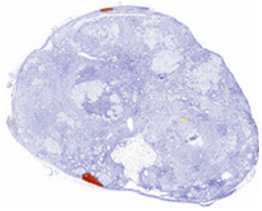
sup220_B2_36051_P

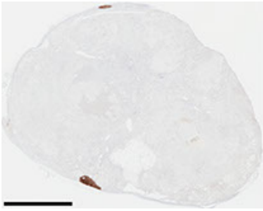
sup220_B2_36051

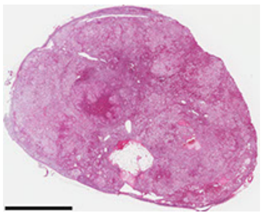
sup220_HE_36025

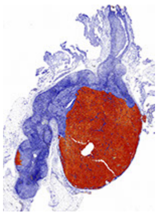
sup221_B2_36052_P

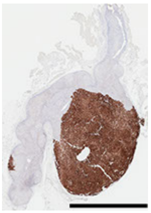
sup221_B2_36052

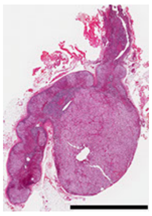
sup221_HE_36026

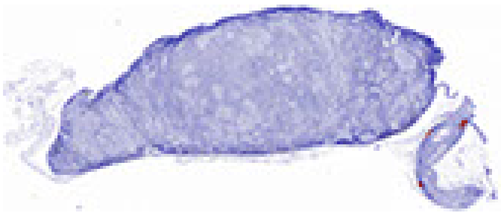
sup222_B2_36053_P

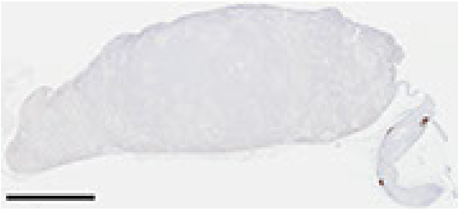
sup222_B2_36053

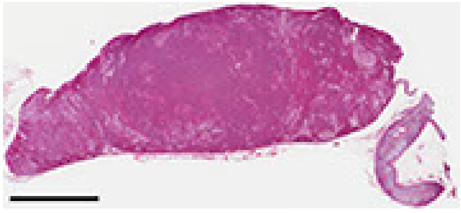
sup222_HE_36027

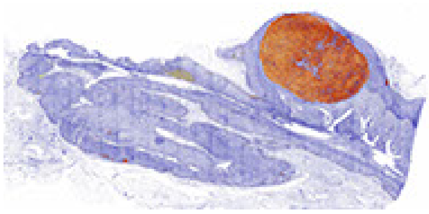
sup223_B2_36054_P

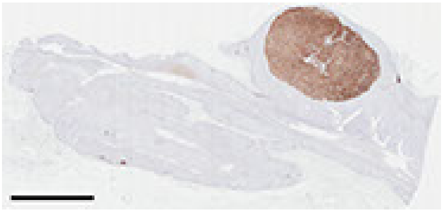
sup223_B2_36054

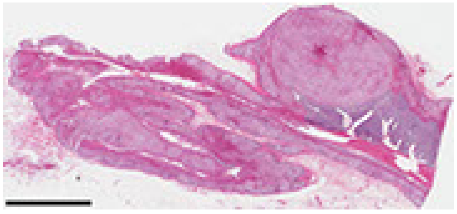
sup223_HE_36028

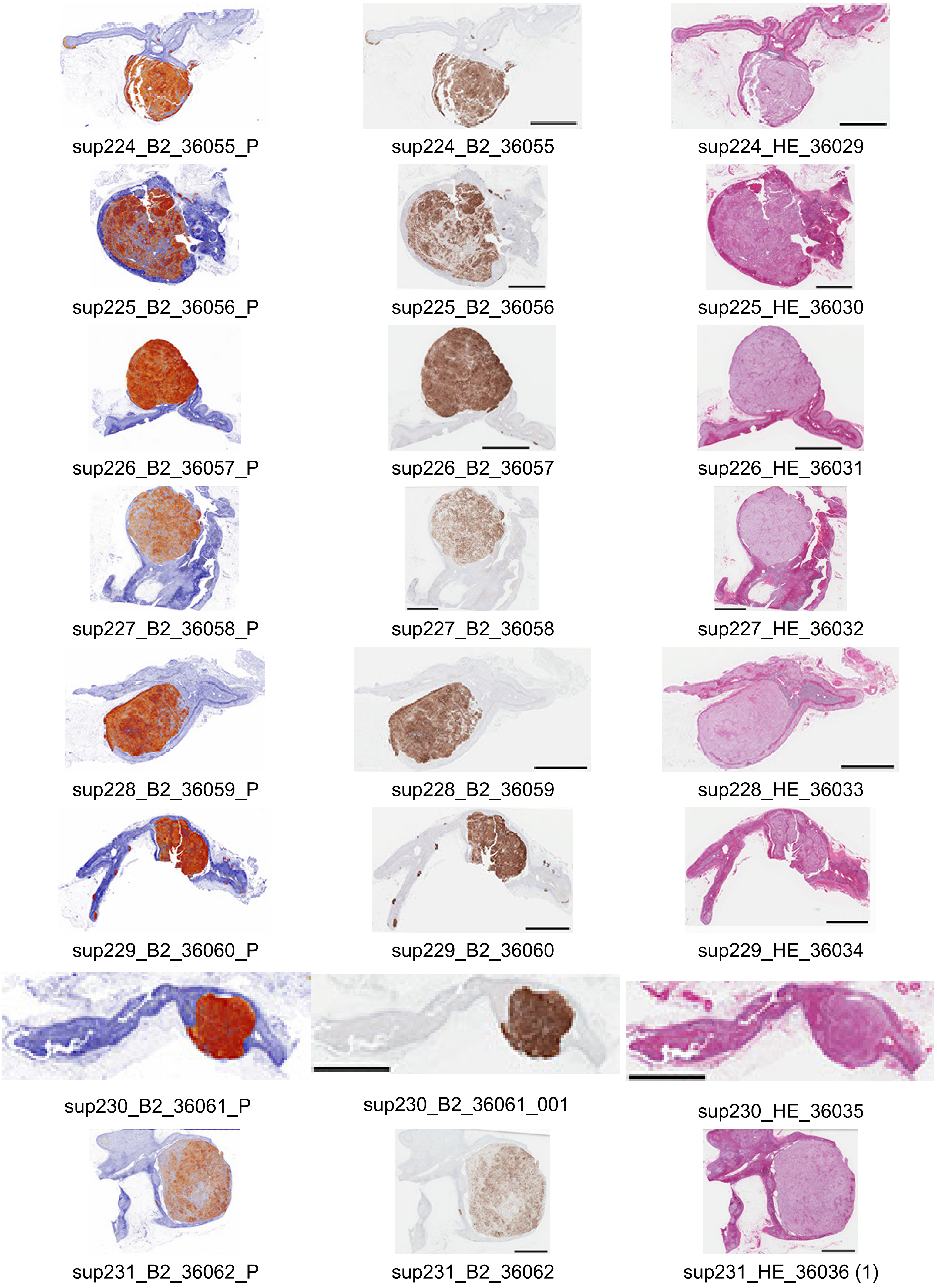
sup224_B2_36055_P
sup224_B2_36055
sup224_HE_36029
sup225_B2_36056_P
sup225_B2_36056
sup225_HE_36030
sup226_B2_36057_P
sup226_B2_36057
sup226_HE_36031
sup227_B2_36058_P
sup227_B2_36058
sup227_HE_36032
sup228_B2_36059_P
sup228_B2_36059
sup228_HE_36033
sup229_B2_36060_P
sup229_B2_36060
sup229_HE_36034
sup230_B2_36061_P
sup230_B2_36061_001
sup230_HE_36035
sup231_B2_36062_P
sup231_B2_36062
sup231_HE_36036 (1)

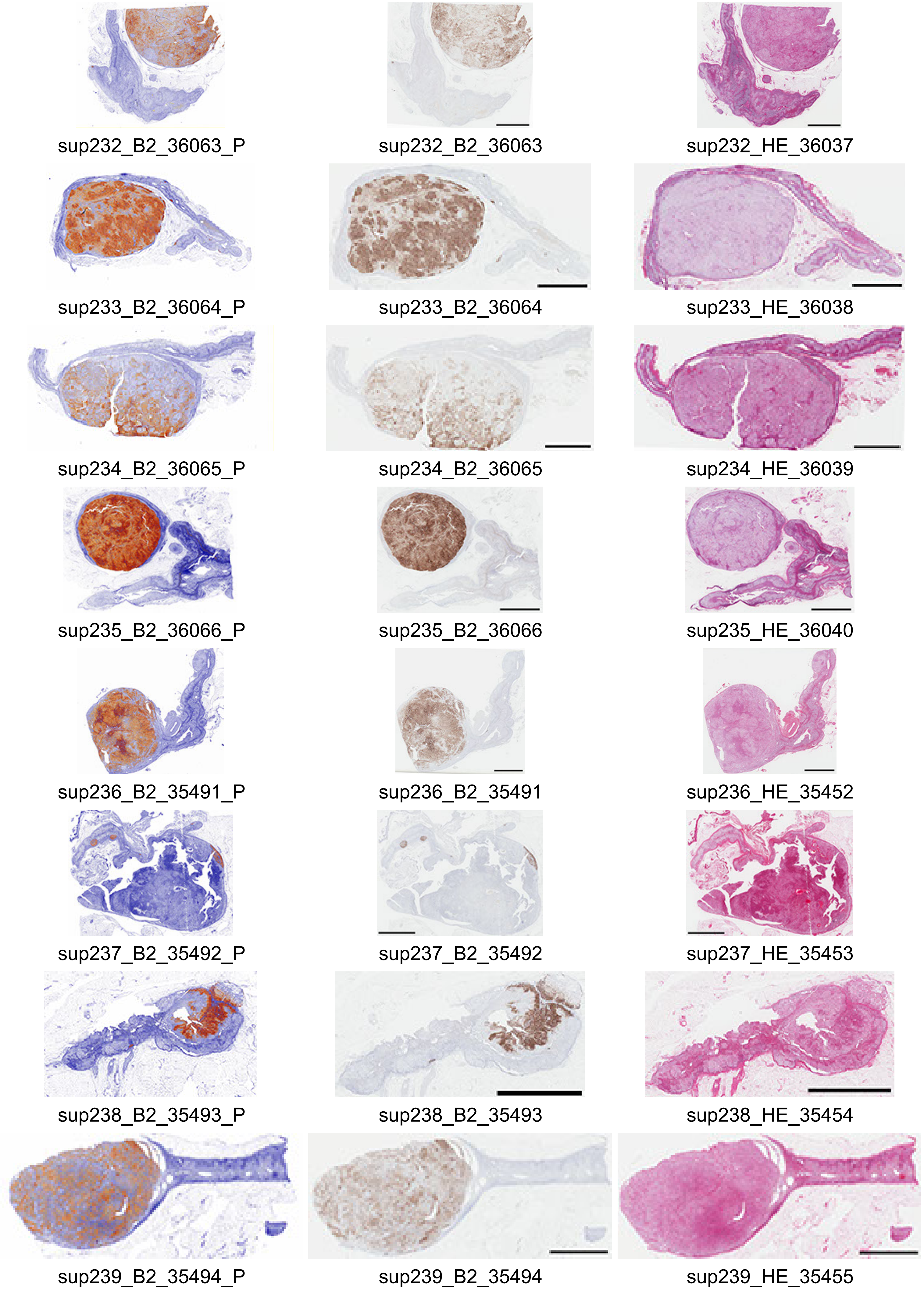
sup232_B2_36063_P
sup232_B2_36063
sup232_HE_36037
sup233_B2_36064_P
sup233_B2_36064
sup233_HE_36038
sup234_B2_36065_P
sup234_B2_36065
sup234_HE_36039
sup235_B2_36066_P
sup235_B2_36066
sup235_HE_36040
sup236_B2_35491_P
sup236_B2_35491
sup236_HE_35452
sup237_B2_35492_P
sup237_B2_35492
sup237_HE_35453
sup238_B2_35493_P
sup238_B2_35493
sup238_HE_35454
sup239_B2_35494_P
sup239_B2_35494
sup239_HE_35455

Supplementary Figure 1

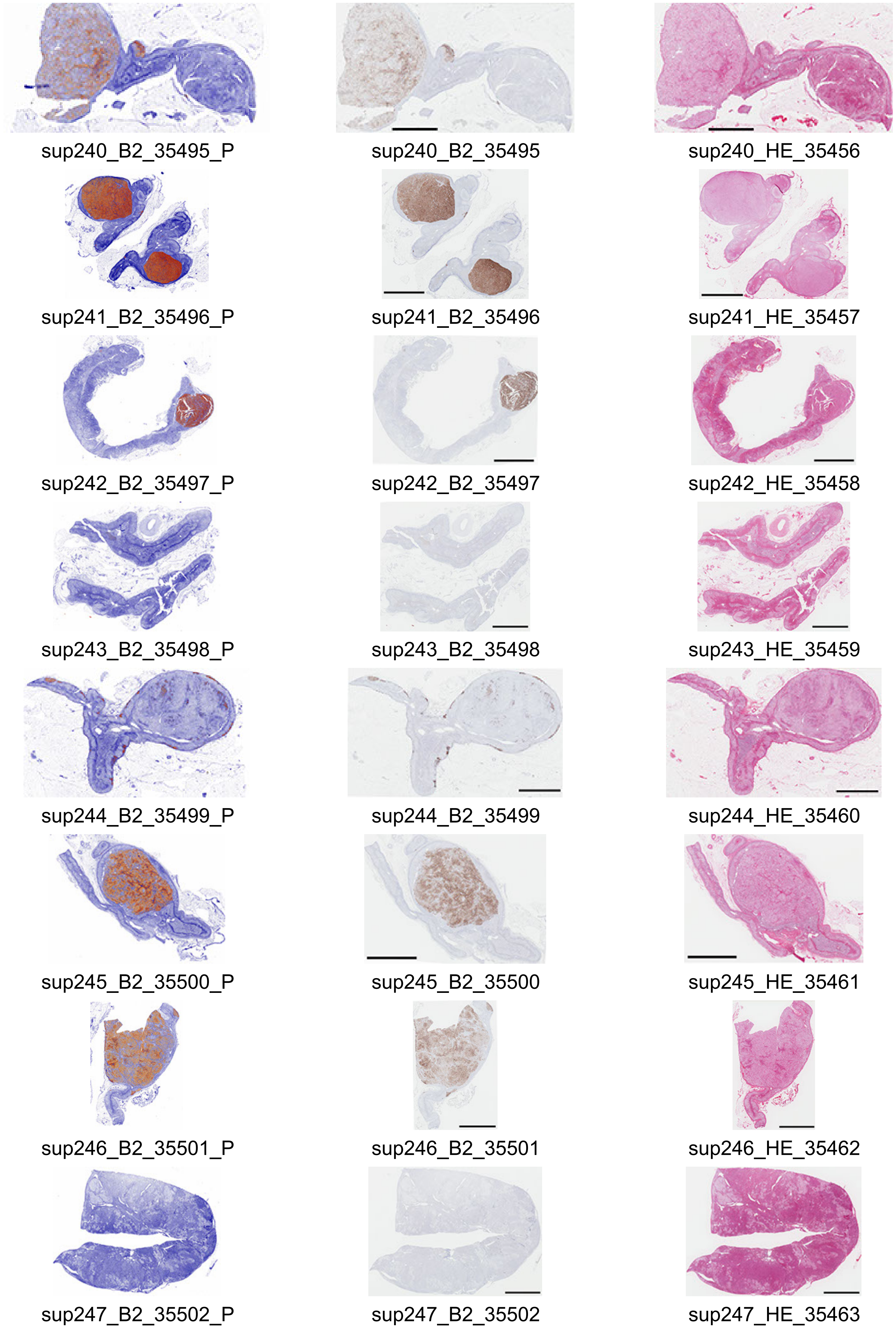

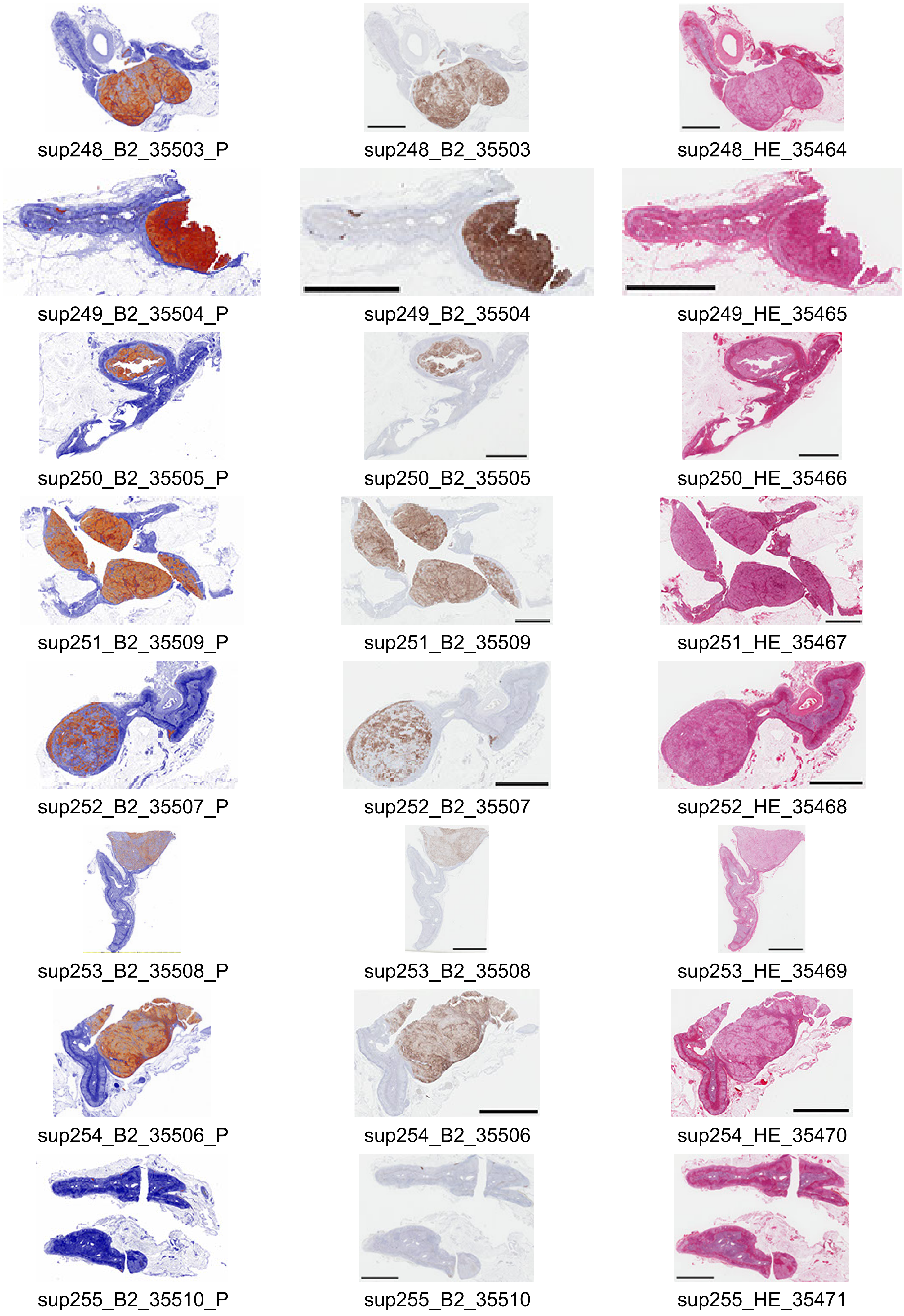
sup248_B2_35503_P
sup248_B2_35503
sup248_HE_35464
sup249_B2_35504_P
sup249_B2_35504
sup249_HE_35465
sup250_B2_35505_P
sup250_B2_35505
sup250_HE_35466
sup251_B2_35509_P
sup251_B2_35509
sup251_HE_35467
sup252_B2_35507_P
sup252_B2_35507
sup252_HE_35468
sup253_B2_35508_P
sup253_B2_35508
sup253_HE_35469
sup254_B2_35506_P
sup254_B2_35506
sup254_HE_35470
sup255_B2_35510_P
sup255_B2_35510
sup255_HE_35471

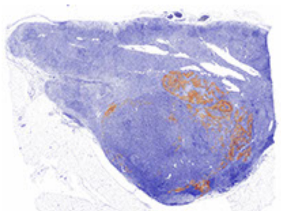
sup256_B2_35511_P
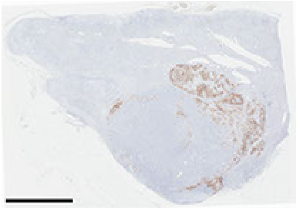
sup256_B2_35511
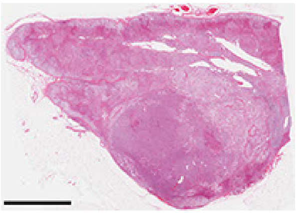
sup256_HE_35472

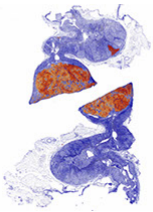
sup257_B2_35512_P
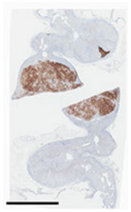
sup257_B2_35512
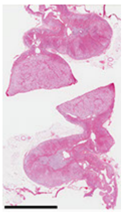
sup257_HE_35473

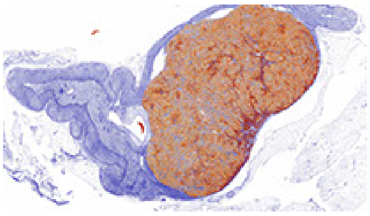
sup258_B2_35513_P
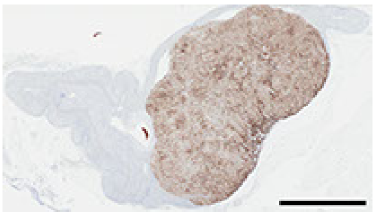
sup258_B2_35513
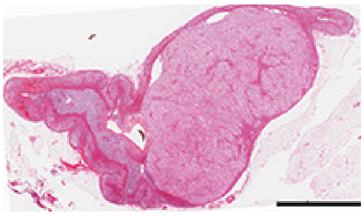
sup258_HE_35474

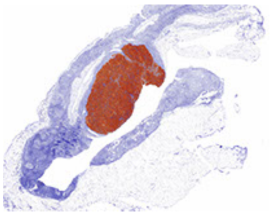
sup259_B2_35514_P
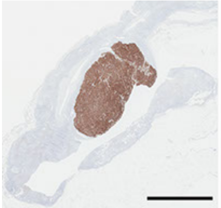
sup259_B2_35514
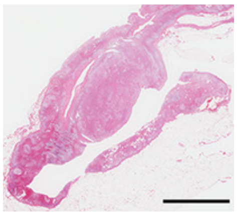
sup259_HE_35475

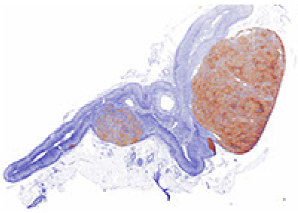
sup260_B2_35515_P
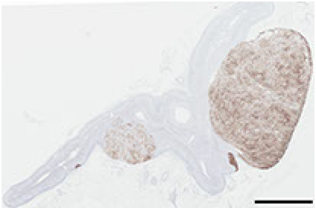
sup260_B2_35515
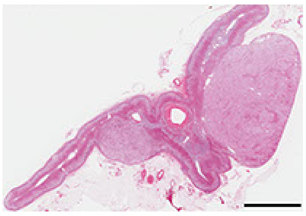
sup260_HE_35476

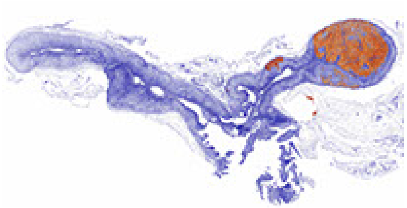
sup261_B2_35516_P
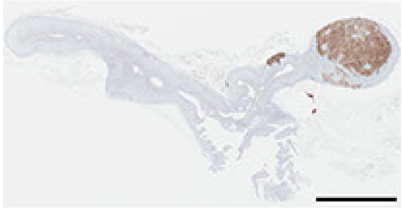
sup261_B2_35516
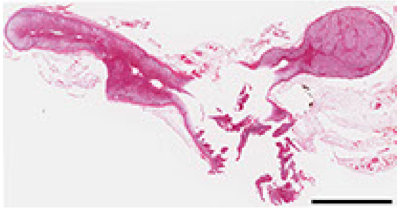
sup261_HE_35481

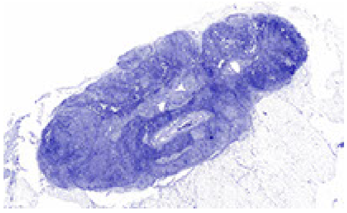
sup262_B2_35517_P
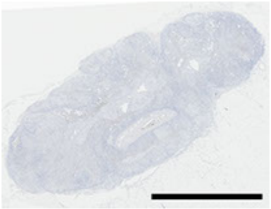
sup262_B2_35517
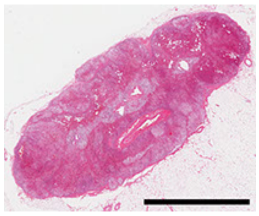
sup262_HE_35480

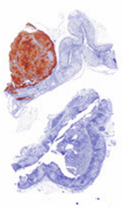
sup263_B2_35518_P
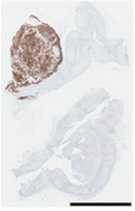
sup263_B2_35518
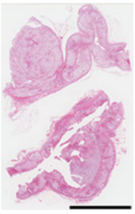
sup263_HE_35479

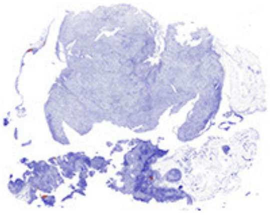
sup264_B2_35519_P
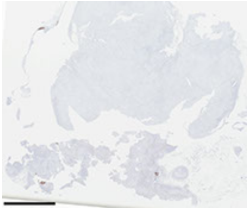
sup264_B2_35519
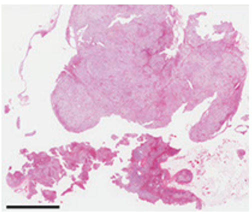
sup264_HE_35478

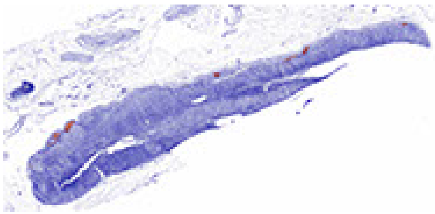
sup265_B2_35520_P
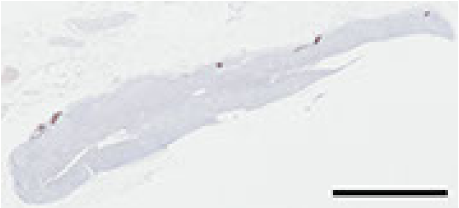
sup265_B2_35520
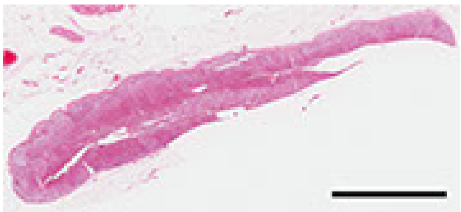
sup265_HE_35477

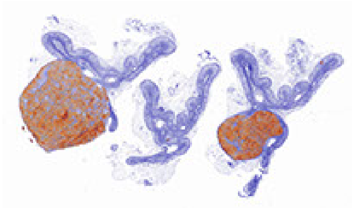
sup266_B2_35521_P
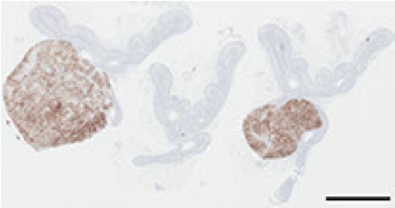
sup266_B2_35521
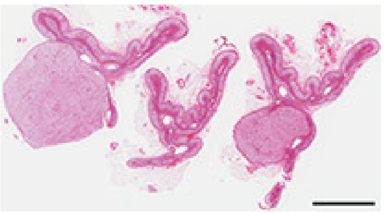
sup266_HE_35482

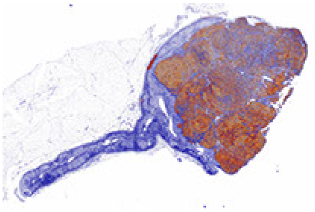
sup267_B2_35522_P
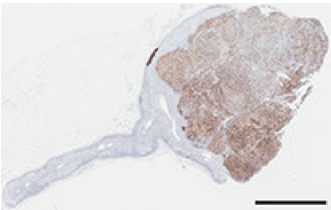
sup267_B2_35522
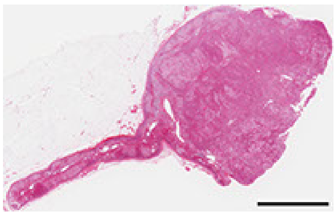
sup267_HE_35483

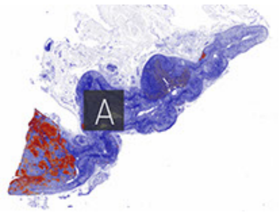

sup268_B2_35523_P
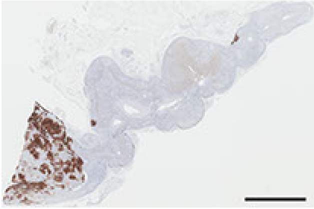
sup268_B2_35523
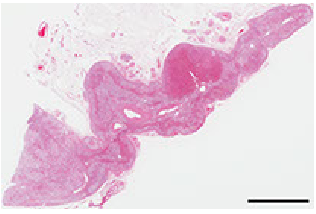
sup268_HE_35484

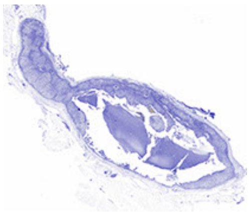
sup269_B2_35524_P
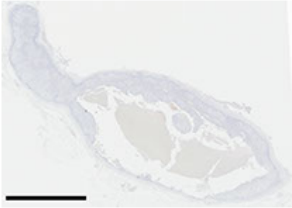
sup269_B2_35524
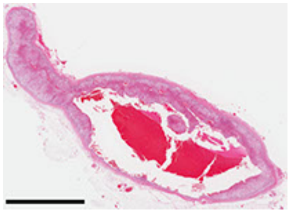
sup269_HE_35485

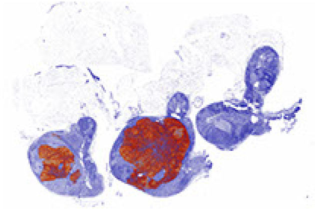
sup270_B2_35525_P
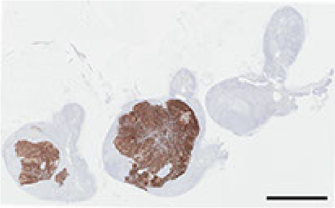
sup270_B2_35525
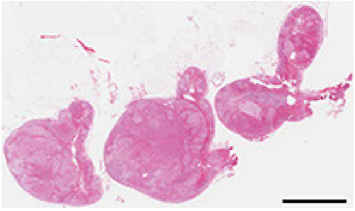
sup270_HE_35486

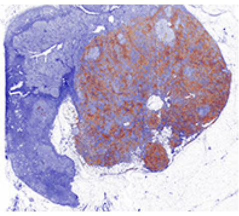
sup271_B2_35526_P
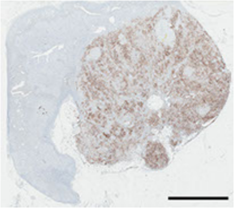
sup271_B2_35526
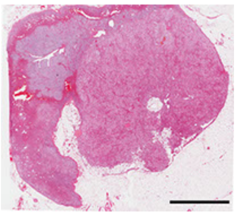
sup271_HE_35487

Supplementary Figure 1

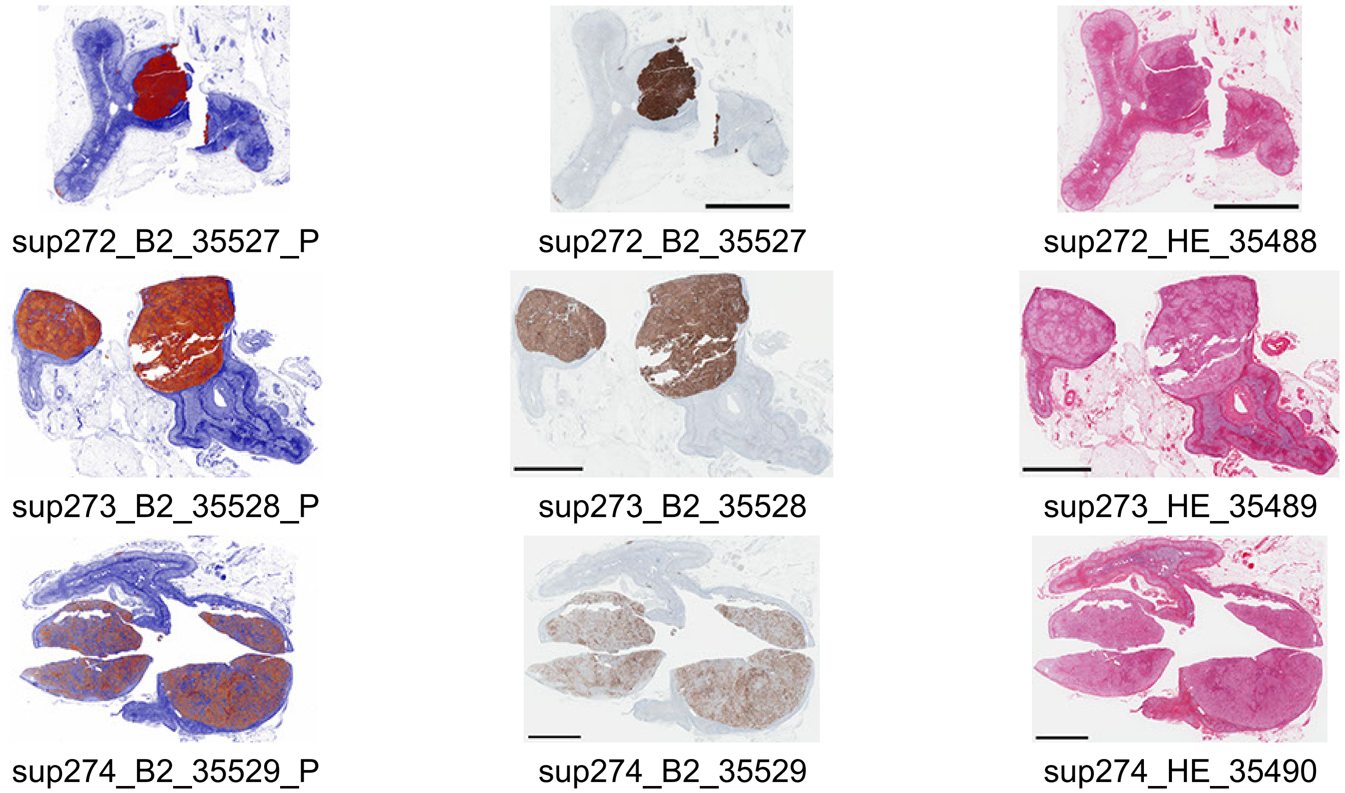

Supplement: Supplementary file 1 — Supplementary Figure 1 [file 41440_2020_579_MOESM1_ESM.pdf]
