# Supplementary figures and images for: Diverse pathological lesions of primary aldosteronism and their clinical significance
Source: Hypertens Res. 2021 Jan 12;44(5):498–507. doi: 10.1038/s41440-020-00579-w (PMC8099725; doi:10.1038/s41440-020-00579-w)

**Supplementary Figure 2**

**A biochemical outcome**

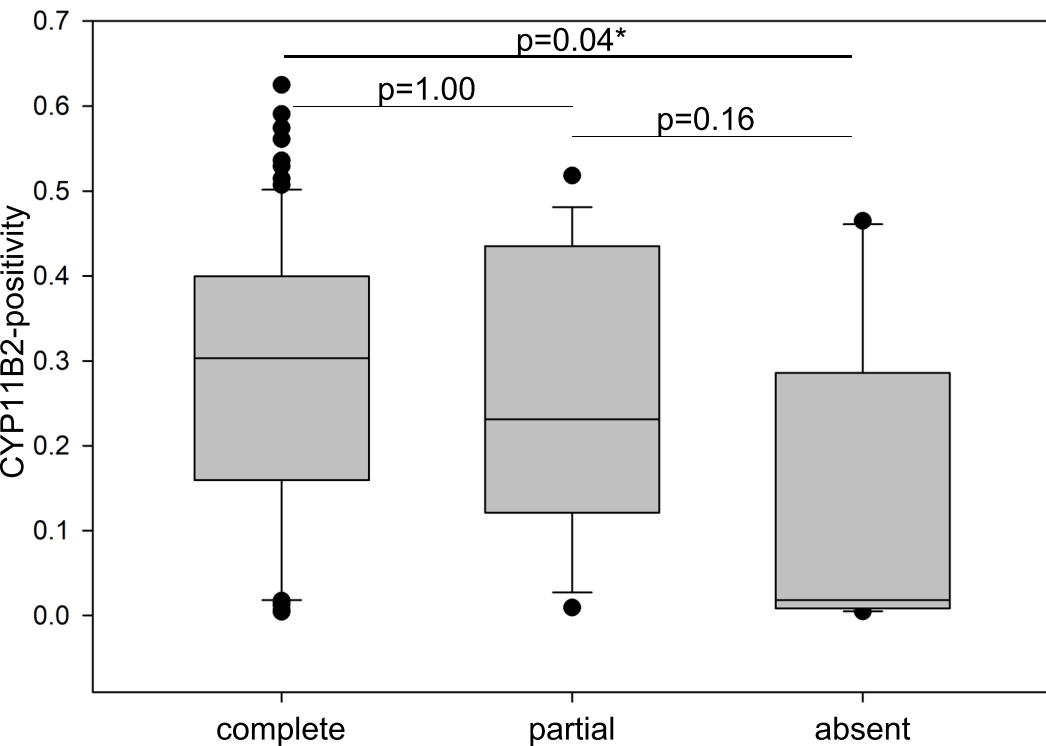

**B clinical outcome**

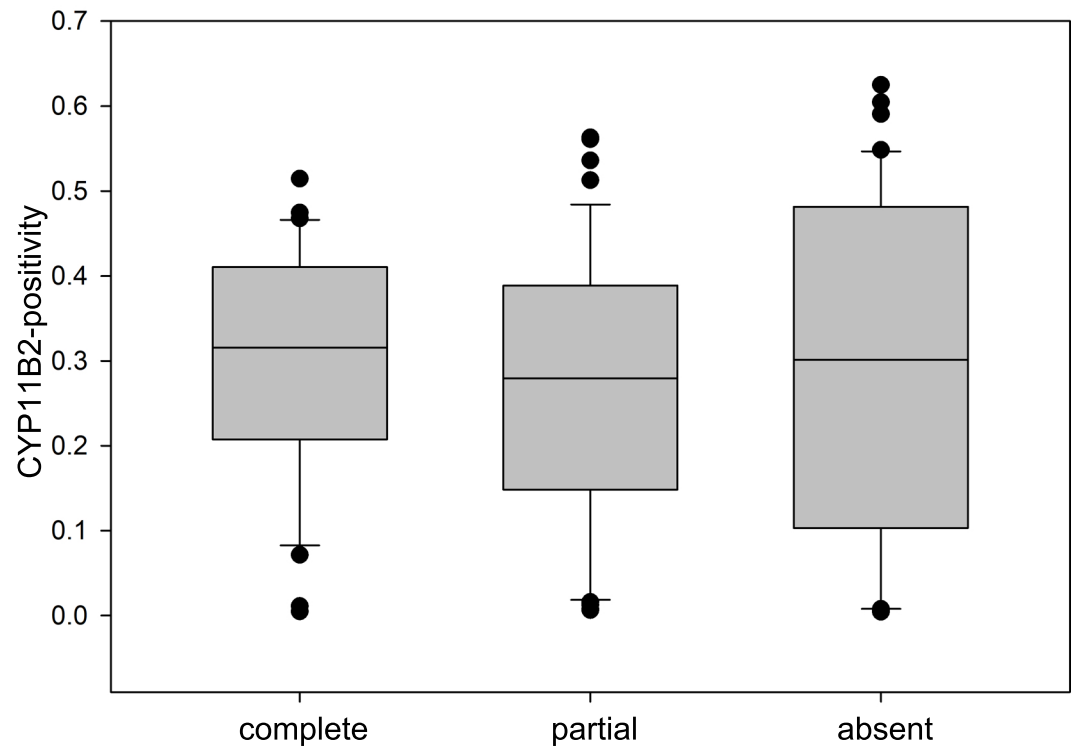

Supplement: Supplementary file 2 — Supplementary Figure 2 [file 41440_2020_579_MOESM2_ESM.pdf]

Supplementary Figure 3

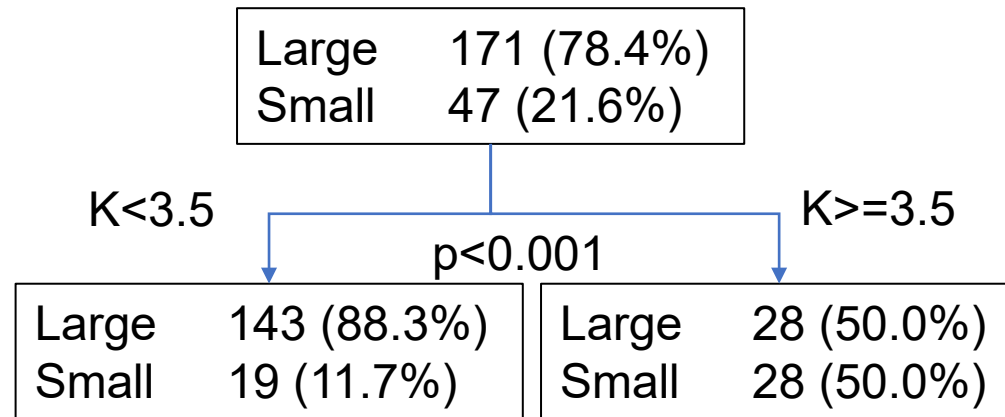

Supplement: Supplementary file 3 — Supplementary Figure 3 [file 41440_2020_579_MOESM3_ESM.pdf]
