# Supplementary material for: Diverse pathological lesions of primary aldosteronism and their clinical significance
Source: Hypertens Res. 2021 Jan 12;44(5):498–507. doi: 10.1038/s41440-020-00579-w (PMC8099725; doi:10.1038/s41440-020-00579-w)
Supplement: Supplementary file 4 — Supplementary Materials and Methods [file 41440_2020_579_MOESM4_ESM.docx]

# Supplementary Materials and Methods

## Ethics

The participating institutions included Kyoto Medical Center (IRB approval#: #15-039, n = 39), Osaka University (#16163, n = 39), St. Marianna University (#3165, n = 34), Oita University (#909, n = 26), Kyushu University (#29-113, n = 22), Sapporo City General Hospital (#H27-052-268, n = 22), Kyoto University (#R0335, n = 20), Tenri Hospital (#811, n = 14), Tottori University (#1608A092, n = 11), Uwajima City Hospital (#172-90, n = 9), Tokyo Medical and Dental University (#2198, n = 6), and Saitama Medical University International Medical Center (SIMC) (approval#: 16-093, pathological analyses).

## Case selection

Since available patient information and samples varied among institutions, cases were selected based on each institution’s criteria as follows: all PA patients who were unilaterally adrenalectomized between January 1st, 2006 and December 31st, 2016 at the National Hospital Organization Kyoto Medical Center and St. Marianna University; those between August 25th, 2006 and September 2nd, 2016 at Osaka University; those between September 29th, 2010 and January 20th, 2017 at Tenri Hospital; those between January 1st, 2006 and December 31st, 2015 at Oita University; those between January 1st, 2011 and December 31st, 2016 at Tottori University; those between April 1st, 2014 and March 31st, 2016 at Kyushu University; and those between January 1st, 2013 and December 31st, 2017 at Uwajima City Hospital as well as randomly selected PA patients who were unilaterally adrenalectomized between January 1st, 2009 and December 31st, 2016 at Sapporo City General Hospital; those between January 1st, 2006 and December 31st, 2016 at Kyoto University; and those between January 1st, 2009 and December 31st, 2014 at Tokyo Medical and Dental University.

## Removal of 20 pathological samples

Among the 242 stained cases, 20 were excluded from subsequent statistical analyses due to poor sample preparation (n = 9, “poor sample preparation” in the column “initial pathological diagnosis” of Supplementary Table 1), PA lesions not present in the section (presumably outside of the sectioned plane, n = 3), and no CYP11B2-positive cells on each section (“no CYP11B2-positive cells”, n=8). Regarding the 9 poorly prepared samples, 2 from Institution #WJ-014 (n = 2, Cases 215 [sup #59] and 239 [#146]) showed severely fragmented adrenals, which was presumably caused at the time of surgery, and were incompatible for re-sampling. Seven samples from Institution #035 (n = 7, Cases 194 [sup #191], 195 [#192], 198 [#195], 199 [#196], 207 [#204], 208 [#205], and 209 [#206]) showed cracking, folds, and peeling, which were presumably due to a substandard sectioning procedure. These samples were not re-sectioned and were simply excluded from analyses to avoid possible experimental bias due to a non-concurrent staining process.

## Initial pathological diagnosis

Initial pathological diagnoses of the remaining cases (n = 222) were performed by an author (KN) as follows (the 2^nd^ and 3^rd^ columns of Supplementary Figure 1): (i) apparent CYP11B2-positive tumors larger than 6 mm were diagnosed as APA, (ii) subcapsular CYP11B2-positive cell clusters smaller than 1.5 mm were diagnosed as APCCs, (iii) apparent tumors in H&E, which did not contain CYP11B2-positive cells, were diagnosed as non-functional tumors (although they may be CYP11B1-expressing tumors), (iv) CYP11B2-positive lesions between 1.5 – 6 mm were diagnosed as APCC, pAATL, or APA based on their morphology, (v) lesions with intermediate characteristics were designated with both types of lesions with a slash in between, e.g., pAATL/APA and pAATL/APCCs, (vi) if multiple lesions were identified in a section, all were listed with separation using semi-colons, and (vii) the zona glomerulosa, which was apparently normal and without a lesion, was shown as ZG if layered CYP11B2 expression was present (Supplementary Table 1). All stained images were labeled with a SIMC urology pathology identification number (sup #); staining methods, i.e., H&E (labeled as HE) or CYP11B2 (labeled as B2); and five-digit image identification number at SIMC.

## Intensity calculation of hypertensive agents in each patient

The preoperative usage of anti-hypertensive agents was quantified as the intensity of antihypertensives, which was calculated as previously reported (Supplementary Table 2) (Williams, et al. 2017). Briefly, the amount of each anti-hypertensive agent taken per day was divided by the defined daily dose for the agent indicated in the Anatomical Therapeutic Chemical classification system and the Defined Daily Dose index (ATC/DDD index 2018, <https://www.whocc.no/atc_ddd_index>, Last updated: 2017-12-20, underlined numbers in Supplementary Table 2). The sum of the divided values of all anti-hypertensives in each patient was then calculated (intensity of antihypertensives). For example, Case 10 was administered 40 mg/day of azilsartan medoxomil (defined daily dose: 40 mg), 10 mg/day of amlodipine (5 mg), 1 mg/day of indapamide (2.5 mg), and 2 mg/day of doxazosin (4 mg), and, thus, the intensity of antihypertensives for Case 10 was calculated as 40/40 + 10/5 +1/2.5 + 2/4 = 3.9. The defined daily doses of azelnidipine, alacepril, bunazosin, and guanabenz were not available in the ATC/DDD index 2018; therefore, we defined their daily doses as the median values of the recommended doses described in their package inserts in Japan (Supplementary Data 2, available in Japanese only [parts describing suggested daily doses are marked by blue boxes and the translation is provided]; find the median values, i.e., their daily doses, in Supplementary Table 2).

## CYP11B2 positivity

CYP11B2 positivity was calculated using the Positive Pixel Count Algorithm (also see <https://www.leicabiosystems.com/digital-pathology/analyze/ihc/aperio-positive-pixel-count-algorithm>). Blue, yellow, orange, and red pixels indicate negative, weak-positive, positive, and strong-positive pixels of CYP11B2 immunohistochemistry (2^nd^ column of Supplementary Figure 1). The calculated positivity of each case is shown in Supplementary Table 1.

**Supplementary References**

Hayashi T, Zhang Z, Al-Eyd G, Sasaki A, Yasuda M, Oyama M, Gomez-Sanchez CE, Asakura H, Seki T, Mukai K, et al. 2019 Expression of aldosterone synthase CYP11B2 was inversely correlated with longevity. *J Steroid Biochem Mol Biol* **191** 105361.

Williams TA, Lenders JWM, Mulatero P, Burrello J, Rottenkolber M, Adolf C, Satoh F, Amar L, Quinkler M, Deinum J, et al. 2017 Outcomes after adrenalectomy for unilateral primary aldosteronism: an international consensus on outcome measures and analysis of remission rates in an international cohort. *Lancet Diabetes Endocrinol* **5** 689-699.

# Supplementary Figure Legends

## Supplementary Figure 1. Images of histological data from all cases.

Images in the 1^st^, 2^nd^, and 3^rd^ columns indicate false-color images (artificially colored images) of CYP11B2 immunohistochemistry (Hayashi, et al. 2019), CYP11B2 immunohistochemistry, and H&E staining, respectively. Scale bars indicate 5 mm. All images are labeled with a Saitama Medical University International Medical Center Urology Pathology (sup) ID, method of staining (B2=CYP11B2 immunohistochemistry, HE=H&E), and image scope file number, followed by a “P” to indicate a Positive Pixel Count Algorithm image of CYP11B2 immunohistochemistry as applicable. The relationships between the case number and sup ID are shown in Supplementary Table 1.

## Supplementary Figure 2. A classification tree predicting patients with small PA lesions by a serum K threshold of 3.5 mEq/L.

The upper node was separated into the two lower nodes based on the serum K value (<3.5 mEq/L vs. ≥3.5 mEq/L). The p value was calculated by Fisher’s exact test.
