# Supplementary material for: Diverse pathological lesions of primary aldosteronism and their clinical significance
Source: Hypertens Res. 2021 Jan 12;44(5):498–507. doi: 10.1038/s41440-020-00579-w (PMC8099725; doi:10.1038/s41440-020-00579-w)
Supplement: Supplementary file 8 — Supplementary Data 2 [file 41440_2020_579_MOESM8_ESM.pdf]

Supplementary Data 2 [package inserts of azelnidipine, alacepril, bunazosin, and guanabenz]

※※2017年４月改訂（第３版）
※2014年11月改訂

添付文書情報

日本標準商品分類番号	８７２１４９

持続性Ca拮抗剤

※ 処方箋医薬品注1)

※日本薬局方　アゼルニジピン錠

アゼルニジピン錠８mg「NP」
アゼルニジピン錠16mg「NP」

AZELNIDIPINE TABLETS

※貯　　法：室温・遮光保存
使用期限：容器等に記載
注　　意：「取扱い上の注意」参照

	錠８mg	錠16mg
承認番号	22500AMX00279	22500AMX00280
薬価収載	2013年６月	2013年６月
販売開始	2013年６月	2013年６月

禁忌（次の患者には投与しないこと）
1.妊婦又は妊娠している可能性のある婦人（「6.妊婦、産婦、授乳婦等への投与」の項参照）
2.本剤の成分に対し過敏症の既往歴のある患者
※※ 3.アゾール系抗真菌剤（外用剤を除く）（イトラコナゾール、ミコナゾール、フルコナゾール、ホスフルコナゾール、ボリコナゾール）、HIVプロテアーゼ阻害剤（リトナビル含有製剤、サキナビル、インジナビル、ネルフィナビル、アタザナビル、ホスアンプレナビル、ダルナビル含有製剤）、コビシスタット含有製剤、オムビタスビル・パリタプレビル・リトナビルを投与中の患者（「3.相互作用」の項参照）

【組成・性状】

1.組成

販売名	アゼルニジピン錠８mg「NP」	アゼルニジピン錠16mg「NP」
有効成分（１錠中）	日本薬局方　アゼルニジピン	
	８mg	16mg
添加物	結晶セルロース、低置換度ヒドロキシプロピルセルロース、軽質無水ケイ酸、ポリソルベート80、メグルミン、ヒドロキシプロピルセルロース、カルメロースカルシウム、ステアリン酸マグネシウム	

2.製剤の性状

	アゼルニジピン錠８mg「NP」	アゼルニジピン錠16mg「NP」
外形	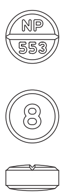	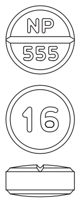
形状	淡黄白色の割線入り素錠	
大きさ 直径（mm）	7.0	9.0
大きさ 厚さ（mm）	3.4	4.2
大きさ 重量（mg）	140	280
識別コード	NP-553	NP-555

【効能・効果】

高血圧症

【用法・用量】

通常、成人にはアゼルニジピンとして８～16mgを１日１回朝食後経口投与する。なお、１回８mgあるいは更に低用量から投与を開始し、症状により適宜増減するが、１日最大16mgまでとする。

translation: 8 - 16 mg/day

※※【使用上の注意】

1.慎重投与（次の患者には慎重に投与すること）
1)重篤な肝・腎機能障害のある患者［本剤は肝臓で代謝される。また一般に重篤な腎機能障害のある患者では、降圧に伴い腎機能が低下する可能性がある。］
2)高齢者（「5.高齢者への投与」の項参照）

2.重要な基本的注意
1)カルシウム拮抗剤の投与を急に中止したとき、症状が悪化した症例が報告されているので、本剤の休薬を要する場合は徐々に減量し、観察を十分に行うこと。また、患者に医師の指示なしに服薬を中止しないように注意すること。
2)本剤の投与により、まれに過度の血圧低下を起こすおそれがあるので、そのような場合には減量又は休薬するなど適切な処置を行うこと。
3)降圧作用に基づくめまい等があらわれることがあるので、高所作業、自動車の運転等、危険を伴う機械を操作する際には注意させること。

3.相互作用
本剤は、主としてチトクロームP450 3A4（CYP3A4）で代謝される。
1)併用禁忌（併用しないこと）

薬剤名等	臨床症状・措置方法	機序・危険因子
※※ **アゾール系抗真菌剤（外用剤を除く）**<br>・イトラコナゾール（イトリゾール）<br>・ミコナゾール（フロリード）<br>・フルコナゾール（ジフルカン）<br>・ホスフルコナゾール（プロジフ）<br>・ボリコナゾール（ブイフェンド）	イトラコナゾールとの併用により本剤のAUCが2.8倍に上昇することが報告されている。	これらの薬剤がCYP3A4を阻害し、本剤のクリアランスが低下すると考えられる。

※注1）注意－医師等の処方箋により使用すること

薬剤名等	臨床症状・措置方法	機序・危険因子
※※ **HIVプロテアーゼ阻害剤**<br>・リトナビル含有製剤（ノービア、カレトラ）<br>・サキナビル（インビラーゼ）<br>・インジナビル（クリキシバン）<br>・ネルフィナビル（ビラセプト）<br>・アタザナビル（レイアタッツ）<br>・ホスアンプレナビル（レクシヴァ）<br>・ダルナビル含有製剤（プリジスタ、プレジコビックス）<br>**コビシスタット含有製剤**（スタリビルド、ゲンボイヤ、プレジコビックス）	併用により本剤の作用が増強されるおそれがある。	これらの薬剤がCYP3A4を阻害し、本剤のクリアランスが低下すると考えられる。
※※ **オムビタスビル・パリタプレビル・リトナビル**（ヴィキラックス）	併用により本剤の作用が増強されるおそれがある。	リトナビルがCYP3A4を阻害し、本剤のクリアランスが低下すると考えられる。

2）併用注意（併用に注意すること）

薬剤名等	臨床症状・措置方法	機序・危険因子
**他の降圧剤**	過度の降圧が起こるおそれがある。必要があれば他の降圧剤あるいは本剤を減量すること。	作用メカニズムの異なる降圧剤の併用により薬理作用が増強される。
**ジゴキシン**	併用によりジゴキシンのCmaxが1.5倍、AUCが1.3倍に上昇することが報告されている。必要があればジゴキシンを減量すること。	ジゴキシンの腎排泄（尿細管分泌）及び腎外からの排泄を阻害するためと考えられる。
※※ **シメチジン**<br>**イマチニブメシル酸塩**<br>**マクロライド系抗生物質**<br>・エリスロマイシン<br>・クラリスロマイシン<br>等	併用により本剤の作用が増強されるおそれがある。必要があれば本剤を減量あるいはこれらの薬剤の投与を中止すること。	これらの薬剤がCYP3A4を阻害し、本剤のクリアランスが低下すると考えられる。
**シンバスタチン**	併用によりシンバスタチンのAUCが2.0倍に上昇することが報告されている。必要があれば本剤又はシンバスタチンの投与を中止すること。	これらの薬剤がCYP3A4を競合的に阻害することにより、相互のクリアランスが低下すると考えられる。腎機能障害のある患者は特に注意すること。
**シクロスポリン**	併用により本剤又はこれらの薬剤の作用が増強されるおそれがある。必要があれば本剤又はこれらの薬剤を減量すること。	
**ベンゾジアゼピン系薬剤**<br>・ジアゼパム<br>・ミダゾラム<br>・トリアゾラム<br>等<br>**経口黄体・卵胞ホルモン**<br>・経口避妊薬<br>等		これらの薬剤がCYP3A4を競合的に阻害することにより、相互のクリアランスが低下すると考えられる。
**タンドスピロンクエン酸塩**	併用により本剤の作用が増強されるおそれがある。必要があれば本剤を減量あるいはタンドスピロンクエン酸塩の投与を中止すること。	セロトニン受容体を介した中枢性の血圧降下作用が降圧作用を増強する。
**リファンピシン**<br>**フェニトイン**<br>**フェノバルビタール**	併用により本剤の作用が減弱されるおそれがある。	これらの薬剤の代謝酵素誘導作用により、本剤のクリアランスが上昇すると考えられる。
**グレープフルーツジュース**	本剤の血中濃度が上昇することが報告されている。降圧作用が増強されるおそれがあることから、本剤の服用中はグレープフルーツジュースを飲用しないよう注意すること。	グレープフルーツジュースに含まれる成分がCYP3A4による本剤の代謝を阻害し、クリアランスを低下させるためと考えられる。

4．副作用

本剤は、副作用発現頻度が明確となる調査を実施していない。

1）重大な副作用（頻度不明）

（1）肝機能障害、黄疸

AST（GOT）、ALT（GPT）、γ-GTPの上昇等の肝機能障害、黄疸があらわれることがあるので、観察を十分に行い、異常が認められた場合には投与を中止し、適切な処置を行うこと。

（2）房室ブロック、洞停止、徐脈

房室ブロック、洞停止、徐脈があらわれることがあるので、めまい、ふらつき等の異常が認められた場合には投与を中止し、適切な処置を行うこと。

2）その他の副作用

下記の副作用があらわれることがあるので、異常が認められた場合には必要に応じ投与を中止するなど適切な処置を行うこと。

種類＼頻度	頻度不明
**過敏症**[注2）	発疹、そう痒、血管浮腫
**精神神経系**	頭痛・頭重感、ふらつき、めまい、立ちくらみ、眠気
**消化器**	胃部不快感、悪心、便秘、腹痛、下痢、歯肉肥厚、口内炎
**循環器**	動悸、ほてり、顔面潮紅
**血液**	好酸球増多

種類＼頻度	頻度不明
**肝　　臓**	ALT（GPT）上昇、AST（GOT）上昇、LDH上昇、γ-GTP上昇、肝機能異常、ALP上昇、総ビリルビン上昇
**泌　尿　器**	BUN上昇、クレアチニン上昇、尿硝子円柱増加、頻尿
**そ　の　他**	尿酸上昇、総コレステロール上昇、CK（CPK）上昇、カリウム上昇、倦怠感、異常感（浮遊感、気分不良等）、カリウム低下、浮腫、しびれ、乳び腹水[注3)]

注2）投与を中止すること。また、類薬では光線過敏症が報告されている。

注3）低アルブミン血症の患者で起こりやすい。

5.高齢者への投与

高齢者に使用する場合は、８mgあるいは更に低用量から投与を開始し、経過を十分に観察しながら慎重に投与することが望ましい。[一般に高齢者では、過度の降圧は好ましくないとされている（脳梗塞が起こるおそれがある）。]

6.妊婦、産婦、授乳婦等への投与

1)妊婦又は妊娠している可能性のある婦人には投与しないこと。[動物試験（ラット）で妊娠前～初期の投与において着床前及び着床後胚死亡率の増加、出生児の体重低下、妊娠期間及び分娩時間の延長が認められている。また、妊娠末期の投与において妊娠期間及び分娩時間の延長が認められている。]

2)授乳中の婦人への投与は避けることが望ましいが、やむを得ず投与する場合は授乳を中止させること。[動物試験（ラット）で乳汁中へ移行することが報告されている。]

7.小児等への投与

低出生体重児、新生児、乳児、幼児又は小児に対する安全性は確立していない（使用経験がない）。

8.適用上の注意

薬剤交付時

PTP包装の薬剤は、PTPシートから取り出して服用するよう指導すること。[PTPシートの誤飲により、硬い鋭角部が食道粘膜へ刺入し、更には穿孔を起こして縦隔洞炎等の重篤な合併症を併発することが報告されている。]

9.その他の注意

1)因果関係は明らかではないが、本剤による治療中に心筋梗塞、心不全や不整脈（心房細動等）がみられたとの報告がある。

2)CAPD（持続的外来腹膜透析）施行中の患者の透析排液が白濁することが報告されているので、腹膜炎等との鑑別に留意すること。

※【薬 物 動 態】

1.生物学的同等性試験

1)アゼルニジピン錠８mg「NP」

アゼルニジピン錠８mg「NP」と標準製剤のそれぞれ１錠（アゼルニジピンとして８mg）を、クロスオーバー法により健康成人男子に絶食時に経口投与して血漿中アゼルニジピン濃度を測定した。得られた薬物動態パラメータ（$AUC_{0→48hr}$、Cmax）について90％信頼区間法にて統計解析を行った結果、log（0.80)～log（1.25）の範囲内であり、両剤の生物学的同等性が確認された。[1)]

	判定パラメータ		参考パラメータ	
	$AUC_{0→48hr}$ (ng･hr/mL)	Cmax (ng/mL)	Tmax (hr)	$t_{1/2}$ (hr)
アゼルニジピン錠８mg「NP」	40.935±12.250	5.515±2.460	2.67±0.73	9.82±2.49
標準製剤（錠剤、８mg）	37.558±11.166	4.852±1.793	2.81±0.55	9.87±3.45

(Mean±S.D., n＝18)

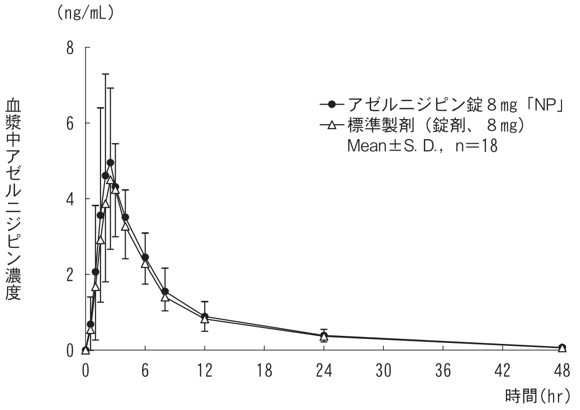

血漿中濃度並びにAUC、Cmax等のパラメータは、被験者の選択、体液の採取回数・時間等の試験条件によって異なる可能性がある。

2)アゼルニジピン錠16mg「NP」

アゼルニジピン錠16mg「NP」と標準製剤のそれぞれ１錠（アゼルニジピンとして16mg）を、クロスオーバー法により健康成人男子に絶食時に経口投与して血漿中アゼルニジピン濃度を測定した。得られた薬物動態パラメータ（$AUC_{0→48hr}$、Cmax）について90％信頼区間法にて統計解析を行った結果、log（0.80)～log（1.25）の範囲内であり、両剤の生物学的同等性が確認された。[2)]

	判定パラメータ		参考パラメータ	
	$AUC_{0→48hr}$ (ng･hr/mL)	Cmax (ng/mL)	Tmax (hr)	$t_{1/2}$ (hr)
アゼルニジピン錠16mg「NP」	91.341±24.391	11.510±4.654	3.15±0.89	10.38±2.69
標準製剤（錠剤、16mg）	84.421±22.292	10.196±4.160	3.05±0.63	10.86±3.19

(Mean±S.D., n＝20)

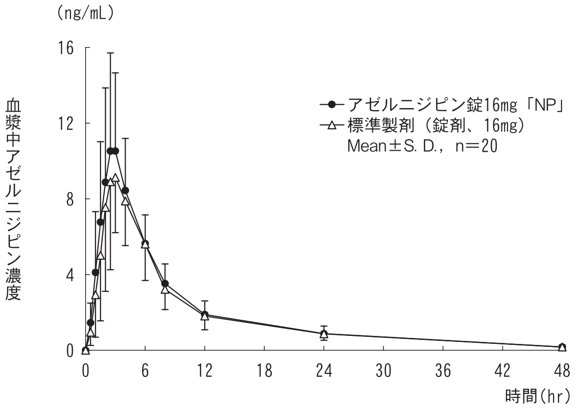

血漿中濃度並びにAUC、Cmax等のパラメータは、被験者の選択、体液の採取回数・時間等の試験条件によって異なる可能性がある。

※2.溶出挙動

アゼルニジピン錠８mg「NP」及びアゼルニジピン錠16mg「NP」は、日本薬局方医薬品各条に定められたアゼルニジピン錠の溶出規格に適合していることが確認されている。[3)]

【薬 効 薬 理】

ジヒドロピリジン系Ca拮抗薬。膜電位依存性L型カルシウムチャネルに特異的に結合し、細胞内へのカルシウムの流入を減少させることにより、冠血管や末梢血管の平滑筋を弛緩させる。なお、本薬は作用の持続性が特徴とされる。[4)]

【有効成分に関する理化学的知見】

一般名：アゼルニジピン（Azelnidipine）
化学名：3-[1-(Diphenylmethyl)azetidin-3-yl]5-(1-methylethyl)(4*RS*)-2-amino-6-methyl-4-(3-nitrophenyl)-1,4-dihydropyridine-3,5-dicarboxylate
分子式：$C_{33}H_{34}N_4O_6$
分子量：582.65
構造式：

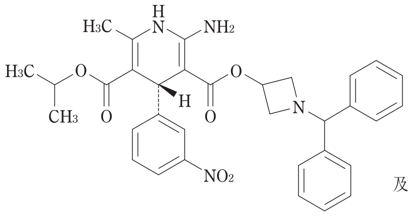

及び鏡像異性体

性　状：・淡黄色～黄色の結晶性の粉末又は塊を含む粉末である。
・エタノール（99.5）又は酢酸（100）に溶けやすく、水にほとんど溶けない。
・エタノール（99.5）溶液（１→100）は旋光性を示さない。
・結晶多形が認められる。

【取扱い上の注意】

1.開封後の注意

本剤は光により着色するので開封後も遮光して保存すること。

2.安定性試験

1)アゼルニジピン錠８mg「NP」

最終包装製品を用いた加速試験（40℃、相対湿度75％、６カ月）の結果、アゼルニジピン錠８mg「NP」は通常の市場流通下において３年間安定であることが推測された。[5)]

2)アゼルニジピン錠16mg「NP」

最終包装製品を用いた加速試験（40℃、相対湿度75％、６カ月）の結果、アゼルニジピン錠16mg「NP」は通常の市場流通下において３年間安定であることが推測された。[6)]

【包　　装】

アゼルニジピン錠８mg「NP」：100錠（PTP）
500錠（PTP）
アゼルニジピン錠16mg「NP」：100錠（PTP）
140錠（PTP）
500錠（PTP）
700錠（PTP）

※※※【主 要 文 献】

1)ニプロ（株）：社内資料（生物学的同等性試験）
2)ニプロ（株）：社内資料（生物学的同等性試験）
※3)ニプロ（株）：社内資料（溶出試験）
※※4)第十七改正日本薬局方解説書
5)ニプロ（株）：社内資料（安定性試験）
6)ニプロ（株）：社内資料（安定性試験）

【文献請求先・製品情報お問い合わせ先】

主要文献欄に記載の社内資料につきましても下記にご請求ください。

ニプロ株式会社　医薬品情報室
〒531-8510 大阪市北区本庄西３丁目９番３号
0120-226-898
FAX 06-6375-0177

NIPRO
製造販売　ニプロ株式会社
大阪市北区本庄西3丁目9番3号

C－1

※※印：2016年10月改訂（第17版、社名変更等に伴う改訂）
※印：2014年 6月改訂

日本標準商品分類番号	872144

貯　　法：室温・気密容器保存
使用期限：外装に表示の使用期限内に使用すること。
※ 規制区分：処方箋医薬品
（注意－医師等の処方箋により使用すること）

	12.5mg	25mg	50mg
承認番号	22300AMX00062000	22100AMX02344000	22300AMX01130000
薬価収載	2011年11月	2011年11月	2011年11月
販売開始	2011年11月	1999年 7月	2011年11月
再評価結果（品質再評価）	―	2001年 4月	―

日本薬局方
アラセプリル錠
持続性ACE阻害降圧剤
アラセプリル錠12.5mg「タイヨー」
アラセプリル錠25mg「タイヨー」
アラセプリル錠50mg「タイヨー」
ALACEPRIL

【禁忌（次の患者には投与しないこと）】
(1) 本剤の成分に対し過敏症の既往歴のある患者
(2) 血管浮腫の既往歴のある患者（アンジオテンシン変換酵素阻害剤等の薬剤による血管浮腫、遺伝性血管浮腫、後天性血管浮腫、特発性血管浮腫等）［高度の呼吸困難を伴う血管浮腫を発現することがある］
(3) デキストラン硫酸固定化セルロース、トリプトファン固定化ポリビニルアルコールまたはポリエチレンテレフタレートを用いた吸着器によるアフェレーシスを施行中の患者［ショックを起こすことがある］（「**相互作用**」の項参照）
(4) アクリロニトリルメタリルスルホン酸ナトリウム膜（AN69®）を用いた血液透析施行中の患者［アナフィラキシーが発現することがある］（「**相互作用**」の項参照）
(5) 妊婦または妊娠している可能性のある婦人（「**妊婦、産婦、授乳婦等への投与**」の項参照）
(6) アリスキレンを投与中の糖尿病患者（ただし、他の降圧治療を行ってもなお血圧のコントロールが著しく不良の患者を除く）［非致死性脳卒中、腎機能障害、高カリウム血症及び低血圧のリスク増加が報告されている］（「**重要な基本的注意**」の項参照）

【組成・性状】

	アラセプリル錠12.5mg「タイヨー」	アラセプリル錠25mg「タイヨー」	アラセプリル錠50mg「タイヨー」
組　成	1錠中：アラセプリル……12.5mg〈添加物〉結晶セルロース、ショ糖脂肪酸エステル、ステアリン酸マグネシウム、低置換度ヒドロキシプロピルセルロース	1錠中：アラセプリル……25mg〈添加物〉軽質無水ケイ酸、結晶セルロース、ステアリン酸マグネシウム、低置換度ヒドロキシプロピルセルロース、バレイショデンプン、ヒドロキシプロピルセルロース	1錠中：アラセプリル……50mg〈添加物〉結晶セルロース、ステアリン酸マグネシウム、低置換度ヒドロキシプロピルセルロース、ヒドロキシプロピルセルロース
性　状	白色の片面1/2割線入り素錠		
識別コード（PTP）	t AC 12.5mg	t AC25 25mg	t AC50 50mg

		アラセプリル錠12.5mg「タイヨー」		アラセプリル錠25mg「タイヨー」		アラセプリル錠50mg「タイヨー」	
外形（サイズ）	表（直径mm）	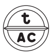	6.0	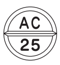	7.0	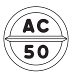	8.5
	裏（重量mg）	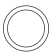	80	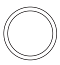	105	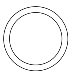	200
	側面（厚さmm）	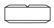	2.2	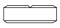	2.3	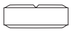	3.0

【効能・効果】
本態性高血圧症、腎性高血圧症

translation: 25 - 75 mg/ day

【用法・用量】
通常、成人にアラセプリルとして1日25～75mgを1～2回に分割経口投与する。年齢、症状により適宜増減する。
なお、重症例においても1日最大投与量は100mgまでとする。

〈用法・用量に関連する使用上の注意〉
重篤な腎機能障害のある患者では、活性代謝物の血中濃度が上昇し、過度の血圧低下、腎機能の悪化が起こるおそれがあるので、血清クレアチニン値が3mg/dLを超える場合には、投与量を減らすかまたは投与間隔を延ばすなど慎重に投与すること。

【使用上の注意】
1. 慎重投与（次の患者には慎重に投与すること）
(1) 両側性腎動脈狭窄のある患者または片腎で腎動脈狭窄のある患者（「**重要な基本的注意**」の項参照）
(2) 高カリウム血症の患者（「**重要な基本的注意**」の項参照）
(3) 重篤な腎機能障害のある患者（**〈用法・用量に関連する使用上の注意〉**の項参照）
(4) 高齢者（「**高齢者への投与**」の項参照）

2. 重要な基本的注意
(1) **両側性腎動脈狭窄のある患者**または**片腎で腎動脈狭窄のある患者**においては、腎血流量の減少や糸球体ろ過圧の低下により急速に腎機能を悪化させるおそれがあるので、**治療上やむを得ないと判断される場合を除き、使用は避けること。**
(2) **高カリウム血症の患者**においては、高カリウム血症を増悪させるおそれがあるので、**治療上やむを得ないと判断される場合を除き、使用は避けること。**
また、腎機能障害、コントロール不良の糖尿病等により血清カリウム値が高くなりやすい患者では、高カリウム血症が発現するおそれがあるので、血清カリウム値に注意すること。

＜裏面につづく＞

(3) アリスキレンを併用する場合、腎機能障害、高カリウム血症及び低血圧を起こすおそれがあるため、患者の状態を観察しながら慎重に投与すること。なお、eGFRが60mL/min/1.73m^{2}未満の腎機能障害のある患者へのアリスキレンとの併用については、治療上やむを得ないと判断される場合を除き避けること。
(4) **腎機能障害のある患者**および**腎疾患の既往歴のある患者**に投与する場合には、投与は**少量かつ1日1回投与より開始**し、増量を必要とする場合は、患者の状態を十分に観察しながら徐々に行うこと。
(5) 本剤の投与により次の患者では、**初回投与後一過性の急激な血圧低下**を起こす場合があるので、投与は少量より開始し、増量する場合は、患者の状態を十分に観察しながら徐々に行うこと。
1) 重症の高血圧症患者
2) 血液透析中の患者
3) 利尿降圧剤投与中の患者（特に最近利尿降圧剤投与を開始した患者）
4) 厳重な減塩療法中の患者
(6) 降圧作用に基づくめまい、ふらつきがあらわれることがあるので、高所作業、自動車の運転など危険を伴う機械を操作する際には注意させること。
(7) 手術前24時間は投与しないことが望ましい。

3. 相互作用

(1) **併用禁忌**（併用しないこと）

薬剤名等	臨床症状・措置方法	機序・危険因子
デキストラン硫酸固定化セルロース（リポソーバー®、セレソーブ®）、トリプトファン固定化ポリビニルアルコール（イムソーバTR®）またはポリエチレンテレフタレート（セルソーバ®）を用いた吸着器によるアフェレーシスの施行	ショックを起こすことがある。	陰性に荷電したデキストラン硫酸固定化セルロース、トリプトファン固定化ポリビニルアルコールまたはポリエチレンテレフタレートにより血中キニン系の代謝が亢進し、本剤によりブラジキニンの代謝が妨げられ蓄積すると考えられている。
アクリロニトリルメタリルスルホン酸ナトリウム膜を用いた透析 AN69®	アナフィラキシーを発現することがある。	多価イオン体であるAN69®により血中キニン系の代謝が亢進し、本剤によりブラジキニンの代謝が妨げられ蓄積すると考えられている。

(2) **併用注意**（併用に注意すること）

薬剤名等	臨床症状・措置方法	機序・危険因子
カリウム保持性利尿剤 スピロノラクトン トリアムテレン 等 カリウム補給剤 塩化カリウム 等	血清カリウム値が上昇することがある。	本剤によりアンジオテンシンⅡが低下してアルドステロンの分泌減少をきたし、カリウム排泄量が少なくなる。特に腎機能障害のある患者では注意すること。
アリスキレン	腎機能障害、高カリウム血症及び低血圧を起こすおそれがあるため、腎機能、血清カリウム値及び血圧を十分に観察すること。なお、eGFRが60mL/min/1.73m^{2}未満の腎機能障害のある患者へのアリスキレンとの併用については、治療上やむを得ないと判断される場合を除き避けること。	併用によりレニン・アンジオテンシン系阻害作用が増強される可能性がある。
※アンジオテンシンⅡ受容体拮抗剤	腎機能障害、高カリウム血症及び低血圧を起こすおそれがあるため、腎機能、血清カリウム値及び血圧を十分に観察すること。	併用によりレニン・アンジオテンシン系阻害作用が増強される可能性がある。
カリジノゲナーゼ製剤	本剤との併用により過度の血圧低下が引き起こされる可能性がある。	カリジノゲナーゼによるキニン産生作用とアンジオテンシン変換酵素阻害剤のキニン分解抑制作用により、キニン系が亢進し、血管平滑筋の弛緩が増強されると考えられる。
利尿降圧剤 チアジド系利尿剤 ヒドロクロロチアジド 等 ループ利尿剤 等 減塩療法 血液透析の治療	本剤を初めて併用する場合、降圧作用が増強するおそれがあるので、減量するなど慎重に投与すること。	利尿降圧剤の投与中や減塩療法、血液透析の治療中には血漿レニン活性が上昇しており、本剤の投与により急激な血圧低下をきたす。
リチウム	他のアンジオテンシン変換酵素阻害剤（カプトプリル、エナラプリル、リシノプリル）との併用により、リチウム中毒が報告されているので、本剤においても血中のリチウム濃度に注意すること。	リチウムとナトリウムは近位尿細管で競合的に再吸収されており、本剤によるナトリウム排泄作用によりリチウムの再吸収が促進される。
アロプリノール	過敏症状（Stevens-Johnson症候群、関節痛等）が発現したとの報告がある。患者の状態を注意深く観察し、発熱を伴う発疹等の過敏症状が発現した場合には直ちに両剤の投与を中止すること。	機序は不明である。特に腎機能障害のある患者では注意すること。
非ステロイド性消炎鎮痛剤 インドメタシン等	本剤の降圧作用が減弱することがある。	非ステロイド性消炎鎮痛剤のプロスタグランジン合成阻害作用により、本剤のプロスタグランジンを介した降圧作用が減弱される。
	腎機能が低下している患者では、さらに腎機能が悪化するおそれがある。	プロスタグランジン合成阻害作用により、腎血流量が低下するためと考えられる。

4. 副作用

本剤は使用成績調査等の副作用発現頻度が明確となる調査を実施していない。

(1) **重大な副作用**（頻度不明）

1) **血管浮腫**　呼吸困難を伴う顔面、舌、声門、喉頭の腫脹を症状とする血管浮腫があらわれることがあるので、このような場合には、直ちに投与を中止し、気道の確保など適切な処置を行うこと。
2) **無顆粒球症**　無顆粒球症があらわれることがあるので、観察を十分に行い、異常が認められた場合には、投与を中止するなど適切な処置を行うこと。
3) **天疱瘡様症状**　天疱瘡様症状があらわれることがあるので、このような場合には、減量または投与を中止するなど適切な処置を行うこと。
4) **高カリウム血症**　重篤な高カリウム血症があらわれることがあるので、観察を十分に行い、異常が認められた場合には、直ちに適切な処置を行うこと。

⑵ **類薬による重大な副作用**
汎血球減少、急性腎不全、膵炎　他のアンジオテンシン変換酵素阻害剤（カプトプリルまたはエナラプリル）で、これらの副作用が報告されているので、観察を十分に行い、異常が認められた場合には、投与を中止するなど適切な処置を行うこと。

⑶ **その他の副作用**

	頻　度　不　明
腎臓注1)	BUN、クレアチニンの上昇、蛋白尿
血液注1)	白血球減少、貧血、血小板減少、好酸球増多
過敏症注1)	発疹、そう痒感
循環器	起立性低血圧、胸部不快感、動悸
呼吸器	咳嗽、咽喉頭異物感、喀痰増加
精神神経系	めまい、ふらつき感、頭痛、頭重、眠気、浮遊感、四肢しびれ感、口内しびれ感
消化器	悪心、下痢、食欲不振、胃部不快感、胸やけ、口渇、口内炎
味覚注2)	味覚異常
肝臓	ALT（GPT）、AST（GOT）、γ-GTP、Al-Pの上昇、黄疸
その他	全身倦怠感、浮腫、顔面のほてり、血清カリウム値の上昇、抗核抗体の陽性例、低血糖注3)

注1）観察を十分に行い、異常が認められた場合には、減量または投与を中止するなど適切な処置を行うこと。
注2）このような場合には、減量または投与を中止するなど適切な処置を行うこと（通常、味覚異常は可逆的である）。
注3）「**その他の注意**」の項参照

5. 高齢者への投与
低用量から投与を開始するなど患者の状態を観察しながら慎重に投与すること。［高齢者では一般に過度の降圧は好ましくないとされている（脳梗塞等が起こるおそれがある）］

6. 妊婦、産婦、授乳婦等への投与
⑴ 妊婦または妊娠している可能性のある婦人には投与しないこと。また、投与中に妊娠が判明した場合には、直ちに投与を中止すること。［妊娠中期および末期にアンジオテンシン変換酵素阻害剤を投与された高血圧症の患者で羊水過少症、胎児・新生児の死亡、新生児の低血圧、腎不全、高カリウム血症、頭蓋の形成不全および羊水過少症によると推測される四肢の拘縮、頭蓋顔面の変形等があらわれたとの報告がある。また、海外で実施されたレトロスペクティブな疫学調査で、妊娠初期にアンジオテンシン変換酵素阻害剤を投与された患者群において、胎児奇形の相対リスクは降圧剤が投与されていない患者群に比べ高かったとの報告がある。］
⑵ 授乳中の婦人に投与することを避け、やむを得ず投与する場合には、授乳を中止させること。［動物実験（ラット）で乳汁中へ移行することが認められている］

7. 小児等への投与
小児等に対する安全性は確立していない（使用経験がない）。

8. 臨床検査結果に及ぼす影響
尿中ケトン（アセトン）が偽陽性を呈することがある。

9. 適用上の注意
薬剤交付時：PTP包装の薬剤はPTPシートから取り出して服用するよう指導すること。（PTPシートの誤飲により、硬い鋭角部が食道粘膜へ刺入し、更には穿孔をおこして縦隔洞炎等の重篤な合併症を併発することが報告されている）

10. その他の注意
インスリンまたは経口血糖降下剤の投与中にアンジオテンシン変換酵素阻害剤を投与することにより、低血糖が起こりやすいとの報告がある。

【 薬 物 動 態 】

1. 生物学的同等性試験[1)]
●アラセプリル錠12.5mg「タイヨー」
アラセプリル錠12.5mg「タイヨー」と標準製剤を、クロスオーバー法によりそれぞれ2錠（アラセプリルとして25mg）健康成人男子に絶食単回経口投与して血漿中遊離型カプトプリル濃度及び血漿中総カプトプリル濃度を測定し、得られた薬物動態パラメータ（AUC、Cmax）について統計解析を行った結果、両剤の生物学的同等性が確認された。

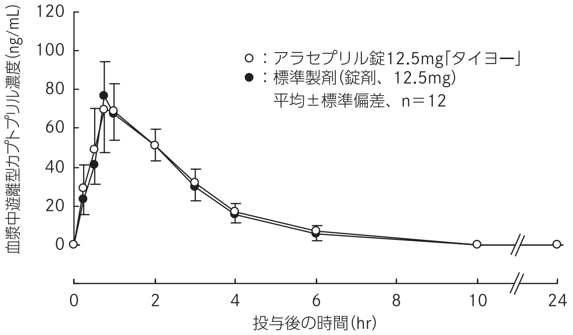

薬物動態パラメータ（遊離型カプトプリル）（平均±標準偏差、n＝12）

	投与量（mg）	AUC0-24（ng・hr/mL）	Cmax（ng/mL）	Tmax（hr）	T1/2（hr）
アラセプリル錠12.5mg「タイヨー」	25	207.62 ±33.86	79.08 ±15.20	0.85 ±0.13	1.45 ±0.27
標準製剤（錠剤、12.5mg）	25	198.13 ±34.52	78.56 ±14.36	0.79 ±0.10	1.35 ±0.29

血漿中濃度並びにAUC、Cmax等のパラメータは、被験者の選択、体液の採取回数・時間等の試験条件によって異なる可能性がある。

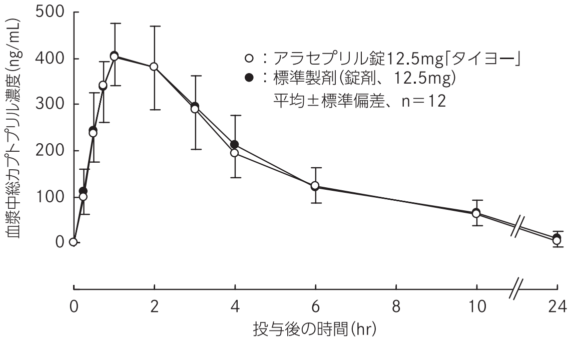

薬物動態パラメータ（総カプトプリル）（平均±標準偏差、n＝12）

	投与量（mg）	AUC0-24（ng・hr/mL）	Cmax（ng/mL）	Tmax（hr）	T1/2（hr）
アラセプリル錠12.5mg「タイヨー」	25	2366.6 ±505.5	447.7 ±43.2	1.35 ±0.58	4.82 ±2.65
標準製剤（錠剤、12.5mg）	25	2453.1 ±489.3	438.6 ±74.8	1.40 ±0.54	5.67 ±4.26

血漿中濃度並びにAUC、Cmax等のパラメータは、被験者の選択、体液の採取回数・時間等の試験条件によって異なる可能性がある。

<裏面につづく>

●アラセプリル錠25mg「タイヨー」

アラセプリル錠25mg「タイヨー」と標準製剤を、クロスオーバー法によりそれぞれ1錠（アラセプリルとして25mg）健康成人男子に絶食単回経口投与して血漿中遊離型カプトプリル濃度を測定し、得られた薬物動態パラメータ（AUC、Cmax）について統計解析を行った結果、両剤の生物学的同等性が確認された。

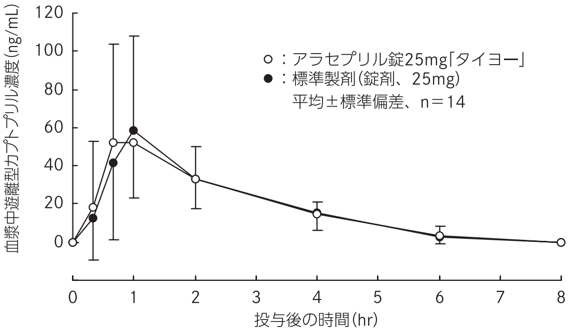

薬物動態パラメータ　　　　　（平均±標準偏差、n＝14）

	投与量 (mg)	AUC_{0-8} (ng・hr/mL)	Cmax (ng/mL)	Tmax (hr)	$T_{1/2}$ (hr)
アラセプリル錠25mg「タイヨー」	25	143.2 ±60.5	66.5 ±44.3	0.9 ±0.3	2.6 ±2.2
標準製剤（錠剤、25mg）	25	142.2 ±67.1	65.0 ±45.4	1.5 ±0.5	2.2 ±1.2

血漿中濃度並びにAUC、Cmax等のパラメータは、被験者の選択、体液の採取回数・時間等の試験条件によって異なる可能性がある。

2. 溶出性[2)]

アラセプリル錠12.5mg「タイヨー」、アラセプリル錠25mg「タイヨー」及びアラセプリル錠50mg「タイヨー」の溶出性は、日本薬局方に定められた規格に適合していることが確認されている。

【薬効薬理】[3)]

アラセプリルはアンジオテンシン変換酵素阻害薬である。経口投与後、デアセチルアラセプリルとカプトプリルに代謝されて作用を発現し、生理活性を持たないアンジオテンシンⅠから強力な昇圧活性を有するアンジオテンシンⅡへの変換を阻害することにより、血圧降下作用を示す。なお、アンジオテンシン変換酵素はキニナーゼⅡと同一の酵素であり、変換酵素阻害薬は血管拡張物質ブラジキニンの分解を抑制する。

【有効成分に関する理化学的知見】

一般名：アラセプリル（Alacepril）

化学名：(2*S*)-2-{(2*S*)-1-[(2*S*)-3-(Acetylsulfanyl)-2-methylpropanoyl]pyrrolidine-2-carbonyl}amino-3-phenylpropanoic acid

分子式：$C_{20}H_{26}N_2O_5S$

分子量：406.50

融　点：153～157℃

性　状：アラセプリルは白色の結晶又は結晶性の粉末である。メタノールに溶けやすく、エタノール(95)にやや溶けやすく、水に溶けにくい。水酸化ナトリウム試液に溶ける。

構造式：

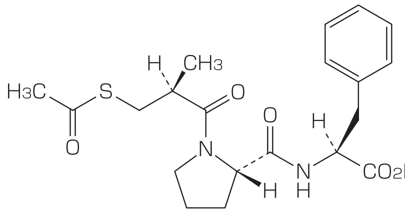

【取扱い上の注意】[4)]

安定性試験結果の概要

●アラセプリル錠12.5mg「タイヨー」

長期保存試験（3年）の結果、アラセプリル錠12.5mg「タイヨー」は通常の市場流通下において3年間安定であることが確認された。

●アラセプリル錠25mg「タイヨー」

●アラセプリル錠50mg「タイヨー」

加速試験（40℃、相対湿度75%、6ヵ月）の結果、アラセプリル錠25mg「タイヨー」及びアラセプリル錠50mg「タイヨー」は通常の市場流通下において3年間安定であることが推測された。

【包　装】

●アラセプリル錠12.5mg「タイヨー」

PTP包装：100錠（10錠×10）

●アラセプリル錠25mg「タイヨー」

PTP包装：100錠（10錠×10）、1,200錠（10錠×120）

●アラセプリル錠50mg「タイヨー」

PTP包装：100錠（10錠×10）

【主要文献】

1) 武田テバファーマ㈱社内資料（生物学的同等性試験）
2) 武田テバファーマ㈱社内資料（溶出試験）
3) 第十六改正日本薬局方解説書
4) 武田テバファーマ㈱社内資料（安定性試験）

※※【文献請求先・製品情報お問い合わせ先】

主要文献欄に記載の文献・社内資料は下記にご請求下さい。

武田テバファーマ株式会社　武田テバDIセンター
〒453-0801　名古屋市中村区太閤一丁目24番11号
TEL　0120-923-093
受付時間　9:00～17:30（土日祝日・弊社休業日を除く）

※※販売
武田薬品工業株式会社
大阪市中央区道修町四丁目1番1号

※※製造販売元
武田テバファーマ株式会社
名古屋市中村区太閤一丁目24番11号

PQM19601
PQU64601
PQU68401

003012−24
2015年７月改訂（第7版、処方箋医薬品表示変更に伴う改訂）
2009年６月改訂

日本標準商品分類番号
872149

translation: guanabenz
(name of agent)

劇薬
処方箋医薬品注）

高血圧症治療剤

ワイテンス®錠2mg

（グアナベンズ酢酸塩錠）

WYTENS® Tablets 2mg

承認番号	22000AMX00641000
薬価収載	2008年 6月
販売開始	1985年 12月
再審査結果	1993年 9月

注）注意－医師等の処方箋により使用すること

貯　　法：遮光・気密容器、室温保存
使用期限：外箱等に表示

【禁忌（次の患者には投与しないこと）】
本剤の成分に対し過敏症の既往歴のある患者

【組成・性状】

	ワイテンス錠2mg		
成分･含量	1錠中「日本薬局方」グアナベンズ酢酸塩2.525mg（グアナベンズとして2mg）		
添加物	結晶セルロース、乳糖水和物、ステアリン酸マグネシウム		
性　状	白色の割線入りの素錠		
外　形	表	裏	側　面
	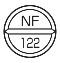	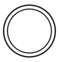	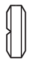
大きさ	直径：6.5mm		厚さ：2.3mm
質　量	100mg		
識別コード	NF　122		

【効能・効果】
本態性高血圧症

translation: 2 - 4 mg/day

【用法・用量】
グアナベンズとして、通常成人1回2mg1日2回経口投与する。
効果が不十分な場合は、1回4mg1日2回に増量する。
なお、年齢、症状に応じて適宜増減する。

【使用上の注意】
1. 慎重投与（次の患者には慎重に投与すること）
(1)肝障害のある患者［肝初回通過効果を受けにくくなり、高い血中濃度が持続するおそれがある。］
(2)腎障害のある患者［排泄遅延により、高い血中濃度が持続するおそれがある。］
(3)狭心症、心筋梗塞の患者［動物実験で心拍数減少と心収縮力低下が認められているので、症状が悪化するおそれがある。］
(4)高血圧以外の原因による心不全のある患者［動物実験で心拍数減少と心収縮力低下が認められているので、症状が悪化するおそれがある。］
(5)虚血性心疾患およびうっ血性心不全の既往歴のある患者［動物実験で心拍数減少と心収縮力低下が認められているので、症状が悪化するおそれがある。］
(6)脳血管障害のある患者［血圧下降に基づく脳血流量の低下により、脳梗塞を惹起するおそれがある。］
(7)高齢者（「5. 高齢者への投与」の項参照）
2. 重要な基本的注意
(1)眠気、めまい、ふらつき等があらわれることがあるので、高所作業、自動車の運転等危険を伴う作業には注意させること。
(2)類似化合物（クロニジン）を投与している患者で急に投与を中止すると、まれに血圧の上昇、神経過敏、頻脈、不安感、頭痛等のリバウンド現象があらわれることが知られているので、本剤の使用にあたっても、投与を中止しなければならない場合には、高血圧治療で一般に行われているように、投与量を徐々に減らすこと。

3. 相互作用
［併用注意］（併用に注意すること）

薬剤名等	臨床症状・措置方法	機序・危険因子
中枢神経抑制薬<br>バルビタール、チオペンタールナトリウム、モルヒネ塩酸塩水和物、ブロチゾラム、ジアゼパム等	相互に作用が増強されることがある。<br>患者の状態を注意深く観察し、眠気、めまい、ふらつき等の症状が認められた場合、本剤又は中枢神経抑制薬を減量若しくは中止するなど適切な処置を行う。	本剤は一般薬理試験で各種の中枢抑制作用を示しまたチオペンタールによる睡眠時間の延長作用が認められているため、薬力学的な相加・相乗作用によるものと考えられている。
アルコール	相互に作用が増強されることがある。<br>患者の状態を注意深く観察し、眠気、めまい、ふらつき等の症状が認められた場合、本剤を減量又はアルコールを中止するなど適切な処置を行う。	本剤は一般薬理試験で各種の中枢抑制作用を示しまたチオペンタールによる睡眠時間の延長作用が認められているため、薬力学的な相加・相乗作用によるものと考えられている。
β遮断剤<br>プロプラノロール塩酸塩、カルテオロール塩酸塩等	本剤投与中止後のリバウンド現象が強められるおそれがあるので、投与を中止する場合にはβ遮断剤を先に中止し、数日間経過を観察した後、本剤の投与を中止する。<br>また、患者に医師の指示なしに使用を中止しないように注意すること。	本剤は交感神経終末からのノルアドレナリン遊離を抑制する。このため、本剤を先に中止した場合神経終末からのノルアドレナリン遊離が増加し、α_1刺激効果（血管収縮等）が発現する。他方、β遮断作用は残っているため、β刺激効果（血管拡張作用等）は発現しないことから、過度の血圧上昇等が発現するおそれがあると考えられている。
抗うつ剤<br>セチプチリンマレイン酸塩、ミアンセリン塩酸塩等	本剤の降圧効果が減弱するおそれがある。<br>患者の状態を注意深く観察し、血圧上昇等の症状が認められた場合、他剤への変更、又は抗うつ剤の投与を中止するなど適切な処置を行う。	動物実験（モルモット摘出回腸）で、類似化合物（クロニジン）のα_2刺激作用が抗うつ剤セチプチリンマレイン酸塩により競合的に拮抗されることが報告されており、本剤でも同様のことが生じると考えられている。
降圧作用を有する薬剤	降圧作用が増強されることがある。	共に降圧作用を有するため。

4. 副作用
調査症例15,358例中822例（5.4%）に副作用が報告されている。その主なものは口渇等の消化器症状2.9%、眠気、めまい等の精神神経系症状2.8%、発疹等の過敏症状0.2%である。（再審査終了時）

	0.1〜5%未満	0.1%未満
過敏症注）	発疹	顔面湿疹、蕁麻疹、瘙痒
肝　臓	———	AST（GOT）、ALT（GPT）の上昇
精神神経系	眠気、めまい、ふらつき、立ちくらみ、倦怠感、脱力感、頭痛・頭重	耳鳴、不眠、ゆううつ感、振せん
循環器	———	動悸、胸痛、徐脈、不整脈、過度の降圧
消化器	口渇、腹部不快感、悪心	食欲不振、下痢、便秘、嘔吐、胸やけ、苦味感、胃痛

	0.1～5%未満	0.1%未満
その他	―――	肩こり、腰痛、筋肉痛、しびれ感、四肢冷感、鼻閉、呼吸困難、顔面潮紅、浮腫、膀胱テネスムス、頻尿

注）発現した場合には、投与を中止すること。

5. 高齢者への投与
高齢者では低用量から投与を開始するなど患者の状態を観察しながら慎重に投与すること。[一般に過度の降圧は好ましくないとされている（脳梗塞等が起こるおそれがある）。]

6. 妊婦、産婦、授乳婦等への投与
(1)妊婦又は妊娠している可能性のある婦人には、治療上の有益性が危険性を上回ると判断される場合にのみ投与すること。[動物実験（ラット）で、妊娠早期に胚胎児の吸収が報告されている。]
(2)授乳婦への投与は避けることが望ましいが、やむを得ず投与する場合は授乳を避けさせること。［動物実験（ラット）で乳汁中への移行が報告されている。]

7. 小児等への投与
小児等に関する安全性は確立していない。

8. 過量投与
症状：副作用症状（低血圧、傾眠、嗜眠、過敏症、徐脈等）が強くあらわれる。また、縮瞳があらわれることがある。
処置：胃洗浄及び経口活性炭、昇圧薬、輸液の投与が有効な場合がある。

9. 適用上の注意
薬剤交付時：
PTP包装の薬剤はPTPシートから取り出して服用するよう指導すること。[PTPシートの誤飲により、硬い鋭角部が食道粘膜へ刺入し、更には穿孔をおこして縦隔洞炎等の重篤な合併症を併発することが報告されている。]

10. その他の注意
小児喘息の既往を有する高血圧患者で、本剤の投与により喘鳴がみられたという報告がある。

【薬 物 動 態】

1. 血中濃度[1)]
健常成人男子にグアナベンズを1回8mg経口投与した場合、血漿中濃度は投与後2時間で最高値（2.14ng/mL）に達し、血漿中半減期は約5.4時間である。

2. 代謝・排泄[1)]
尿中累積排泄率は投与後48時間までにほぼプラトーに達し、投与量の約41%である。尿中代謝物は主として4-ヒドロキシグアナベンズ及びその抱合体であり、未変化体はわずかである。
（参考）[2)]
ラット経口投与による吸収は良好かつ速やかである。その主たる吸収部位は小腸である。27%の消化管再吸収があり、腸肝循環の関与が考えられる。
経口投与後の組織内濃度は消化管及び肝で高く、次いで膵、腎、肺、脾、血液及び血漿でやや高く、中枢神経系及び眼球では低い。
未変化体は血液－脳関門を容易に通過する。
投与後24時間までに投与量の約90%が糞便及び尿中に排泄される。

【臨 床 成 績】

1. 本態性高血圧症に対する効果
387例について実施された一般臨床試験[3~6)]において、378例中215例（56.9%）に降圧効果が認められている。また、二重盲検試験により本剤の有用性が認められている。

2. 血圧の日内変動に及ぼす影響[7)]
本剤1回2又は4mg1日2回（朝夕）投与例において、血圧の日内変動に影響を及ぼすことなく、血圧を下降させることが認められている。

3. 長期投与例における降圧効果[8)]
1年以上の長期投与例において、血圧のコントロールは良好であることが認められている。

【薬 効 薬 理】

〈降 圧 作 用〉
1. 選択的α_2－アドレナリン受容体刺激作用を有する（ラット摘出輸精管[9)]、ウサギ摘出大動脈条片[10)]）。
2. 中枢部位に作用して遠心性交感神経活動を低下させるとともに交感神経終末における神経伝達を遮断することにより血圧を低下させる（麻酔ネコ[11)]：静脈内投与、ウサギ摘出標本[12)]）。
3. 高血圧自然発症ラット[13)]、腎性高血圧ラット[13)]及びイヌ[14)]、DOCA－Salineラット[13)]において、経口投与によりいずれもほぼ同程度の持続性降圧作用を有する。

【有効成分に関する理化学的知見】

一般名：グアナベンズ酢酸塩（Guanabenz Acetate）
化学名：(*E*)-1-(2, 6-Dichlorobenzylideneamino) guanidine monoacetate
構造式：

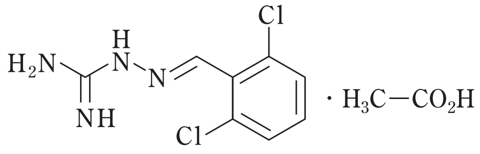

分子式：$C_8H_8Cl_2N_4 \cdot C_2H_4O_2$
分子量：291. 13
性　状：白色の結晶又は結晶性の粉末である。
酢酸(100)に溶けやすく、メタノール又はエタノール(95)にやや溶けやすく、水に溶けにくく、ジエチルエーテルにほとんど溶けない。
光によって徐々に変化する。
融　点：約190℃（分解）
分配係数

pH	酢酸エチル／緩衝液	クロロホルム／緩衝液
3.0	0.07	0
5.0	0.09	0.02
6.0	0.56	0.08
7.0	3.97	0.52
8.0	35.06	3.25
9.0	―	8.07
11.0	―	13.22

【包　　装】

100錠、500錠、1000錠（PTP）
1000錠（バラ）

【主 要 文 献】

1)中島光好　他：臨床薬理, 14, 637(1983)
2)横山信治　他：医薬品研究, 13, 1190(1982)
3)石山太朗　他：臨牀と研究, 60, 649(1983)
4)五島雄一郎　他：薬理と治療, 10, 4657(1982)
5)尾前照雄　他：臨牀と研究, 60, 3687(1983)
6)五島雄一郎　他：薬理と治療, 10, 4675(1982)
7)金子好宏　他：循環器科, 16, 85(1984)
8)河村　博　他：現代の診療, 24, 1167(1982)
9)MacDonald, A. et al.：Br J Pharmacol, 71, 445(1980)
10)Sakakibara, Y. et al.：Jpn J Pharmacol, 31, 1029(1981)
11)Baum, T. et al.：Eur J Pharmacol, 37, 31(1976)
12)Misu, Y. et al.：Eur J Pharmacol, 77, 177(1982)
13)Misu, Y. et al.：応用薬理, 24, 245(1982)
14)Baum, T. et al.：Experientia, 25, 1066(1969)

【文献請求先】

アルフレッサ ファーマ株式会社　学術情報部
〒540-8575 大阪市中央区石町二丁目２番９号
TEL 06-6941-0306　 FAX 06-6943-8212

alfresa
製造販売元　アルフレッサ ファーマ株式会社
大阪市中央区石町二丁目２番９号

®登録商標

Supplementary Data 2 [package inserts of azelnidipine, alacepril, bunazosin, and guanabenz]

**2018年４月改訂（第13版）
*2016年６月改訂

処方箋医薬品注）

日本標準商品分類番号
872149

１日１回型α_1遮断降圧剤

デタントール®R錠3mg
デタントール®R錠6mg　Detantol®R

〈ブナゾシン塩酸塩徐放性製剤〉

translation: bunazosin

	錠3mg	錠6mg
承認番号	20700AMZ00013000	20700AMZ00014000
薬価収載	1995年３月	1995年３月
販売開始	1995年５月	1995年５月
再審査結果	2001年12月	
国際誕生	1985年４月	

〔貯　　法〕室温保存
PTP包装はアルミ袋開封後、バラ包装は開栓後湿気を避けて保存すること。
〔使用期限〕外箱又はラベルに表示の使用期限内に使用すること。
注）注意－医師等の処方箋により使用すること

【禁　　忌】（次の患者には投与しないこと）
本剤の成分に対し過敏症の既往歴のある患者

*【組成・性状】

1. 組成

錠３mg：本剤は、１錠中にブナゾシン塩酸塩３mgを含有する白色徐放性のフィルムコーティング錠である。添加物としてエチルセルロース、クロスカルメロースナトリウム、軽質無水ケイ酸、酸化チタン、ショ糖脂肪酸エステル、ステアリン酸カルシウム、精製セラック、タルク、中鎖脂肪酸トリグリセリド、乳糖水和物、ヒドロキシプロピルセルロース、ヒプロメロース、マクロゴール6000を含有する。

錠６mg：本剤は、１錠中にブナゾシン塩酸塩６mgを含有する白色徐放性のフィルムコーティング錠である。添加物としてエチルセルロース、クロスカルメロースナトリウム、軽質無水ケイ酸、酸化チタン、ショ糖脂肪酸エステル、ステアリン酸カルシウム、精製セラック、タルク、中鎖脂肪酸トリグリセリド、乳糖水和物、ヒドロキシプロピルセルロース、ヒプロメロース、マクロゴール6000を含有する。

***2. 製剤の性状**

販売名	剤形<br>識別コード	外形			性状
		表	裏	側面	
デタントールR錠3mg	フィルムコーティング錠	デタントールR 3	デタントールR 3		白色
	—注1)	直径(mm)・質量(mg)・厚さ(mm)<br>5.6	83	3.1	
デタントールR錠6mg	フィルムコーティング錠	デタントールR 6	デタントールR 6		白色
	—注2)	直径(mm)・質量(mg)・厚さ(mm)<br>7.2	166	3.9	

注1）「デタントールR３」の印字あり
注2）「デタントールR６」の印字あり

【効能・効果】

高血圧症

【用法・用量】

通常、成人にはブナゾシン塩酸塩として１日１回３～９mgを経口投与する。ただし、１日１回３mgから開始し、１日最高投与量は９mgまでとする。

translation: 3 - 9 mg/day

【使用上の注意】

1. 慎重投与（次の患者には慎重に投与すること）

(1)肝障害のある患者
〔本剤は主として肝で抱合を受けて糞中に排泄されるので、肝機能の低下している患者では血中濃度が上昇するおそれがある。〕
(2)腎機能障害のある患者
〔腎機能障害者では最高血中濃度が上昇することがある。「薬物動態」の項３参照〕
(3)高齢者
〔「高齢者への投与」の項参照〕
(4)ホスホジエステラーゼ５阻害作用を有する薬剤を投与中の患者（「相互作用」の項参照）

2. 重要な基本的注意

(1)投与初期又は用量の急増時等に起立性低血圧に基づく立ちくらみ、めまい等があらわれることがあるので、高所作業、自動車の運転等危険を伴う作業に従事する人には注意を与えること。
(2)起立性低血圧があらわれることがあるので、臥位のみならず立位又は坐位で血圧測定を行い、体位変換による血圧変化を考慮し、坐位にて血圧をコントロールすること。
(3)投与初期又は用量の急増時等に立ちくらみ、めまい、悪心、また、胸部不快感、呼吸困難等があらわれることがある。その際は仰臥位をとらせるなどの適切な措置を講ずる。また必要に応じて、患者の合併症、既往歴等を十分に考慮のうえ、昇圧剤の投与等の対症療法を行うこと。

3. 相互作用

併用注意（併用に注意すること）

薬剤名等	臨床症状・措置方法	機序・危険因子
利尿剤<br>他の降圧剤	作用が増強されるおそれがあるので、減量するなど注意すること。	相加的な降圧作用の増強による。
リファンピシン	本剤の作用が減弱することがある。	相手薬剤の肝薬物代謝酵素誘導により、本剤の血中濃度が低下することがある。
ホスホジエステラーゼ５阻害作用を有する薬剤<br>バルデナフィル塩酸塩水和物<br>シルデナフィルクエン酸塩等	併用により症候性低血圧があらわれるとの報告がある。	相手薬剤の血管拡張作用により、本剤の降圧作用が増強されるおそれがある。

（裏面につづく）

4. 副 作 用

総症例3,817例中、223例（5.84%）の副作用が報告されている。（再審査終了時）

(1)重大な副作用

失神、意識喪失　失神（0.1～5％未満）、意識喪失（0.1％未満）（多くは一過性の血圧低下による）があらわれることがあるので、そのような場合には本剤の投与を中止し、仰臥位をとらせるなど適切な処置を行うこと。

(2)その他の副作用

	0.1～5％未満	0.1％未満	頻度不明
**精神神経系**	めまい、頭痛、不眠、倦怠感、眠気、耳鳴、頭重	しびれ感、意識低下、脱力感	
**循環器**	立ちくらみ、動悸、頻脈、低血圧	胸部圧迫感、胸部不快感、起立性低血圧	
**消化器**	悪心	嘔吐、食欲不振、胃部不快感、下痢、口渇、便秘	腹痛
**肝臓**		AST（GOT）、ALT（GPT）、γ-GTP上昇等	
**泌尿器**	頻尿	夜間尿、尿失禁	
**過敏症**注)	発疹	瘙痒	
**その他**	顔面潮紅、浮腫、のぼせ	肩こり、発汗、かすみ目	鼻閉、息苦しさ

注）このような場合には投与を中止すること。

5. 高齢者への投与

高齢者には、次の点に注意し、少量（3 mg／日）から開始するなど患者の状態を観察しながら慎重に投与すること。

(1)高齢者では、一般に過度の降圧は好ましくないとされている（脳梗塞が起こるおそれがある）。

(2)高齢者では、肝・腎機能が低下していることが多く、また体重が少ない傾向があるなど副作用が発現しやすい。（腎機能障害のある患者については「薬物動態」の項3参照）

(3)高齢者では、十分に経過観察を行い、慎重に増量するなど注意すること。なお、過度の降圧が認められた場合には、減量又は投与を中止するか、他の降圧剤への変更を考慮すること（「重要な基本的注意」の項参照）。

6. 妊婦、産婦、授乳婦等への投与

(1)妊婦又は妊娠している可能性のある婦人には、治療上の有益性が危険性を上回ると判断される場合にのみ投与すること。

〔動物実験（ラット）で催奇形作用が報告されている。〕

(2)授乳中の婦人に投与する場合には授乳を中止させることが望ましい。

〔動物実験（ラット）で乳汁中への移行が報告されている。〕

7. 小児等への投与

小児に対する安全性は確立していない（使用経験がない）。

8. 適用上の注意

(1)服用時

本剤をかみくだいて服用すると、一過性の血中濃度の上昇に伴って副作用が発生しやすくなるおそれがあるため、本剤はかまずに服用させること。

(2)薬剤交付時

PTP包装の薬剤はPTPシートから取り出して服用するよう指導すること。（PTPシートの誤飲により、硬い鋭角部が食道粘膜に刺入し、更には穿孔をおこして縦隔洞炎等の重篤な合併症を併発することが報告されている）

9. その他の注意

(1)類似化合物（プラゾシン塩酸塩）で腎及びその他の動脈狭窄、脚部及びその他の動脈瘤等の血管障害のある高血圧患者で、急性熱性多発性関節炎がみられた1例報告がある。

(2)α_1遮断薬を服用中又は過去に服用経験のある患者において、α_1遮断作用によると考えられる術中虹彩緊張低下症候群（Intraoperative Floppy Iris Syndrome）があらわれるとの報告がある。

【薬物動態】

1. 血中濃度

(1)健康成人における生物学的利用性

健康成人男子（12名）にデタントール錠1 mg 2錠及びデタントールR錠6 mg 1錠を経口投与し血漿中濃度推移を比較した結果、デタントールRの相対的生物学的利用率は81.1％であり、平均滞留時間（MRT）から持続性を有することが示された。（①）

(2)食事効果

健康成人男子（12名）にデタントールR錠6 mg 1錠を空腹時及び食後経口投与した結果、食事による影響は認められなかった。（①）

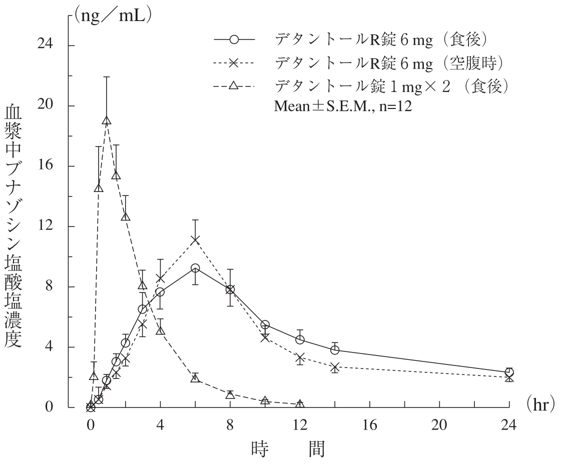

ブナゾシン塩酸塩単回経口投与時の血中濃度推移

ブナゾシン塩酸塩単回経口投与時の薬物動態パラメータ

製剤 投与条件	C_{max} (ng／mL)	t_{max} (hr)	AUC (ng･hr／mL)	R.B.A. (%)	MRT (hr)
デタントールR錠 6.0mg(食後)	10.19±1.10	5.25±0.54	132.73±15.42 2)	81.07±5.98	13.02±0.66
デタントールR錠 6.0mg(空腹時)	11.38±1.32	6.00±0.35	123.03±16.50 2)	74.05±5.24	12.77±0.66
デタントール錠 2.0mg(食後)	22.48±2.41	0.96±0.16	54.68±5.72 1)	100.00±0.00	2.60±0.15

R. B. A.：Relative bioavailability（相対的生物学的利用率）
MRT：Mean residence time（平均滞留時間）
1) $AUC_{(0\text{-}\infty)}$、2) $AUC_{(0\text{-}48h)}$　　n＝12, Mean±S. E.M.

2. 生物学的同等性

健康成人（24名）を対象にデタントールR錠3 mgと同錠6 mgの生物学的同等性を検討した。ブナゾシン塩酸塩として6 mgを各々1回経口投与し、血漿中濃度推移を比較したところ両製剤は同等であった。

3. 腎機能障害患者における体内動態

高血圧症患者（腎機能正常〔NRF〕6名、腎機能障害〔IRF〕5名）にデタントールR錠6 mgを1日1回8日間反復経口投与したとき、投与初日ならびに8日目において、腎機能障害患者では腎機能正常患者と比較して、最高血漿中濃度の上昇が認められた。（②）

NRF群、IRF群のデタントールR錠 6 mg単回、反復投与時の薬物動態パラメータ

	C_{max}(ng／mL)		t_{max}(hr)		$AUC_{0\sim24}$(ng・hr／mL)	
	NRF群	IRF群	NRF群	IRF群	NRF群	IRF群
単回投与 NRF群 vs IRF群	$p<0.05$		N.S.		N.S.	
単回投与	10.1±2.0	15.9±1.2	3.7±0.3	4.4±1.0	115.5±20.2	184.1±25.4
単回投与 vs 反復投与	N.S.	N.S.	N.S.	N.S.	N.S.	N.S.
反復投与	10.7±2.3	19.5±1.9	6.0±1.4	4.0±1.1	138.4±32.4	246.4±49.0
反復投与 NRF群 vs IRF群	$p<0.05$		N.S.		N.S.	

Mean±S.E.M., n＝ 5 ～ 6
単回投与と反復投与はpaired *t*-test
NRF群とIRF群はStudent's *t*-test

【臨床成績】

二重盲検比較試験を含む総症例466例の臨床試験成績の概要は次のとおりである（降圧効果はいずれも下降以上であり、判定不能例を除く）。（③④⑤⑥⑦）

1. 軽症・中等症本態性高血圧症

軽症・中等症の本態性高血圧症に対する降圧効果は63.2%（225／356例）であった。本剤単独投与と他剤併用投与を比較した試験では、単独投与例の降圧効果は49.4%（38／77例）、利尿薬併用例73.7%（28／38例）、β遮断薬併用例57.9%（11／19例）であった。6ヵ月以上の長期投与例においても安定した降圧効果を示した。また、二重盲検比較試験では、デタントールR（3～9 mg、1日1回）とデタントール（1.5～6 mg、1日3回分服）を比較した結果、同等の降圧効果、有用性が認められた。（③④⑤）

2. 重症高血圧症

重症高血圧症に対する降圧効果は80.6%（25／31例）であった。（⑥）

3. 腎障害を伴う高血圧症

腎障害を伴う高血圧症に対する降圧効果は69.2%（18／26例）であった。（⑦）

【薬効薬理】

1. 心血管系のα_1受容体を選択的に遮断する

(1)ラット輸精管を用いた*in vitro*の実験で、ブナゾシン塩酸塩はα_1受容体を選択的に遮断し、α_2受容体には影響を与えない。このため、交感神経末端のα_2受容体を介するノルアドレナリンのネガティブ・フィードバック機構を阻害しないため、ノルアドレナリンの過剰放出を起こさない。（⑧）

(2)モルモット腸間膜動脈及び腸間膜静脈を用いた*in vitro*の実験で、ブナゾシン塩酸塩はα_1受容体を選択的に遮断し、高濃度でもα_2受容体には影響を与えない。（⑨）

2. 末梢血管抵抗を減少し、降圧作用を示す

ブナゾシン塩酸塩は末梢血管のα_1受容体を選択的に遮断し、血管を拡張させ、自然発症高血圧ラット、DOCA・食塩高血圧ラット、腎性高血圧イヌにおいて降圧作用を示す。

さらにブナゾシン塩酸塩は、降圧に伴う生体反応である体液性昇圧因子を増加させない。（⑩⑪⑫）

【有効成分に関する理化学的知見】

一 般 名：ブナゾシン塩酸塩（Bunazosin Hydrochloride）
化 学 名：4-Amino-2-(4-butanoyl-1, 4-diazepan-1-yl)-6, 7-dimethoxyquinazoline monohydrochloride
分 子 式：$C_{19}H_{27}N_5O_3 \cdot HCl$
分 子 量：409.91
構 造 式：

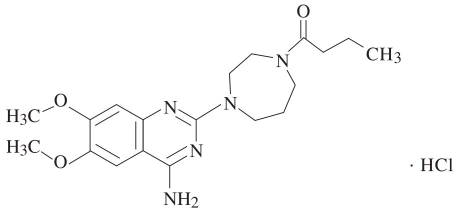

物理化学的性状：
ブナゾシン塩酸塩は白色の結晶性の粉末である。本品はギ酸に極めて溶けやすく、水又はメタノールに溶けにくく、エタノール（99.5）に極めて溶けにくく、ジエチルエーテルにほとんど溶けない。
融　　点：約273℃（分解）
分配係数：0.215（水-1-オクタノール系）

**【包　　装】

デタントールR錠 3 mg･･･100錠(PTP)･140錠(PTP14T×10)
500錠(PTP･バラ)･700錠(PTP14T×50)
****デタントールR錠 6 mg**･･･100錠(PTP)･140錠(PTP14T×10)

【主要文献】

文献請求番号

①	朝野芳郎ら：薬理と治療, **20**, 4939（1992）	DTA-0347
②	塩之入洋ら：基礎と臨床, **27**, 2211（1993）	DTA-0366
③	石井當男ら：基礎と臨床, **26**, 5047（1992）	DTA-0356
④	蔵本　築ら：基礎と臨床, **27**, 1605（1993）	DTA-0368
⑤	金子好宏ら：基礎と臨床, **26**, 5379（1992）	DTA-0363
⑥	石井當男ら：基礎と臨床, **26**, 5349（1992）	DTA-0364
⑦	武田忠直ら：基礎と臨床, **26**, 5365（1992）	DTA-0365
⑧	Shoji, T.：Jpn. J. Pharmacol., **31**, 361（1981）	DTA-0050
⑨	Suzuki, H. et al.：Gen. Pharmacol., **18**, 171（1987）	DTA-0176
⑩	Igarashi, T. et al.：Jpn. Circ. J., **41**, 903（1977）	DTA-0041
⑪	南　勝ら：基礎と臨床, **18**, 2849（1984）	DTA-0048
⑫	南　勝ら：基礎と臨床, **19**, 6972（1985）	DTA-0160

【文献請求先・製品情報お問い合わせ先】

エーザイ株式会社　hhcホットライン
フリーダイヤル　0120-419-497

製造販売元　Eisai　エーザイ株式会社
東京都文京区小石川4-6-10

SG33137-7
